# Dynamic Modelling of Pathways to Cellular Senescence Reveals Strategies for Targeted Interventions

**DOI:** 10.1371/journal.pcbi.1003728

**Published:** 2014-08-28

**Authors:** Piero Dalle Pezze, Glyn Nelson, Elsje G. Otten, Viktor I. Korolchuk, Thomas B. L. Kirkwood, Thomas von Zglinicki, Daryl P. Shanley

**Affiliations:** 1 Institute for Ageing and Health, Newcastle University, Campus for Ageing and Vitality, Newcastle upon Tyne, United Kingdom; 2 Centre for Integrated Systems Biology of Ageing and Nutrition, Institute for Ageing and Health, Newcastle University, Newcastle upon Tyne, United Kingdom; University of Chicago, United States of America

## Abstract

Cellular senescence, a state of irreversible cell cycle arrest, is thought to help protect an organism from cancer, yet also contributes to ageing. The changes which occur in senescence are controlled by networks of multiple signalling and feedback pathways at the cellular level, and the interplay between these is difficult to predict and understand. To unravel the intrinsic challenges of understanding such a highly networked system, we have taken a systems biology approach to cellular senescence. We report a detailed analysis of senescence signalling via DNA damage, insulin-TOR, FoxO3a transcription factors, oxidative stress response, mitochondrial regulation and mitophagy. We show *in silico* and *in vitro* that inhibition of reactive oxygen species can prevent loss of mitochondrial membrane potential, whilst inhibition of mTOR shows a partial rescue of mitochondrial mass changes during establishment of senescence. Dual inhibition of ROS and mTOR *in vitro* confirmed computational model predictions that it was possible to further reduce senescence-induced mitochondrial dysfunction and DNA double-strand breaks. However, these interventions were unable to abrogate the senescence-induced mitochondrial dysfunction completely, and we identified decreased mitochondrial fission as the potential driving force for increased mitochondrial mass via prevention of mitophagy. Dynamic sensitivity analysis of the model showed the network stabilised at a new late state of cellular senescence. This was characterised by poor network sensitivity, high signalling noise, low cellular energy, high inflammation and permanent cell cycle arrest suggesting an unsatisfactory outcome for treatments aiming to delay or reverse cellular senescence at late time points. Combinatorial targeted interventions are therefore possible for intervening in the cellular pathway to senescence, but in the cases identified here, are only capable of delaying senescence onset.

## Author Summary

Ageing is characterised by a gradual loss of homeostasis within organs, which is known to be driven by the accumulation of senescent cells. Cellular senescence helps prevent cells from becoming cancerous, but their detrimental effect on organ function becomes debilitating once they accumulate. These cells are particularly difficult for the body to remove, and therefore understanding what controls their survival and interactions within the organ is important to combat age-related disease. We present a mathematical model for cellular senescence. This model is used for predicting drug interventions for restoring function in cellular senescence. Whilst these interventions were predicted and tested *in vitro*, showing improved function and phenotype, none was able to restore cells to a pre-senescent state. Our model includes mitochondria, the power-plants of the cell, and we identify impairment of their turnover coupled with increased mitochondrial biogenesis as a mechanism which explained the long-term failure in drug intervention. Finally, we predict that the system dynamics stabilise in a new late-senescence state, characterised by limited network response to treatment and increased system vulnerability. This study shows formally for the first time the dynamics of cellular senescence as a system network and proves the requirement of early intervention in order to delay cellular senescence.

## Introduction

Cellular senescence, characterised by permanent cell cycle arrest, plays an important but complex role both in ageing and cancer. Like apoptosis, cell senescence is a cell-autonomous tumour protection mechanism [1], [2]. Senescent cells can persist in tissues for long periods of time and at relatively high frequencies [3]–[5]. They secrete bioactive peptides [6], [7], generate and release reactive oxygen species (ROS) [8], [9], which can promote tumourigenicity and metastasis in adjacent cells with disabled cell cycle checkpoints [10], [11]. The same mechanisms generate DNA damage, enhanced ROS production and senescence in somatic bystander cells [12], [13]. Thus, senescent cells may contribute to loss of tissue homeostasis with ageing. It has been shown that targeted ablation of senescent cells significantly delayed age-associated loss of function in multiple tissues in a progeria mouse model [14]. Furthermore, suppression of pro-inflammatory and pro-oxidant signals emanating from senescent cells while maintaining cell cycle arrest (i.e. the cell-autonomous tumour suppressor function of senescence) shows promise as a future anti-ageing intervention in humans [15]. To deliver on this promise, an improved understanding of the complex interactions between the multiple dysfunctional cellular processes that govern senescence is needed.

The most central of these processes involves accumulation of DNA damage and increased levels of ROS [8]. ROS arise mainly from mitochondrial activity, being generated as a by-product of energy production [16], and ROS-induced damage affects multiple cellular constituents and functions. Importantly, ROS damage mitochondrial DNA (mtDNA) and so impair mitochondrial function [17], [18]. In response to damage, numerous signalling pathways are activated and these are often reinforced through feedback loops [6], [9], [19]. Oxidative stress activates c-Jun N-terminal kinase (JNK) [20], which is responsible for FoxO3a translocation to the nucleus via phosphorylation [21], [22]. This then promotes transcription of the genes CDKN1A and CDKN1B, which control cell cycle arrest [21], [23]. Nuclear FoxO3a also expresses the genes LC3, Gabarapl1 and Atg12 [24], [25] inducing autophagy, a cellular recycling process which is negatively controlled by the insulin/TOR signalling pathway [26]. Inhibition of insulin signalling, particularly Akt activity, promotes transcription of FoxO3a genes (Daf-16 in *C. elegans*) and extends lifespan in worms [27], [28]. Inhibition of TOR (by caloric restriction or pharmacological intervention) and activation of AMPK (by resveratrol or metformin treatment) increase autophagy by regulating ULK1 [29]–[31]. AMPK, in cooperation with SIRT1, increases transcription and modulation of FoxO3a and PGC-1α [32]–[34], while PGC-1α was also found, in addition to the effects of AMPK activation [35], to be controlled via mTORC1 [36], [37]. Once activated, PGC-1α promotes mitochondrial biogenesis, increasing total mitochondrial mass and mitochondrial membrane potential (ψm) [38]. This leads to an increase in cellular energy production (as ATP), and possibly also to increased ROS generation.

As may be seen from this brief summary of the interconnected pathways associated with cellular senescence, there is a need not only to draw together the various elements into an integrated framework, but also to do so in a way that allows the quantitative dynamics to be examined and eventually understood. This is a challenge that necessitates a systems-biology approach. Although individual actions and reactions within the network have been established by targeted experiments, the dynamic properties of the system as a whole are more complicated than can properly be understood without the aid of mathematical modelling. In particular, modelling is likely to prove essential for identifying the consequences of more complex interventions than alterations to single elements, and thus for exposing possible paths for novel therapies against the many age-related conditions to which cellular senescence contributes.

We describe here the development of a comprehensive dynamical model of irradiation-induced cellular senescence. Using this model, we studied ROS-mTOR-dependent mechanisms of restoring mitochondrial phenotype and function. We first predicted *in silico* and then verified *in vitro* that it is possible to improve the functional health of mitochondria either by scavenging ROS or inhibiting mTOR, as single interventions. The model next enabled investigation *in silico* of multiple, simultaneous parameter perturbations, making it possible to probe in detail the ‘state space’ of the senescent phenotype. From this we identified two possible interventions which should improve the cellular state. The first was the use of combined inhibition of ROS and mTOR. We tested this *in vitro* and confirmed that the dual inhibition resulted in improved mitochondrial status and reduced DNA damage. The second was the use of combined activation of AMPK and mitophagy. Again, this improved cellular function and identified the cellular control of energy status and turnover as being integral to controlling the induction (or avoidance) of senescence. Whilst combined interventions improved mitochondrial function, none were able to restore it to a pre-senescent state. Using sensitivity analysis, we identified that mitochondrial autophagy (mitophagy) affected new mitochondria but not old mitochondria, suggesting a decrease in mitochondrial fission over time. The impairment in mitochondrial turnover in combination with increased mTORC1-dependent mitochondrial biogenesis provides a theoretical explanation for the global mitochondrial mass increase in senescence. Measures of mitochondrial fusion and fission validated this finding in senescent cells. Interestingly, in all interventions, we also detected a gradual loss in treatment effectiveness at late time points: after 18 days post-senescence induction, all interventions became largely ineffective. We explored this finding using dynamic sensitivity analysis, and obtained novel evidence for the existence of a stable late-senescence state, characterised by poor sensitivity and high variability across the network. This points to cellular senescence being a locked, dysfunctional state which presumably exists to protect the organism against the risk that might otherwise be posed by continued proliferation of a badly damaged cell.

## Results

### Development of a dynamic model for cellular senescence

**Figure 1:**
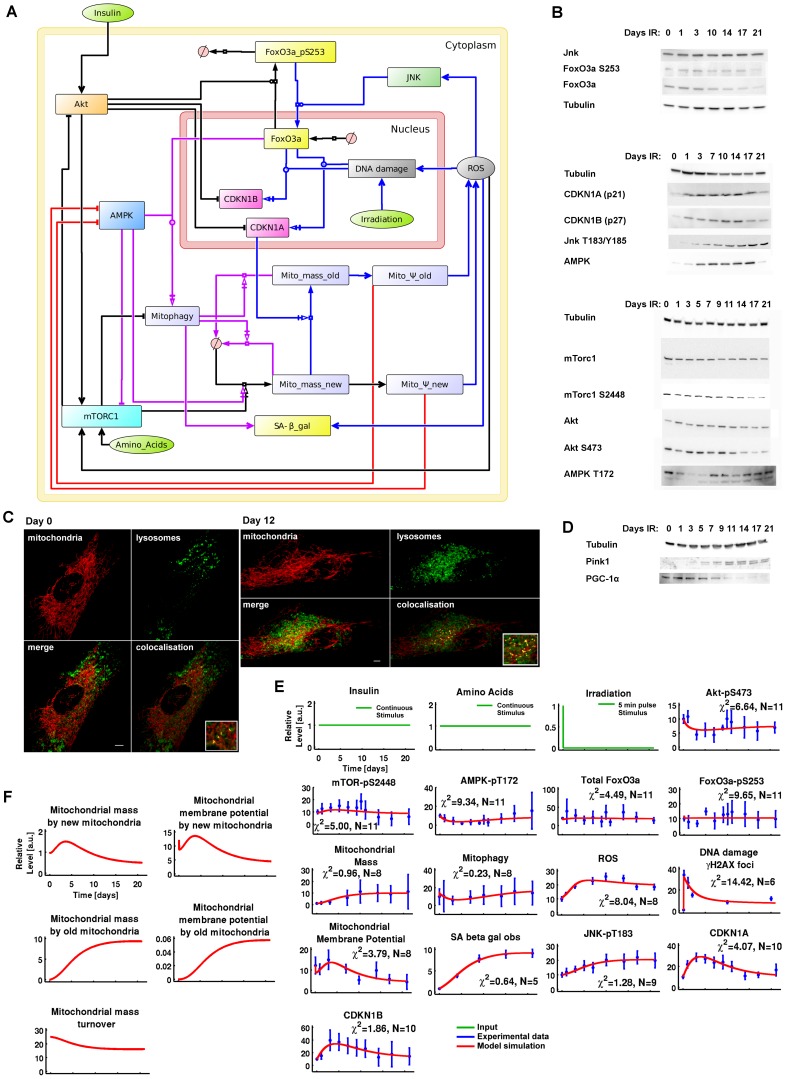
A dynamic model for cellular senescence. (A) Graphical model integrating the insulin-TOR (IIS-TOR) signalling pathway (left), the DDR-oxidative stress responses (top-right) and mitochondria (centre). The insulin-TOR signalling pathway regulated the anabolic process of mitochondrial biogenesis, FoxO3a translocation to the cytoplasm followed by ubiquitination, and inhibition of cell cycle arrest (black reactions). AMPK signalling promoted the catabolic pathway of mitophagy, but was also modelled to promote mitochondrial biogenesis (magenta reactions). Mitochondrial membrane potential (ψm) increased cellular energy levels (red reactions) deregulating AMPK, and enhanced intracellular ROS (blue reactions). DNA damage and ROS triggered a stress response and consolidated cell cycle arrest. (B) Representative western blot data used for calibrating the model. (C) Representative imaging data used for measuring co-localisation between mitochondria (COXIV) and lysosomes (LC3) at day 0 and 12. These data were used for creating a time course for mitophagy activation as indicated in panel E.(D) Additional controls for mitophagy data using Pink 1 and mitochondrial biogenesis using PGC-1α indicating correlation between mTOR-pS2448 and PGC-1α. (E) *In silico* versus *in vitro* time courses. The model (red lines) was calibrated over experimental time course data (blue points) collected for 14 readouts in the network up to 21 days. The inputs are amino acids/insulin (constant inputs) and irradiation (pulse input of 5 min which simulates 20 Gy X ray irradiation over 5 min). Experimental time points (blue points) are mean +/−1 standard deviation collected from 5 independent repetitions. N = number of data points used for fitting each readout time course. The goodness-of-fit statistical measures χ^2^ [61] and AIC [68] for this model are 70.4278 and 381.838, respectively. (F) Simulated time-courses of the internal states for mitochondrial mass, ψm and turnover.

The model presented in Figure 1A integrated five key regulators of cellular ageing: insulin-TOR, FoxO3a, DNA damage response (DDR), ROS, and mitochondrial function. The insulin and insulin-like growth factor 1 (IIS)-TOR network was abstracted [from 39], [40] in order to represent as simply as possible (consistent with capturing the functional essence) the dynamics of Akt, mammalian TOR Complex I (mTORC1) and the mTORC1-p70-S6K-induced negative feedback loop. The mammalian TOR Complex II (mTORC2) was not included explicitly but is represented in its contribution to Akt-pS473, which is a readout for both the mTORC1-p70-S6K-negative feedback and insulin-mTORC2 activity, independent of the negative feedback loop [39]. For FoxO3a, we modelled the main processes of synthesis governed by an activated DNA damage response (via ATM interactions), translocation from the nucleus to the cytoplasm, subsequent ubiquitination as regulated by Akt, and an opposing translocation regulated by JNK activity [21], [22]. The dynamics of Akt-pT308 and Akt-pS473 overlap, and by selecting Akt-pS473 we could model both the Akt-pS473-dependent regulation of FoxO3a and the Akt-pT308-dependent activation of mTORC1 in one step. Cell cycle arrest was followed in the model via the cell division kinase inhibitors 1A (CDKN1A, also known as p21) and 1B (CDKN1B, also known as p27), whose levels were regulated negatively via Akt [41] and positively via the transcriptional activity of FoxO3A and DNA damage/p53. The nutrient (amino acid)–dependent regulation of mTORC1 was also included along with the energy-dependent regulation of AMPK via mitochondrial function. mTORC1 was inhibited by AMPK indirectly via TSC2 phosphorylation [42] and directly via Raptor phosphorylation [43]. mTORC1 and AMPK independently triggered mitochondrial biogenesis via PGC-1α [35], [36]. In conjunction with FoxO3a activation, AMPK was implemented to induce mitophagy [25].

Mitochondrial mass and function play important roles in cell senescence: driven by signalling downstream of CDKN1A [9], [17] and mTOR [44], mitochondrial mass per cell increases in senescence. This is associated with decreased mitochondrial membrane potential, ψm, and increased production of ROS, together indicating mitochondrial dysfunction in senescence [17]. Increased ROS generate more DNA damage, driving a positive feedback loop by maintaining activation of CDKN1A [9]. To quantitatively model this, we added a mitochondrial module which describes the mitochondrial population in a cell as two subpopulations, ‘new’ and ‘old’. Biogenesis only generates ‘new’ mitochondria, which then develop (or degenerate) to ‘old’ mitochondria with the rate of conversion dependent on CDKN1A. ‘New’ mitochondria are different from ‘old’ in terms of amounts in the cell, membrane potential (and thus propensity for ATP generation), ROS production and probability for mitophagy. Under basal conditions, there are essentially only new mitochondria in a cell. Total mitomass and membrane potential are the sum of both subpopulations.

Finally, ROS were represented as inducing DNA damage and activating JNK and mTORC1 (via ATM to TSC2 and/or IKK-β to TSC1). In conjunction with mitophagy, ROS drove accumulation of lipofuscin [45] and therefore an increase in senescence-associated β-galactosidase (SA-β-gal).

### Time-course analysis upon irradiation-induced senescence

#### *In vitro* data collection

To calibrate the model, MRC5 fibroblasts were treated with X-ray irradiation (20 Gy), which results in an irreversible cell cycle arrest and eventual acquisition of a senescence phenotype [46]. *In vitro* experimental time course data were collected up to 21 days after X-ray irradiation and analysed by western blot for protein levels and phosphorylation (Figure 1B). Immunofluorescence co-localisation of LC3 and CoxIV were used to determine a time course for mitochondrial degradation via mitophagy (representative images in Figure 1C). Pink1 western blotting (Figure 1D), plus controls treated with Bafilomycin A followed by live cell microscopy measuring colocalization of mitochondrially targeted RFP with lysosomal GFP (Figure S1) were used to further validate quantified data on LC3-CoxIV co-localisation (Figure 1E). We also detected PGC-1α (Figure 1D), which reported a consistent correlation with mTOR-pS2448, in agreement with previously published data showing mTORC1-dependent PGC-1α activation [36], [37]. Therefore, we condensed PGC-1α-dependent mitochondrial biogenesis regulation into mTOR-pS2448-dependent signalling to reduce the model complexity. DNA damage levels were determined by γH2A.X immunofluorescence (Figure 1E). Live cell microscopy was used for detecting mitochondrial mass and ψm, and flow cytometry for ROS levels. As an additional control, mitochondrial mass was also detected and confirmed using flow cytometry (Figure S2). Data for SA-β-gal were taken from previously published data [9]. Finally, since our cell viability data were constant along the time course (Figure S3), we decided to omit an apoptosis module in the model. A table containing the complete quantitative data set used to estimate the model parameters is provided in Table S1.

#### *In vitro* and *in silico* time-course analysis

These 14 readouts were used to estimate and identify the parameters of the model introduced in Figure 1A (see Materials and Methods, Tables S2, S3, S4 and Figures S4, S5, S6, S7, S8, S9, S10, S11, S12, S13, S14, S15, S16, S17, S18). The model simulation versus *in vitro* quantification data upon X-ray irradiation is shown in Figure 1E. Following irradiation, *in vitro* and *in silico* data showed a dramatic increase in DNA damage foci followed by persistent oxidative stress and inflammatory responses (ROS, JNK-pT183 and SA-β-gal) and consolidated cell cycle arrest as shown by increased CDKN1A and CDKN1B levels as well as reduced Akt-pS473 phosphorylation. Interestingly, mTOR-pS2448 levels increased upon irradiation until days 7–10 and decreased thereafter. Consistent with mTOR-pS2448 activation, new mitochondria were created by mitochondrial biogenesis (mitochondrial mass). The mitochondrial ψm showed a gradual decrease from day 5, indicating that the mitochondrial mass from day 7 until 21 represented mitochondrial networks of low ψm. This decrease in mitochondrial ψm was responsible for the decrease in energy levels (increase in AMP/ATP ratio level) as shown by AMPK-pT172 activation. Mitophagy levels also remained low in the first 10 days and then increased, consistent with AMPK-pT172 levels and inversely with mTOR-pS2448 activity. FoxO3a-pS253 and total FoxO3a levels remained generally stable along the time course, highlighting that CDKN1A/B and mitophagy responses were governed more by DNA damage and AMPK, respectively, than by FoxO3a.

#### Model prediction

The distinction of the internal mitochondrial states, new and old, allowed us to predict three essential features for the two sub-populations of mitochondria: mass, ψm and turnover (Figure 1F). The population of new functional mitochondria was characterised by a reduced mass and a high ψm. In contrast, the population of old dysfunctional mitochondria showed an elevated mass and a low ψm. The total mitochondrial mass, associated to the *in vitro* observed mitochondrial mass (Figure 1E), was determined predominantly by the old sub-population (Figure 1F), whereas the total mitochondrial ψm (Figure 1E), was largely due to the new sub-population (Figure 1F). Throughout the time course, the model predicted a gradual decrease in mitochondrial turnover, indicating an inability to correctly eliminate old dysfunctional mitochondria over time. The next step was to use our dynamic mTOR-mitochondria-ROS model to predict *in silico*, and then test *in vitro*, modalities for restoring mitochondrial function in cellular senescence.

### ROS inhibition enhanced mitochondrial membrane potential

#### Background

As ROS is a central driver for mitochondria dysfunction we gradually inhibited the variable ROS in the model and analysed the effects on mitochondrial ψm along the time course. As the model species associated to ROS is produced and destroyed dynamically, we inhibited the ROS species continuously. To achieve this, we created a simulated ROS inhibitor able to reduce ROS levels by up to 90% of the control level along the time course (Figure S19A). Gradual levels of inhibition from 0% (control) to 90% were also simulated.

#### Model prediction

**Figure 2:**
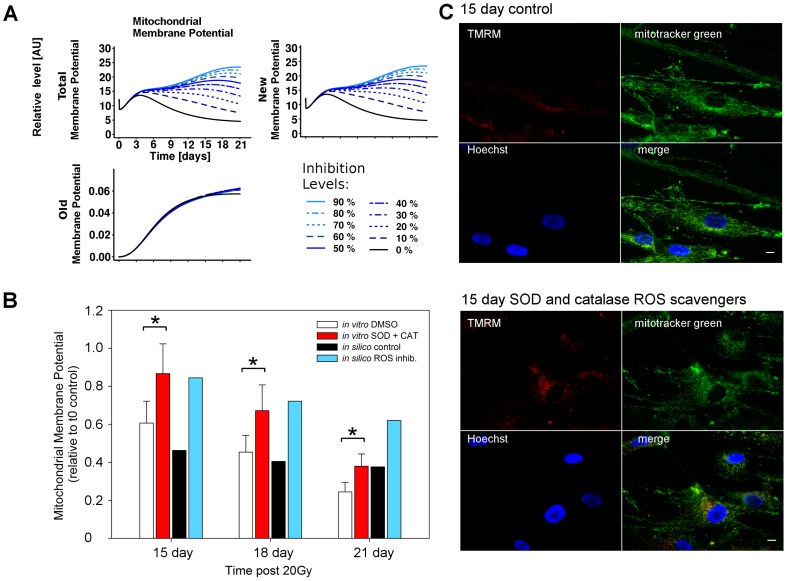
ROS inhibition increased mitochondrial membrane potential. (A) Simulated time-courses for mitochondrial membrane potential (ψm). Gradual ROS inhibition from 0% (black, control) to 90% predicted an increase in mitochondrial ψm due to perturbation of the new mitochondrial population. (B) The model prediction was confirmed by measuring mitochondrial membrane potential by live cell imaging and quantifying the fluorescence intensities (n = 3). Exogenous addition of SOD and catalase significantly increased the average ψm (Mann-Whitney test, * P<0.05) *in vitro*. *In silico* inhibition of ROS levels also partially reactivated mitochondrial ψm in a dose dependent manner, with between 15 and 30% levels giving equivalent restoration of ψm to the *in vitro* data. *In vitro* mitochondrial ψm was determined in MRC5 cells 15, 18 and 21 days post IR using live cell imaging of cells loaded with the mitochondrial ψm dependent dye TMRM and non-potential dependent mitotracker green. (C) Example images of data used in (B) for control cells (upper panel) and cells treated with SOD and catalase (100 U each) in the medium (lower panel) for 15 days post IR, stained with the mitochondrial ψm dependent dye TMRM, the non-potential dependent dye mitotracker green, and the nuclear counterstain Hoechst 33342. Scale bar is 10 µm.

Upon ROS inhibition the model predicted an increase in the total mitochondrial ψm after three days post-irradiation. Interestingly, this increase was explained by the model as being dependent on the new mitochondria population (Figure 2A). Despite the ψm increase, the model also predicted that only high levels of ROS inhibition (>70%) were able to maintain high levels of mitochondrial ψm at later time points (>9 days). In contrast, milder levels of inhibition gradually lost their effectiveness over time. This suggested that ROS inhibition (Figure S19) could increase the mitochondrial ψm but was not sufficient to restore mitochondrial function, which was gradually overwhelmed with time.

#### *In vitro* testing

These predictions were experimentally tested *in vitro* by treating cells with exogenous ROS-scavenging enzymes superoxide dismutase and catalase (SOD and CAT) throughout the time course. This treatment had the effect of decreasing cellular superoxide production throughout the timecourse (Figure S20A). By measuring ψm (via TMRM/MTG ratio) at days 15, 18 and 21 post-irradiation we observed a significant increase in treated cells (Figure 2B and 2C). By matching *in silico* to *in vitro* data, an increase in the TMRM/MTG ratio of 25–40% was found to correspond to ROS-inhibition of 15–30% in the model, confirming the model prediction that ROS levels impinge upon mitochondrial membrane potential. Furthermore, as predicted by the model, differences in ψm between SOD/CAT-treated and control cells became smaller with time (Figure 2B) despite non-diminishing efficiency of the treatment (Figure S20).

### mTOR inhibition reduced mitochondrial mass

#### Background

TOR plays a crucial role in the regulation of autophagy [30], [31], mitochondrial biogenesis [36] and Akt feedback [47], [48]. Hence, we studied the consequences of TOR inhibition on mitochondrial mass over the time course. Perturbation of mTORC1 alone produces undesired effects in the insulin/TOR signalling pathway: it reduces mTORC1-p70-S6K-dependent negative feedback to the insulin receptor substrate (IRS) and therefore hyperactivates Akt [49], [50]. A cleaner approach was to perturb both mTOR complexes simultaneously by intervening with TOR kinase directly. Although mTORC2 was not included in the model, we could still approximate inhibition of TOR kinase by reducing the protein levels for both mTORC1 and Akt at the same time. Since no turnover was included for the species mTORC1 and Akt, it was sufficient to decrease their initial protein levels to model their inhibition. Using this approach, we simulated a TOR-specific inhibition, similar to Torin1 treatment [51].

#### Model prediction

**Figure 3:**
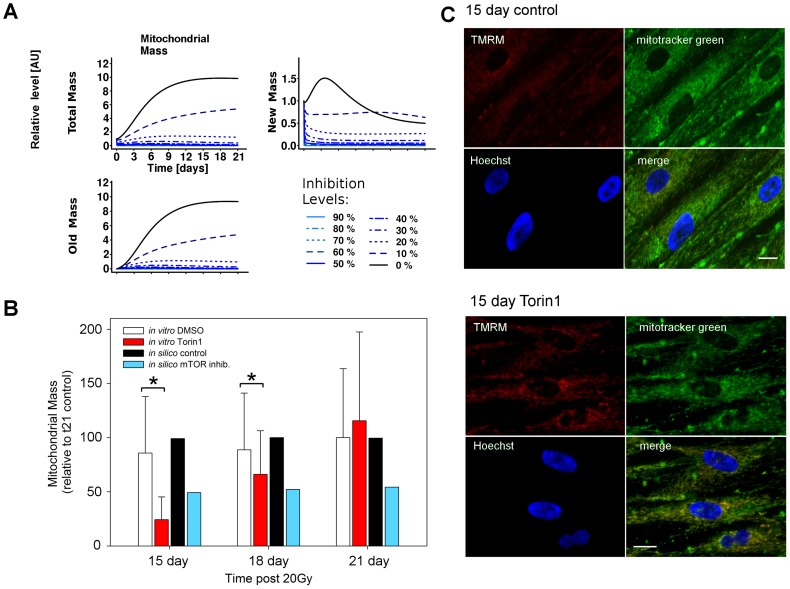
mTOR inhibition decreased mitochondrial mass. (A) Simulated time-courses for mitochondrial mass. Gradual mTOR specific inhibition from 0% (black, control) to 90% predicted a decrease in mitochondrial mass due to the perturbation of the population of old mitochondria. (B) The model prediction was confirmed by measuring mitochondrial mass using mitotracker green and quantifying the fluorescence intensities (n = 3). Exogenous addition of 10 nM Torin1 significantly decreased the average mass (Mann-Whitney test, * P<0.05) *in vitro*. *In silico* inhibition of mTOR levels also partially decreased mitochondrial mass in a dose dependent manner, with between 15 and 30% levels giving equivalent restoration of ψm to the *in vitro* data. (C) Example images of data used in (B) for control cells (upper panel) and cells treated with Torin in the medium (lower panel) for 15 days post IR, stained with the mitochondrial ψm dependent dye TMRM, the non-potential dependent dye mitotracker green, and the nuclear counterstain Hoechst 33342. Scale bar is 10 µm.

Upon mTOR inhibition the model predicted a decrease in total mitochondrial mass to be already detectable after three days post-irradiation, due primarily to a decrease in mass not only of the old mitochondrial population, but also of the new population (Figure 3A). The model also predicted that an inhibition of mTOR by more than 20% would be sufficient to maintain low mitochondrial mass levels along the time course. Since mitochondrial biogenesis affects the population of new mitochondria, inhibition of mTOR by more than 40% was predicted to be detrimental for this group, compromising healthy cellular function. A safe margin of intervention was predicted to be between 10–20% inhibition, which maintained a moderate and stable population of new mitochondria, and limited the old dysfunctional population.

#### *In vitro* testing

These predictions were tested experimentally and confirmed *in vitro* by treating cells with Torin1 (Figure S20B) and measuring mitochondrial mass (using mitotracker green) at 15, 18 and 21 days post-irradiation (Figure 3B and 3C). Although the reduction in total mitochondrial mass matched the prediction until day 18, the later time point showed high variation and no decrease of mitochondrial mass within the Torin1 treated cells. Similar to the ROS scavenging intervention, the earliest time points were most effective, displaying a gradual loss of treatment efficacy. These data suggest that until days 15–18 the cellular state change from normal to senescence remained partially transient and additional cellular dysfunction was occurring at late time points.

### Combined ROS-mTOR inhibition synergistically increased mitochondrial membrane potential

#### Background

In the previous sections, the effects of ROS or mTOR inhibition were predicted and tested, showing that the former increased mitochondrial ψm whereas the latter decreased mitochondrial mass. At this stage, we wished to investigate the outcome of a combined ROS-mTOR treatment. The aim was to detect possible non-linear synergistic effects which could further increase mitochondrial ψm and maintain a reduced population of mitochondria at later time points.

#### Model prediction

**Figure 4:**
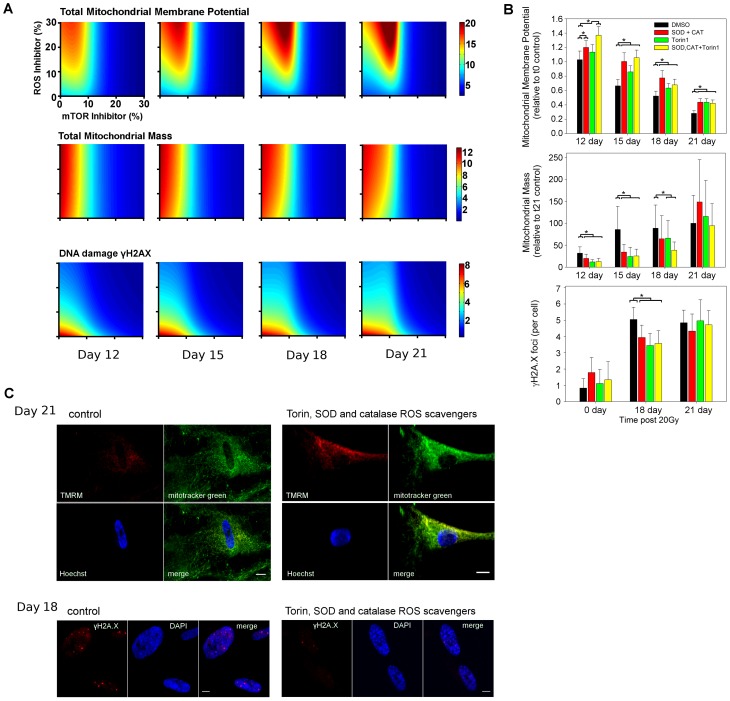
Combined mTOR-ROS inhibition increased mitochondrial membrane potential. (A) Model predictions were obtained by plotting the intensity for each readout (membrane potential (ψm), mitochondrial mass and DNA damage, respectively) with inhibition of mTOR (x axis) and ROS (y axis). The control (no inhibition) is represented as the point (0, 0). Prediction data are shown at days 12, 15, 18 and 21 post-irradiation. (B) *In vitro* ψm, mitochondrial mass and DNA damage foci number upon inhibition of ROS, TOR or combined TOR-ROS at days 12, 15, 18 and 21, or 18 and 21 (for DNA damage foci). Cells were treated with 10 nM Torin1 (TOR inhibitor), or Torin1 with SOD and catalase (100 U each) (n = 3, ANOVA with Dunn's post-hoc test, * P<0.05 within time points). (C) Example images of data used in (B) for control cells (left panels) and cells treated with Torin1, SOD and catalase in the medium (right panel) stained for ψm and mitochondrial mass at day 21 (above panel) or for DNA damage foci at day 18 (lower panel).

In exploring the perturbation space, the model confirmed ROS inhibition as an effective treatment for increasing mitochondrial ψm, but also showed that mTOR inhibition could play a role, particularly when combined with ROS inhibition (Figure 4A, mitochondrial membrane potential). The model indicated that this change in ψm was largely due to the population of new mitochondria, suggesting that these treatments were ineffective in restoring functionality in the old population (Figure S21). Interestingly, the synergy of a ROS-mTOR combined treatment further increased mitochondrial ψm as compared to single interventions (Figure 4A). Despite this, the model also predicted a shift of treatment effectiveness over time for low levels of inhibition, which suggested that the treatment doses should be increased in order to maintain a constant level of mitochondrial ψm.

The next step was the analysis of mitochondrial mass. The model predicted a reduction in mitochondrial mass due predominantly to mTOR inhibition, whereas ROS was not predicted to be a major contributor (Figure 4A, mitochondrial mass). A weak synergistic effect of ROS-mTOR combined intervention was detected at day 21 although this did not show a significant improvement when compared to a single mTOR inhibition. Interestingly, at this time point the model predicted a clear increase in mitochondrial mass upon ROS inhibition. In contrast to the mitochondrial ψm, the model predicted that these treatments affected the mitochondrial mass for the populations of both new and old mitochondria (Figure S21).

Mitochondrial dysfunction and ROS production are interconnected with nuclear DNA damage in a positive feedback loop [9]. Therefore, we also predicted and tested the effect of a combined treatment on the DNA damage response. The model showed a decrease in DNA damage foci upon ROS, mTOR or combined ROS-mTOR inhibition as compared to the control along the time course (Figure 4A, DNA damage). Despite this, a combined ROS-mTOR inhibition was not predicted to further reduce the number of DNA damage foci as compared to the other treatments unless higher doses were applied. Finally, it indicated that any treatment gradually lost effectiveness over time, as shown by a gradual shift towards the top-right corner of the plots.

#### *In vitro* testing

We experimentally tested these predictions and detected a significant increase in ψm following a combined ROS-mTOR treatment as compared to the other treatments or control at day 12 post-irradiation. Although a synergistic effect was found at 12 days, synergism was lost at later time points and none of the treatments rescued the decrease of ψm with time completely (Figure 4B), in agreement with the shift in treatment effectiveness predicted by the model. The *in vitro* tests also confirmed a gradual decrease in ψm for all applied treatments. In particular, the equivalence of all treatments at day 21 suggested a gradual loss in treatment sensitivity, in agreement with the above hypothesis that cellular pathways were still developing progressive dysfunction. See upper panel in Figure 4C for representative images of ψm for control and dual perturbation of cells at day 21. We experimentally tested the model predictions for the mitochondrial mass using a combined SOD+Cat+Torin1 treatment (Figure 4B). In agreement with the model, the *in vitro* data indicated mTOR as a main contributor for mitochondrial mass. In contrast to the model prediction, *in vitro* tests also showed a reduction in mitochondrial mass upon ROS inhibition at 12, 15 and 18 days post-irradiation, but, in agreement with the model, an increase in mitochondrial mass at day 21. This partial discrepancy between *in silico* and *in vitro* data, or within the *in vitro* time-course data, highlights the complexity of ROS signalling. We also tested the model predictions for the DNA damage by counting γH2A.X DNA damage foci for each treatment combination at day 0 (control), and at 18 and 21 days post-irradiation (Figure 4B). *In vitro* tests showed a reduction in number of damage foci for each treatment up until day 18. However, no clear improvement was detected by a combined mTOR-ROS inhibition. At day 21 none of the treatments reduced DNA damage foci frequencies, in agreement with the previously advanced hypothesis of sensitivity loss at late time points (see lower panel in Figure 4C for representative images of DNA damage foci for control and dual perturbation at day 18). Finally, we determined the downstream effect of inhibition on the appearance of the senescence marker, SA-β-gal. We found that there was a slight decrease in positive cells at early time points with all interventions, and a significant decrease for combined ROS-mTOR intervention at days 3 and 6, also with a notable decrease in intensity of staining. However, by day 11 there was no difference in positive cells between any of the treatments (Figure S22).

### Modelling predicts increased mitochondrial membrane potential following AMPK, FoxO3a or mitophagy-activating treatments

#### Background

Since the previous inhibitions negatively affect anabolic processes, we decided to test the effects on the network of single and double over-activation for protein species regulating catabolic signalling pathways. AMPK, FoxO3a and mitophagy are of particular interest in cellular senescence. Since no turnover was included for the species AMPK, it was sufficient to increase its initial protein level to achieve its over-activation. The species FoxO3a and mitophagy were dynamically produced and destroyed, so their over-activation was achieved by perturbation throughout the time course. We created two simulated activators, one for FoxO3a and one for mitophagy, aimed at increasing FoxO3a or mitophagy levels by up to 150% of the control levels along the time course (Figure S19B). Gradual levels of inhibition from 0% (control) to 150% over-activation were also simulated.

#### Model prediction

**Figure 5:**
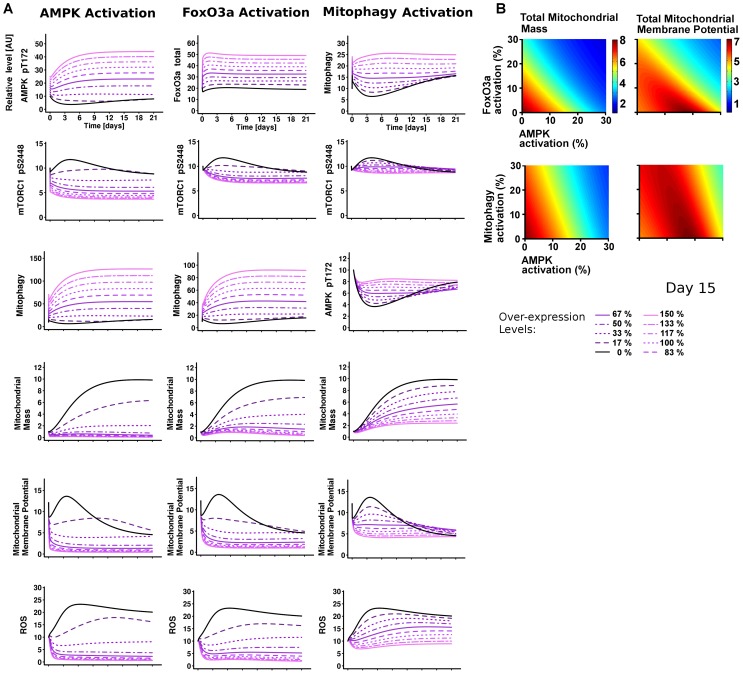
AMPK, FoxO3a, or Mitophagy simulated activations consistently improve mitochondrial function. (A) Model single perturbation of AMPK, FoxO3a, or mitophagy from 0% (control, black) to 150% gradual over-activation. (B) Model double perturbations were obtained by plotting the intensity for each readout (left, mitochondrial mass and on the right, membrane potential (ψm)) with over-activation of AMPK (x axis) and FoxO3a (y axis) (above), or AMPK (x axis) and mitophagy (y axis) (below). The control (no over-activation) is represented as the point (0, 0). Prediction data are shown at 15 days post-irradiation.

The model predicted that AMPK over-activation would reduce mitochondrial biogenesis, ψm and induce mitophagy (Figure 5A, AMPK Activation). Interestingly, the model also showed that all of the model components involved in the DDR-oxidative stress response were gradually inhibited (Figure 5 and Figure S23). Similar predictions were also found by over-activating either FoxO3a or mitophagy in the network. After analysing the effects of single perturbations, we simulated mitochondrial mass or ψm following a double over-activation of FoxO3a-AMPK, or mitophagy-AMPK at day 15 after-irradiation (Figure 5B). Interestingly, we found a synergistic protective effect of dual intervention (as shown in dark red), with lower mitochondrial mass (due to a decrease of old mitochondria) and maintained ψm. This was not obvious from the outcomes of the single perturbations. This area was mostly dependent on AMPK and, importantly, there was a rescue of ψm in association with a low mitochondrial mass. In conclusion, these results were consistent with the single and double inhibitions of ROS and mTOR, and therefore suggested that alternative therapeutic interventions were also possible and potentially effective.

### Mitochondrial dysfunction is maintained by decreased mitochondrial dynamics

#### Background

All interventions that we have tried within the context of our model have been shown to alleviate some of the symptoms of senescence, but none have been able to restore the mitochondria in senescent cells to a pre-senescent state. Therefore we performed a sensitivity analysis averaged over the time course to identify the essential reactions that, according to the model, determine mitochondrial parameters.

#### Model prediction

**Figure 6:**
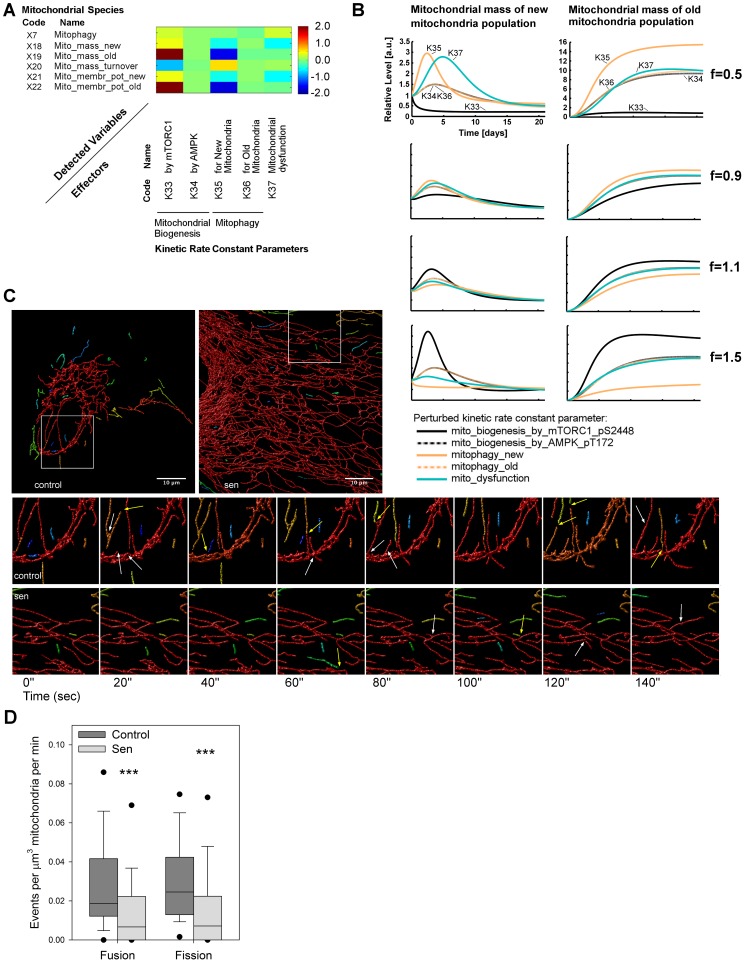
Mitochondrial dysfunction is driven by decreased mitochondrial dynamics. (A) Sensitivity analysis averaged along the time course for the mitochondrial species in the model by varying the mitochondrial-related kinetic rate constant parameters. Mitochondrial biogenesis was determined by mTORC1 signalling (k33) but not by AMPK (k34). Clearance of old mitochondria (k36) was not as effective as for new mitochondria (k35). This reduced mitochondrial quality control for the old mitochondrial population implicated an increased and sustained global mitochondrial dysfunction. Scale bar represents the normalised positive or negative sensitivity of each species to each rate constant. (B) *In silico* time courses over 21 days post-irradiation for the two states of mitochondrial mass (new and old) were computed by separately perturbing the previous kinetic rates by different factors f (f = 1 is the control). These perturbations affected the new mitochondria only at early time points (<10 days), whereas the old mitochondria were affected throughout the time course. Consistent with panel A, the perturbation of the parameters k34 and k36 (dotted lines) did not alter the mitochondrial mass readouts and are therefore overlapped. (C) Representative live cell images of mitochondrial networks in young (control) and senescent (sen) MRC5 fibroblasts. Mitochondrial networks were detected in deconvolved 3D confocal images using mitochondrially targeted fluorescent protein. Individual parts of the cell mitochondrial population are identified using colour coding. Whole frame images are shown in the upper panel, and the boxes highlight the areas (18.2 µm^2^) analysed for fusion and fission events over 30 minutes. Example images from these areas over 140 s are shown below with the detected fusion and fission events highlighted with white and yellow arrows respectively. A timelapse movie containing the full dataset from a control and senescent cell are available in Movie S1. (D) Quantification of observed fusion and fission events in 18.2 µm^2^ areas from control and senescent cells. Events were recorded every 20 s over 30 minutes for each cell and expressed per minute and relative to the mitochondrial mass in the area observed. Significant decreases were seen for both fusion and fission in senescent cells (Mann-Whitney, p<0.001, n = 6 cells per sample with between 90–180 events recorded).

Using sensitivity analysis averaged over the time course, we therefore investigated the role that each mitochondrial-related kinetic rate constant played upon the mitochondrial species within the model (Figure 6A). The two most important reactions determining mitochondrial mass and membrane potential were mTORC1-dependent mitochondrial biogenesis and mitophagy of new mitochondria. AMPK-driven biogenesis played a very minor role (kinetic rate constant k34). To our surprise, the model predicted that variable mitophagy of old mitochondria (rate constant k36) would essentially have no effect on mass and membrane potential of either young or old mitochondria. Finally, mitochondrial dysfunction (kinetic rate constant k37), describing the rate of conversion of young into old mitochondria driven by CDKN1A-p38-TGF-β signalling, only affected the new mitochondria mass species, with concomitant effects upon mitochondrial membrane potential (species ×18 and ×21).

We next investigated the time courses of these species by varying the above kinetic rate constants by factors ranging from f = 0.5 to f = 1.5. These results confirmed the sensitivity of new mitochondrial mass upon mTORC1-dependent mitochondrial biogenesis, mitophagy and (to a lesser degree) mitochondrial dysfunction (Figure 6B, solid lines). Additionally, this analysis showed that changes in these kinetic rate constants affected the new mitochondria population only at the early time points (first row, time <10 days), whereas changes in kinetic rate constants k33 (mTORC1 driven biogenesis) and k35 (mitophagy of new mitochondria) affected the old mitochondrial population throughout the time course. Interestingly, the kinetic analysis confirmed that the increase in mass of old mitochondria was almost exclusively driven by reactions (mTORC1 driven biogenesis and mitophagy) acting only on the new mitochondria, but not by mitophagy of old mitochondria themselves (Figure 6B).

We tested whether this surprising prediction could have been precipitated by an artificial constraint in the model. In the original model, mitophagy of old mitochondria was limited to be smaller than that of new mitochondria to mimic the requirement for mitochondrial fission (Table S2). However, the parameter estimation without that constraint achieved the same result as before (i.e. the value for mitophagy of old mitochondria remained much smaller than that for new mitochondria). Hence, we left that constraint in the model to minimise the computation time of the parameter estimation.

In conclusion, sensitivity analysis predicted that the accumulation of dysfunctional mitochondria in senescence would be caused by the combination of an initial hyperactive mTORC1-dependent mitochondrial biogenesis followed by impaired turnover of the mitochondrial population. While more new mitochondria would be generated, mitophagy of new mitochondria would be insufficient to degrade them before they degenerate into old mitochondria, for which mitophagy would be even less efficient, resulting in accumulation of old, dysfunctional mitochondria. Since mitophagy was functional in our system throughout the time course (Figures 1C, 1E, and S1), we were led to the hypothesis that the turnover inefficiency for the old population was due to a decrease in mitochondria fission events.

#### *In vitro* testing

Mitochondrial fission events are a pre-requisite for mitophagy. The predicted insufficiency of mitophagy might thus be related to low fission (and fusion) activity in senescent cells. To test this hypothesis, we transduced young and senescent MRC5 fibroblasts with a mitochondrially targeted fluorescent protein and followed the mitochondrial network movement over time. We detected a statistically significant decrease in the number of both fusion and fission events in senescent cells (Figure 6C and 6D) concomitant with decreased mitochondrial movement (Movie S1). Therefore, decreased mitochondrial dynamics may explain preferential targeting of young mitochondria to mitophagy as well as increased mitochondrial mass in senescence, the latter by decreasing the available pool of small mitochondria for mitophagy.

### Complications in treating late senescence: a network-wide perspective

#### Background

The previous sections indicated that therapeutic treatments aiming to reduce ROS, mTOR or combined ROS-mTOR levels could improve mitochondrial function and phenotype, although these treatments lost effectiveness at late time points.

#### Model prediction

**Figure 7:**
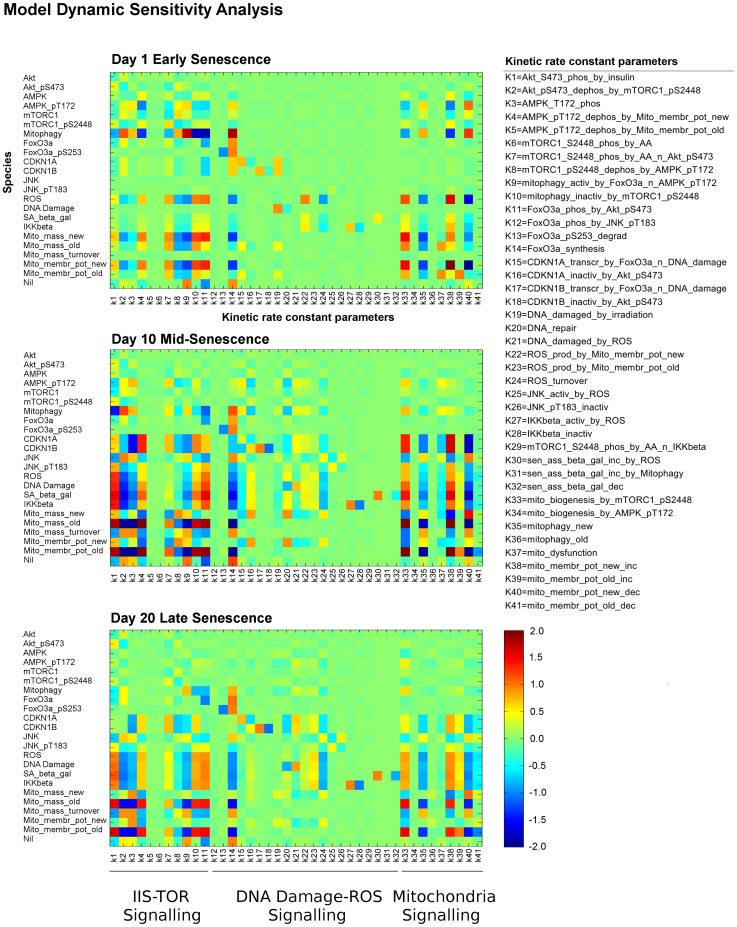
Dynamic sensitivity analysis. Sensitivity analysis at 1, 10 and 20 days post-irradiation indicated three states of cellular senescence: early, middle and late senescence. Sensitivity analysis was computed for the model species (y axis) upon perturbation of the kinetic rate constant parameters (x axis). The parameters k1–k11 are involved in the IIS-TOR signalling sub-network, whereas the groups of parameters k12–k32 and k33–k41 regulate the DDR-ROS signalling response and mitochondrial dynamics, respectively. Model species sensitivities changed over time, highlighting a dysfunction for mitophagy and mitochondria, and DDR-ROS stress-response at the later time points. These dysregulations consolidated over time and ultimately attenuated the global sensitivity in the model. Scale bar represents the normalised positive or negative sensitivity of each species to each rate constant.

By splitting the total mitochondrial population into new functional and old dysfunctional mitochondria in the model, we predicted that the treatments mainly acted on the former mitochondrial sub-population. This suggested that the applied inhibitions could delay the senescence process but not reverse it, as eventually the mitochondrial population became dysfunctional. To investigate this hypothesis, we computed a dynamical sensitivity analysis for the model, to study the dynamics of the relationships between parameters in the whole model. To do so, we computed the sensitivities of the model species upon perturbing the model kinetic rate constant parameters at days 1, 10 and 20 post-irradiation (Figure 7). This aimed to identify which of the species/modules within the network were still responsive at later time points by investigating changes in sensitivity over time.

At day 1 post-irradiation (Figure 7, early senescence panel), the IIS-TOR signalling pathway species, and in particular mitophagy, showed effective sensitivity to both its internal kinetic rate constants and mitochondrial dynamics. The DDR-ROS signalling response was still low-moderate and the predominant signals derived from mitochondria were still driven by the new population. Mitochondrial species within the model were sensitive to the IIS-TOR and mitochondrial kinetic rate constants. Interestingly, the mitochondrial dynamics showed that mitochondrial biogenesis by mTORC1 (parameter k33) also promoted mitochondrial dysfunction (species Mito-mass-old). Mitochondrial kinetic rate constants affected ROS production and IIS-TOR-AMPK pathways by affecting cellular energy levels (species AMPK-pT172). Cell cycle arrest programs, shown by the species CDKN1A and CDKN1B, were already started at day 1, being mostly dependent on irradiation-induced DNA damage (parameter k19).

At day 10 post-irradiation (Figure 7, mid-senescence panel), the DDR-ROS stress responses and mitochondrial dysfunction were highly active. The DDR-ROS stress response species were affected by almost all the model kinetic rate constants indicating that the overall network was sustaining a stress signalling response. In particular, mitochondrial kinetics predominantly fed a stress response by increasing ROS production and strengthening cell cycle arrest, whose control was now driven by mitochondrial and IIS-TOR signalling rather than by DDR-ROS rate constants. At this stage, mitochondrial dysfunction (species Mito-mass-old) was highly sensitive to the IIS-TOR signalling pathway dynamics, positively by anabolic signals, such as Akt-mTORC1 (parameters k1 and k7), and negatively by catabolic signals, such as AMPK-FoxO3a (parameters k3 and k14) and mitophagy (parameter k9). Interestingly, the overall sensitivity for the species mitophagy was largely reduced, indicating a gradual loss in feedback response from IIS-TOR and mitochondria. Conversely, mitophagy was found to become increasingly regulated by DDR-ROS-dependent kinetic rate constants. This gradual loss in sensitivity was also found for the species representing the IIS-TOR signalling pathway. These intermediate time-point results showed that the network was highly dysregulated by severe mitochondrial dysfunction, sustained DDR-ROS stress responses, and moderate insensitivity of the IIS-TOR-mitophagy signalling pathway.

At day 20 post-irradiation (Figure 7, late senescence panel), the network globally showed a decrease in sensitivity as compared to the mid-senescence case. The species involved in the IIS-TOR signalling pathway were only marginally affected by the overall network dynamics, and in particular mitophagy was largely insensitive. The DDR-ROS stress response and mitochondrial dysfunction sensitivities were still sustained, although not as markedly as before. Cell cycle arrest was preserved and maintained by the kinetics of IIS-TOR, DDR-ROS and mitochondria. In Figure 1E, both the *in silico* and *in vitro* time courses suggested that the network was approaching stabilisation after day 15. The sensitivity analysis described here validates this observation of the stabilisation to a senescent phenotype. To formally test the stability of this cellular senescence state, we calculated the Lyapunov exponents of the model (which allowed us to determine the network stability over time) and concluded that the model was asymptotically Lyapunov stable, since the 19 computed Lyapunov exponents were all negative (Table 1). This indicated that the variable levels eventually reach equilibrium. Although this state of cellular senescence, that we term late senescence, was dynamically stable, this did not mean that its variance was low. In fact, the global decrease in sensitivity upon kinetic rate constants indicated that the semantics of these model parameters, e.g. promoter or inhibitor, became more uncertain. As a consequence, this uncertainty increased system noise and decreased network robustness, which, in the context of a cell, translated into weak signalling regulations and therefore poor intervention effectiveness. We tested this prediction by simulating the model stochastically and observed an increase in variance especially for the oxidative stress/DNA damage signalling and to a lesser extent for the Mitophagy and mitochondrial mass species (Figure S24). In agreement with these predictions, our experimental data (Figures 1E, 2B, 3B and 4B) indicated an increase in variance at late time points and a loss in intervention effectiveness for ROS, mTOR or ROS-mTOR interventions at day 21. In particular, we attributed this gradual loss in intervention effectiveness to 1) gradual loss of mTOR-Akt sensitivity upon all the kinetic rate constants in the network, and 2) persistent dysfunction in mitophagy and mitochondria, ultimately largely unaffected by ROS inhibition.

**Table 1:** Model Lyapunov exponents.

Lyapunov Exponents	
λ_1_	−0.0136218
λ_2_	−0.0052144
λ_3_	−0.0748144
λ_4_	−0.0660512
λ_5_	−0.154906
λ_6_	−0.175105
λ_7_	−0.352563
λ_8_	−0.403684
λ_9_	−0.44979
λ_10_	−0.947157
λ_11_	−1.10172
λ_12_	−1.96465
λ_13_	−3.41447
λ_14_	−5.19749
λ_15_	−34.4987
λ_16_	−57.6887
λ_17_	−1094.69
λ_18_	−2694.43
λ_19_	−5721.87
**Sum**	**−9617.50**
**Avg divergence**	**−9616.65**

The Lyapunov exponents computed for this model were all negative, indicating that the model was asymptotically Lyapunov stable and therefore the trajectories eventually converged to an equilibrium.

This network-wide exploration of the model dynamic sensitivity explained why our interventions were only effective until late senescence and then ultimately lost effectiveness. Hence, the investigation of this cellular state of senescence showed intrinsic difficulties in applying intervention to delay or reverse this state to normal due to multiple severe dysfunctions within the network concomitant with a global loss of network sensitivity.

## Discussion

The medium to long term effect of stress-induced senescence has previously been investigated with a focus on DNA damage-activated p53 and cell cycle arrest [52], but not in any detail with regard to other players implicated in cellular senescence. In this work, we used a systems biology approach to build the first detailed mathematical model to address cellular senescence. We performed a temporal analysis of the changes that occur in stress activated signalling, bioenergetic and cell cycle signalling, and their effects upon one another over a three week time course of irradiation-induced senescence. The data showed a complex phenotype of changing behaviour over time for many parameters. Of particular interest were the changes in mitochondrial mass, activity and turnover, which directly affect energy production and cause further stress and damage via ROS production. We observed large changes in both mitochondrial mass and membrane potential, neither of which were rescued by the modest increase in mitophagy. Due to the complexity of these interacting pathways, a model was designed to represent the dynamical process of irradiation-induced cellular senescence. Although inevitably somewhat abstracted, this model fitted our in vitro data, suggesting that the network representation was a close approximation to the biological system that we wanted to investigate. Importantly, such a system enabled us to investigate strategies of drug interventions that might reduce senescence progression in the medium to long term. *In silico* predictions indicated that mitochondrial ψm and mass could be restored by scavenging ROS or inhibiting mTOR. However, the model also indicated loss of effectiveness of interventions at late time points. A combination of the two treatments was shown to out-perform the previous results, and to reduce DNA damage. *In vitro* tests confirmed these predictions and also highlighted an increase in variance at late time points. Finally, these simulated interventions were further supported by predicting the network behaviour upon over-activation of AMPK, FoxO3a or mitophagy, indicating the central role of energy and turnover in inducing senescence.

Whilst the model does not specifically include fusion and fission, the detailed modelling of the shift between freshly generated and aged mitochondria allowed us to infer differences in fission rates in senescence. Fusion and fission are tightly coupled, required for mitophagy of mitochondria [53], essential for removal of damaged mitochondria with reduced ψm and may have deep evolutionary origins connected with ageing [54]. Our measurements of fusion-fission show a decrease in both processes in senescence, whilst maintaining a tight coupling between them in terms of their frequencies. Lower fission therefore explains the lack of mitophagy increase, even though there is apparently a large increase in the necessary proteins. A recent investigation into fusion and fission in neurodegenerative disease models identified similar findings, with reduced rates of fusion and fission [55]. In this study, the authors also showed that mitochondrial motility was essential for fusion. Whilst not quantified, we observed a similar decrease in motility in our senescence model, highlighting the overlap between neuronal disease and cellular ageing. It is therefore possible that the decreased fusion-fission in senescence is due to decreased mitochondrial motility, a factor which is known to induce mitochondrial diseases in some cases of familial Parkinson's disease [56]. Intracellular motility was not encapsulated in our model, and therefore would be important to address in future models investigating mitochondrial dysfunction.

Over the induced senescence time course, why were the treatments effective only up until a late-senescence state? Which were the signalling pathways involved in this dysfunction? What was the nature of this cellular senescence late state? What was its role? Could the dysregulation be reversed? To answer these questions, we computed a dynamic sensitivity analysis and detected a gradual loss in network sensitivity and, in particular, a deficiency in the IIS-TOR signalling pathway and mitophagy regulation at late time points. Although we proved the asymptotic stability of this cellular senescence state, the overall poor sensitivity in the network also predicted two negative consequences: the detected loss in treatment efficacy and increase in network variance. The former indicated that the further the system went into late-senescence, the more combinatorial treatments were required to alleviate the phenotype due to the multiple cellular dysfunctions that were apparent at later time points. The latter implied that the network was less capable of responding to signalling programs due to high variance within the system. In this prediction, a cell in the late-senescence state would not only be intrinsically dysfunctional, but also more susceptible to external insults, due to loss of effectiveness in the response mechanisms.

Aside from late senescence, the model also predicted the mechanism underlying the transition from normal to early senescence. Interestingly, it indicated that multiple factors, such as DNA damage, increased ROS production and gradual mitochondrial dysfunction, are all likely to be playing a role in this transition. These players would mutually re-enforce their activation in the signalling network. In the specific case of irradiation-induced senescence, this synergistic transition would start from DNA damage production and loss of mitochondrial function. Subsequently, in early-mid senescence the cellular decision appears to reach a crossroad, with anabolic signalling driving further damage and catabolic signalling tending to suppress it. All of these events lead eventually to a persistent cell cycle arrest and an initial drive of mTOR-dependent mitochondrial biogenesis and autophagy [57] in order to replace damaged mitochondria and restore cellular energy levels. This over-activation of mitochondria in combination with increased cellular stress activation promotes the formation of dysfunctional mitochondria, over-riding mitochondrial turnover by mitophagy, and accumulating cellular damage. Hence, although mTOR represents an essential player in this reinforcement loop, our data suggest that other drivers, such as DDR and ROS, are also required in irradiation-induced senescence.

Despite its power, the current model misses some components that are likely to be important. Most significant of these is the complexity of the inflammatory system as controlled by NF-κB, TNF-α and TGF-β signalling pathways. This inflammatory response is heavily abstracted in this model by JNK through ROS regulation. The full inflammatory system has important positive-feedback loops that may develop independently and may therefore be responsible for permanent activation of JNK, and contribute to ROS signalling stabilisation (Jurk *et al.*, Nature Comm., in press). Control of pro-inflammatory cytokine production and activation of other members of the senescence associated secretory phenotype would obviously impinge upon the pathway to cellular senescence [58]–[60]. If added to a future version of this model, these would allow a detailed investigation of the development of senescence within a multi-cellular environment where paracrine signals are operative. Finally, the mitochondrial dynamics of fusion and fission were also abstracted, due to the difficulty of representing their regulation in the network. Their role in mitochondrial homeostasis would warrant inclusion in future work. Nevertheless, the abstraction applied in this study was sufficient for the conclusions provided, and represents, we believe, a worthwhile advance.

In conclusion, the nature of the biological pathways driving cellular senescence presents a challenge to gaining a sufficient understanding in order to identify novel targeted therapies against the many age-related diseases. A considerable contribution in representing this highly networked biological system and eliciting its properties is offered by mathematical modelling.

Using an integrative systems-biology approach, we determined that multiple interventions for limiting the progression of senescence, and therefore the impact on age-related diseases, are possible: these include down-regulation of ROS and mTOR. Due to the increase in cellular dysfunction over time, these interventions should be applied at early stages and possibly integrated with other interventions aimed at regenerating mitochondria and mitophagy, and reducing inflammation.

## Materials and Methods

### Mathematical model

The Ordinary Differential Equation (ODE) mathematical model consists of 23 variables and 41 mass action reactions covering 5 cellular modules: DNA-damage, oxidative stress, FoxO, IIS-mTOR and mitochondria. There are 3 inputs: insulin, amino acids and irradiation. The first two inputs were set as constants, whereas the irradiation input was an impulse at time 0 for 5 min, consistent with the *in vitro* cell treatment. The cells were not starved by insulin or amino acids pre-irradiation to avoid cell death upon irradiation, and this was modelled by assuming an equal protein amount fixed at the basal level for both the phosphorylated and dephosphorylated states across the IIS-TOR signalling pathway. In pre-irradiated cells, the stress response induced by DNA-damage and ROS was low but still present and all the variables directly involved in these signalling pathways (DNA-damage, ROS, JNK, SA-β-gal, IKK-β) were set to reflect this initial basal level. In the model, the mitochondrial population was split into discrete groups: new and old. For the initial state, amounts for the new group (Mitochondrial-Mass-new, Mitochondrial-Membrane-Potential-new) were set to basal levels, whereas amounts for the old group (Mitochondrial-Mass-old, Mitochondrial-Membrane-Potential-old) were assumed negligible as in healthy cells, dysfunctional mitochondria are usually removed by mitophagy. The model was linked to the *in vitro* data by 14 observables covering the entire network. Where the observable provided a composite measure of states, such as nuclear/cytoplasmic FoxO3a or the sub-populations of mitochondria, the respective observable was linked to the sum of these states. Hence, the observable FoxO3a-total was associated to the sum of nuclear and cytoplasmic FoxO3a species, the observable Mitochondrial-Mass was associated to the sum of Mitochondrial-Mass-new and –old and the observable Mitochondrial-Membrane-Potential was linked to the sum of Mitochondrial-Membrane-Potential-new and –old. A legend of all the names (e.g. species, parameters, observables, etc.) used in the model is provided in Table S2 and a complete list of model ODEs is provided in Table S3.

### Parameter estimation and identifiability

The Matlab Toolbox PottersWheel 3.0.12 [61] and the PottersWheel identifiability toolbox MOTA [62] were used for estimating the kinetic rate constants in 7 rounds of parameter estimation and identifiability (Table S4). All parameters were fitted within the interval [1e−06, 1e+04]. The kinetic rate constants determining the activation/inactivation for IKK-β were assumed *a priori* due to lack of clear experimental data. IKK-β is not an essential component of this model as it only represents an intermediate step between ROS and mTORC1, but we opted to include it as we wanted to make an explicit connection between oxidative stress-inflammation [59] and inflammatory-dependent mTORC1 activation [63]. The remaining 39 parameters were calibrated using the optimisation algorithm TrustRegion (MaxIter: 150; TolFun: 1e−06; TolX: 1e−06) and Matlab integration algorithm ode15s (AbsTol: 1e−06; RelTol: 1e−04; MaxNumSteps: 1500). For each round, 40 independent sequences of 500 fits each were performed and combined to generate a total of 20,000 fits, which were used for analysis. All the sequences were always computed using the best current fit as the initial assignment and randomising it with disturbance strength of 0.4. This strategy allowed us to explore the parameter space extensively and additionally, to limit the number of non-convergent solutions. To calculate the sequences, a cluster of 6 GNU/Linux computers with a total of 60 cores was employed using the open source job scheduler Openlava (http://www.openlava.org/). As the standard deviation of the experimental time points was often large, a 10% error model of observation was adopted for the task of parameter estimation. This improved the approximation of the model to the data. For each round, nonlinear MOTA identifiability analysis was used for identifying tuples of related parameters by selecting the best 30% fits from the total 20,000 fits. A parameter was considered non-identifiable when the correlation coefficient (CC) and the coefficient of variance (CV) for the tuple of its related parameters were higher than 25% and 0.9, respectively. The MOTA identifiability matrices and the respective correlation plots, as calculated for each round, are reported in Figure S4, S5, S6, S7, S8, S9, S10, S11, S12, S13, S14, S15, S16, S17. To assess the contribution to parameter estimation variability a principal component analysis was performed at each round. Interestingly, this analysis highlighted that at least half of the parameters estimated at each round did not affect the model variance (Figure S18).

### Modelling tasks

Copasi 4.8.35 [64] was used for simulations with perturbation of model species. Simulations were run for protein inhibition at 10 levels from 0% (control) to 90% for mTORC1 (simulating Rapamycin treatment), Akt-mTORC1 (simulating Torin treatment) and ROS. Simulations were run for protein over-activation at 10 levels from 0% (control) to 150% for AMPK, FoxO3a and Mitophagy. For the species in which the sum of the two internal activation/inactivation states was constant (e.g. mTORC1, Akt-mTORC1 and AMPK), the protein amount was simply perturbed at the beginning of the simulation. For species whose level varies over the time-course (e.g. ROS, FoxO3a and Mitophagy), their protein amount was constantly modified using a dummy species introduced for each of the above species. Hence, these dummy species simulated either an activator or an inhibitor. The kinetic rate for this external perturbation was fixed, but, the amount for these dummy species was determined such that the average activation level for the perturbed protein species over the time course was 10% (if inhibited) or 250% (over-activation) with respect to the average activation level for the corresponding unperturbed protein species (Figure S19). Deterministic simulations were performed in Copasi using the deterministic algorithm LSODA, configured with the following parameters: duration, 21; interval size, 0.02; intervals, 1050; integrate reduced model, 0; relative tolerance, 1×10^−6^; absolute tolerance, 1×10^−12^; maximum internal steps, 10,000. Stochastic simulations were performed in Copasi using the Direct Method (Gillespie), configured with the following parameters: duration, 21; interval size, 0.01; intervals, 2100; maximum internal steps, 100,000; use random seed, 0; random seed, 1. Model 2D sensitivity analysis for model species was computed at days 1, 10 and 20 by perturbing the kinetic rate constant values. Double perturbation data were computed using Copasi and Matlab by inhibiting or over-activating the two parameters from 0% to 90% (inhibition) or 0% to 150% (over-activation) by steps of 0.15% and 0.25% respectively. Lyapunov exponents for the reduced system were computed in Copasi using the Wolf Method, configured with the following parameters: number of exponents, 19; start averaging after t, 21; orthonormalisation interval, 0.0001; overall time, 50; relative tolerance, 1×10^−6^; absolute tolerance, 1×10^−10^; maximum internal steps, 10,000. The divergence was calculated as the average over the trace of the Jacobian as implemented in Copasi. Model structure was graphically represented using CellDesigner [65], [66] and exported to SBML [67] Level 2 Version 4 using Potterswheel (Model S1).

### Statistics

The programming language R (http://cran.r-project.org/) was used for computing time course mean and standard deviation of 5 independent *in vitro* time-course measurements. Mean and standard deviation values were then used for calibrating the model observables. The goodness-of-fit statistical measures χ^2^ [61] and AIC [68] were used to assess the quality of fit of the model at each calibration round. All these measures were directly computed using the PottersWheel toolbox. R was selected for the graphic representation of the identifiability matrix computed with MOTA and single perturbation plots. For establishing the model parameterisation, *in vitro* experiments were performed with between 3 and 5 independent replicates. Intervention experiments were performed in triplicate, except for SA-β-gal staining, which was performed in duplicate. For data that were not normally distributed, group means were compared using a Mann–Whitney rank sum test. Multiple comparisons were performed by ANOVA followed by Dunn's test for comparison of individual subgroups. All analyses were performed in Sigmaplot or R unless stated otherwise. Data were tested for normality using Shapiro-Wilk normality test. Significance is denoted on all graphs with * p<0.05.

### Antibodies

The full list of antibodies with their respective catalogue numbers and the dilutions used for the respective staining (western or immunofluorescence) is provided in Table S5.

### Cell culture

MRC-5 human embryonic lung fibroblasts (obtained from ECACC) were grown in Dulbecco's Modified Eagle's Medium plus 10% fetal calf serum (BioSera, Ringmer, UK), 2 mM l-glutamine, 1× penicillin/streptomycin at ambient oxygen partial pressure, 5% CO_2_, 37°C in a humidified incubator (Binder Instruments). All experiments utilised cells which were growing in the logarithmic part of their growth curves. For irradiation, cells were plated to give approximately 80% confluency just prior to irradiation. 20 Gy X-irradiation was given to the cells using an X-rad225 irradiator (Precision x-ray Inc., N.Branford, CT USA) to induce senescence. Post irradiation, cells were immediately supplied with fresh medium, and then fed three times a week over the timecourse (with or without drugs/DMSO each time). 0 day timepoints were unirradiated and harvested 2 hours after treating with their respective drugs if necessary. Antioxidant treatment was performed with the addition of 100 IU SOD and 100 IU catalase to the medium as described previously [12]. mTOR inhibition was performed using Torin 1 (Tocris Bioscience, Bristol) at 10 nM. Bafilomycin A treatment was performed for 1 hour at 400 nM.

### Western analysis

2×10^5^ cells were plated in 60 mm petri dishes (Corning) 1 day prior to irradiation. Medium was removed and cells were washed once with PBS before harvesting by scraping directly in SDS loading buffer, transferred to screw cap microcentrifuge tubes, boiled for 5 minutes, then stored at −20°C until required. Samples were run on 10% SDS-PAGE gels (Biorad) before transferring to PVDF (Biorad). Membrane washes and antibody dilutions were performed using Tris-buffered saline with 0.1% (v/v) Tween20 (TBST). All primary antibody incubations were performed in TBST with 5% BSA, overnight at 4°C before washing, applying HRP-conjugated secondary antibody (NEB) for 1 h at room temperature and subsequently detecting HRP activity using Supersignal West Pico substrate (ThermoScientific, Cramlington, UK) with a LAS4000 imager (Fujifilm, Japan).

### Immunofluorescence

2.5×10^4^ cells were plated on 16 mm Ø glass coverslips (#1.5) in 12 well plates (Corning) 1 day prior to irradiation. Medium was removed and wells were washed once with PBS before adding 250 µl of pre-warmed 4% paraformaldehyde (PFA) and incubating at 37°C for 2 minutes. PFA was removed and wells were washed 3 times with PBS. Cells were stored in 2 ml of PBS at 4°C until required. For immunostaining, TBS was used for all washes, and antibody incubations were performed using TBS plus 0.3% (v/v) Triton X-100. All primary antibody incubations were performed overnight at 4°C, with secondary detection using AlexaFluor conjugated antibodies (Life technologies, Paisley, UK) for 1 h at room temperature. Coverslips were mounted using Prolong Gold with DAPI (Life technologies). Imaging was performed using a spinning disk confocal head (CSU-X1, Yokogawa, Japan) mounted on an Axiovert 200M equipped with a 63× NA1.4 objective driven by Axiovision software (v4.8.1, Zeiss, Cambridge, UK). Each position was captured as a z stack and deconvolved using Huygens (v4.2, SVI, Netherlands). Fluorescent intensities and γH2A.X foci counts were calculated using ImageJ (v1.45j, http://rsb.info.nih.gov/ij/). Number of colocalised objects in LC3- COXIV stained cells were determined using Huygens Colocalisation analyser.

### Live cell microscopy

Mitochondrial mass and ψm were determined by fluorescence microscopy using TMRM and Mitotracker Green as described previously (Passos et al., 2010), analysing at least 100 cells per treatment and time point. For following mitochondrial dynamics, cells were transduced with baculovirus encoding mitochondrially targeted red fluorescent protein following the manufacturer's protocol (Invitrogen). Single cell images were captured using a Zeiss CellObsever spinning disk confocal equipped with a heated, humidified stage (95% air, 5% CO_2_) using a 100× 1.4NA objective (Zeiss) as a z stack encompassing the entire cell every 20 s for 30 minutes. Images were then deconvolved with Huygens and objects identified using the same parameters between cells with Huygens Object Analyzer. For each cell, an 18.2 µm^2^ area was then cropped out and the total mitochondrial volume of the object identified time series were manually analysed over time for numbers of fusion and fission events per time point (every 20 s). Huygens was used to determine the total mass at each time point in the cropped regions. Total fusion and total fission events per minute were then summed every minute over the time course. To measure mitophagy, cells were transduced with baculovirus encoding mitochondrially targeted red fluorescent protein and lysosomally targeted green fluorescent protein following the manufacturer's protocol (Invitrogen). Images were acquired and processed as described for LC3-COXIV stained cells.

### Flow cytometry

Flow cytometric measurements of mitochondrial mass and superoxide measurements (using mitotracker green and DHE respectively) were performed as described previously (Passos et al., 2007), analysing 30,000 cells per treatment and time point.

## Supporting Information

Figure S1 **Measures of mitophagy during stress induced senescence.** (A) Cells were fixed at the indicated timepoint with or without 1 hour pre treatment with 400 nM Bafilomycin A, and then stained for LC3 and COX-IV. Number of co-localised objects were determined as described in the Methods. Replicatively senescent MRC5 cells were included as a positive control. (B) Live cell microscopy was performed using co-transduction of GFP and RFP targeted lysosome and mitochdondrial baculovirus constructs respectively at the timepoints shown. Bafilomycin A treatment and analysis were performed as described for LC3-COX-IV stained cells.
(TIF)

Figure S2 **Mitochondrial mass data as determined by flow cytometry.** Cells loaded with Mitotracker Green were analysed by flow cytometry at the time points indicated post 20 Gy irradiation. Data represent the median fluorescence intensity from 30,000 cells per time point relative to the 0 day control.
(TIF)

Figure S3 **Cell viability.** Cell viability was determined from trypsinised cells (plus spent medium) at the indicated time points post 20 Gy irradiation by counting live/dead cells using trypan blue and a haemocytometer. A minimum of 120 cells were recorded per dish per time point, 3 dishes per time point. Data are mean +/− SD. No significant changes in viability were seen over time.
(TIF)

Figure S4 **MOTA identifiability matrix for round 1 of parameter estimation.** In this round the kinetic rate constants k3, k4, k17, k18, k19, k20, and k37 were fixed since they were identifiable using MOTA identifiability analysis. *: Correlation Coefficient (CC)>0.9 and Coefficient of Variation (CV)>0.25; **: Correlation Coefficient (CC)>0.9, Coefficient of Variation (CV)>0.25 and number of tuples showing this correlation (#)>1. Format: ParameterCode <CC CV # (“Tuple of related parameters”)>.
(TIF)

Figure S5 **Correlation plots for round 1 of parameter estimation, as detected by MOTA identifiability analysis.** Plots for the tuples of related parameters reported in the MOTA identifiability matrix in Figure S4.
(TIF)

Figure S6 **MOTA identifiability matrix for round 2 of parameter estimation.** In this round the kinetic rate constants k1, k2, k15, k16, k21, k30, k35, and k36 were fixed since they were identifiable using MOTA identifiability analysis.
(TIF)

Figure S7 **Correlation plots for round 2 of parameter estimation, as detected by MOTA identifiability analysis.** Plots for the tuples of related parameters reported in the MOTA identifiability matrix in Figure S6.
(TIF)

Figure S8 **MOTA identifiability matrix for round 3 of parameter estimation.** In this round the kinetic rate constants k25, k26, and k32 were fixed since they were identifiable using MOTA identifiability analysis. The parameters k8, k9, and k12 were locked as reported in Table S4. The parameters k8 and k9 were part of two couples of related parameters. The former k8 with k7, the latter k9 with k10. All these two couples only related internally with themselves and therefore formed two locally defined correlations. The parameter k12 only related with k11, although k11 was not dependent on k12. Hence, this also formed a locally defined correlation.
(TIF)

Figure S9 **Correlation plots for round 3 of parameter estimation, as detected by MOTA identifiability analysis.** Plots for the tuples of related parameters reported in the MOTA identifiability matrix in Figure S8.
(TIF)

Figure S10 **MOTA identifiability matrix for round 4 of parameter estimation.** In this round the kinetic rate constants k7, k10, k11, k13, k14, and k33 were fixed since they were identifiable using MOTA identifiability analysis.
(TIF)

Figure S11 **Correlation plots for round 4 of parameter estimation, as detected by MOTA identifiability analysis.** Plots for the tuples of related parameters reported in the MOTA identifiability matrix in Figure S10.
(TIF)

Figure S12 **MOTA identifiability matrix for round 5 of parameter estimation.** In this round the kinetic rate constants k34, k38, k40, and k41 were fixed since they were identifiable using MOTA identifiability analysis.
(TIF)

Figure S13 **Correlation plots for round 5 of parameter estimation, as detected by MOTA identifiability analysis.** Plots for the tuples of related parameters reported in the MOTA identifiability matrix in Figure S12.
(TIF)

Figure S14 **MOTA identifiability matrix for round 6 of parameter estimation.** In this round the kinetic rate constants k22, k23, k24, and k39 were fixed since they were identifiable using MOTA identifiability analysis.
(TIF)

Figure S15 **Correlation plots for round 6 of parameter estimation, as detected by MOTA identifiability analysis.** Plots for the tuples of related parameters reported in the MOTA identifiability matrix in Figure S14.
(TIF)

Figure S16 **MOTA identifiability matrix for round 7 of parameter estimation.** In this round the kinetic rate constants k5, k6, k29, and k31 were fixed since they were identifiable using MOTA identifiability analysis.
(TIF)

Figure S17 **Correlation plots for round 7 of parameter estimation, as detected by MOTA identifiability analysis.** Plots for the tuples of related parameters reported in the MOTA identifiability matrix in Figure S16.
(TIF)

Figure S18 **Principal component analysis for model at each round of parameter estimation.** To further investigate the source of variability, principal component analysis (PCA) of the model was computed at each round of parameter estimation. Interestingly, this analysis indicated that only about half of the estimated parameters did not significantly contribute to the overall variance for each round. This suggested that a group of parameters could have been potentially identified at each round.
(TIF)

Figure S19 **Simulated tools for model inhibition or over-activation over time.** (A) To inhibit ROS over time, a simulated ROS inhibitor species was added to the model. This new species reduced ROS levels and acted as an *in vitro* ROS scavenger. The abundance for this species was estimated in order to achieve ROS inhibition to 10% (blue) as compared to the control (white) throughout the time course. (B) In analogy, two new species were created for over-activating mitophagy and FoxO3a, respectively. The abundance for these two species were estimated to achieve a mitophagy or FoxO3a over-activation of 150% (magenta) as compared to the corresponding control (white) throughout the time course. Each boxplot represents the median and two quartiles, whereas the bars indicate the minimum and the maximum values, as estimated from day 1 to day 21.
(TIF)

Figure S20 **Inhibition of ROS and mTOR *in vitro*.** Torin and ROS inhibition efficacy. (A) Cells were irradiated with 20 Gy X irradiation and then treated with Torin1 or DMSO as described in Methods. Lysates were probed with total mTORC1 and mTORC1-S2448 antibodies. Band intensities were quantified relative to Tubulin loading control and plotted as mean +/− SD ratios of mTORC1-S2448 to total mTORC1 (3 repeats). (B) Cells were irradiated with 20 Gy X irradiation and then treated with SOD and catalase as described in Methods. Cells were stained with DHE to measure intracellular superoxide levels by flow cytometry. Time course data over 21 days are plotted (n = 3).
(TIF)

Figure S21 **Analysis of the two mitochondrial sub-populations upon ROS-mTOR combined intervention.** The internal states for the new and old mitochondrial sub-populations were also investigated upon combined ROS-mTOR simulated intervention. Regarding the mitochondrial membrane potential, the global effect of these interventions mainly resulted from changes in the sub-population of new mitochondria, which showed a strong synergistic response at particular levels of mTOR- ROS inhibition. The effect upon old mitochondrial øm was almost entirely dependent upon mTOR inhibition, but still changed their potential by an insignificant amount compared to the new mitochondrial population. Concerning the mitochondrial mass, the perturbation of combined ROS-mTOR acted predominantly on the young population, although these changes were largely hidden in the overall population (Figure 4A) due to the larger proportion being comprised of the old population. The point (0, 0) indicates the control (no inhibition).
(TIF)

Figure S22 **Analysis of senescence-associated β-galactosidase staining upon upon ROS-mTOR combined intervention.** (A) Cells were fixed at the indicated timepoints and assayed for senescence–associated β-galactosidase followed by counterstaining with nuclear fast red prior to imaging. An average of ∼300 cells were counted per coverslip, with any blue cytoplasmic staining being considered positive. Data are n = 2 ± SD. Combined intervention produced significantly lower positive cells at 3 and 6 days post irradiation relative to DMSO control (P<0.05). (B) Example images of stained coverslips showing the decreased intensity of staining observed in treated cells.
(TIF)

Figure S23 **Additional readouts upon AMPK, FoxO3a or mitophagy simulated over-activation.** The model predicted a decrease in the DNA damage/oxidative stress response pathways upon over-activation of AMPK, FoxO3a or mitophagy. AMPK was predicted to achieve the strongest inhibition throughout the time course as compared to FoxO3a or mitophagy. Interestingly, an over-activation of FoxO3a was predicted to initially increase and then reduce CDKN1A levels as compared to the control (black line), suggesting differential regulation of cell cycle arrest over time.
(TIF)

Figure S24 **Model stochastic simulation showed increase stochasticity over time.** Model stochastic simulations up to 20 days graphically showed increased stochasticity for the oxidative stress/DNA damage signalling species. Moderate increased variance was also detected for the species Mitophagy and the species representing mitochondrial mass and membrane potential. Number of stochastic runs: 500; black line indicates the means, dark grey area indicates 95% confidence interval of the mean and grey area indicates a standard deviation. (A) Species associated to *in vitro* data (observable variables). (B) Mitochondrial internal states (derived variables).
(TIF)

File S1 **File collecting all the supporting tables and figures.** All the supporting tables and figures with their corresponding legends are combined into one document in this file.
(PDF)

Model S1 **SBML code for the model.**
(XML)

Movie S1 **Mitochondrial fusion and fission in young and senescent fibroblasts over 30 minutes time courses.**
(MP4)

Table S1 ***In vitro* data set used for estimating the model parameters.** For each model observable, the mean and standard deviation (reported as StdCol) of the quantified *in vitro* intensities are reported. The table is formatted for Potterswheel v. 3.0.12.
(XLS)

Table S2 **Legend of the model variables.** This table defines the unique codes for the model kinetic rate constants (K_i_), species (X_i_), scaling factors (S_i_), observables (Y_i_) and constraints (CS_i_). The values for the non-estimated parameters are also provided. The lambda term in the block ‘Constraints’ defines the constraint strength (higher values means harder constraints) as implemented in PottersWheel.
(TIF)

Table S3 **ODEs table of the model.** The Ordinary Differential Equations (ODEs) for the 23 species defining the model.
(TIF)

Table S4 **Table of the estimated parameters in the model.** Up to 7 rounds of alternated parameter estimation and MOTA identifiability analysis were computed in order to progressively determine all the kinetic rate constant parameters. The internal round columns include the following labels: Assumed, Fixed and Locked. A parameter was termed assumed when it was assumed a priori, and therefore was not estimated. The only assumed parameters were related to the IKK-β dynamics. A parameter was termed fixed when it could be estimated and identified based on MOTA analysis within a confidence of variance lower than 25% or a correlation coefficient lower than 0.9. A parameter *p* was termed locked when it was fixed without being completely identifiable according to MOTA analysis. This was done only when 1) *p* belonged to a tuple of related parameters and each parameter in this tuple only related to the same parameter tuple (e.g. case for k8, k9), or 2) the other parameters in this tuple were not found to significantly relate with *p*, creating a one-way correlation (e.g. case for k12, see Figure S8). Since these correlations were local and completely confined to the tuple parameters, they did not affect the other unrelated parameters. The parameters in the tuple were only linearly affected. MOTA correlation matrices and plots are provided in Figures S4, S5, S6, S7, S8, S9, S10, S11, S12, S13, S14, S15, S16, S17, and computed from the best 30% of 20,000 fits (see Materials and Methods for more details). For each parameter, the final value, mean, standard deviation and coefficient of variance are reported. Interestingly, parameter estimation reported an extremely low value for the parameter k5, suggesting a poor ATP production by dysfunctional mitochondria. Among the three modalities of mTORC1 activation (amino acids only, amino acids + insulin, amino acids + IKK-β; parameters k6, k7 and k29, respectively), it was detected that mTORC1 activation was significantly stronger in the presence of insulin. Finally, parameter estimation also suggested that the increase in SA-β-gal (see k30, k31) was largely dependent on ROS rather than mitophagy.
(TIF)

Table S5 **List of antibodies.** The antibodies used in this study are shown, with their respective catalogue numbers and the dilutions used for the respective staining (western or immunofluorescence).
(TIF)
