# Supplementary figures and images for: Dynamic Modelling of Pathways to Cellular Senescence Reveals Strategies for Targeted Interventions
Source: PLoS Comput Biol. 2014 Aug 28;10(8):e1003728. doi: 10.1371/journal.pcbi.1003728 (PMC4159174; doi:10.1371/journal.pcbi.1003728)

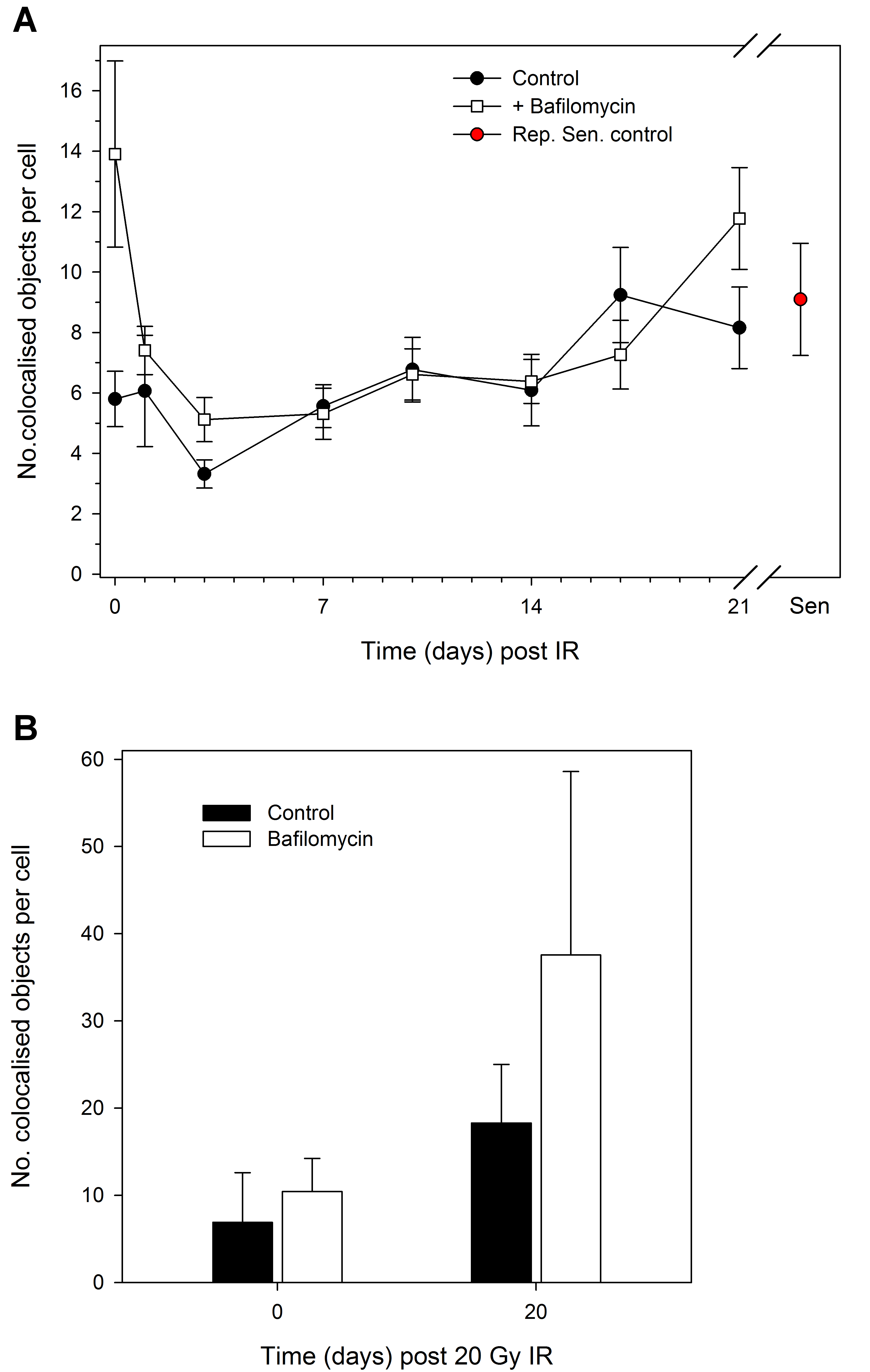

Supplement: Figure S1 — Measures of mitophagy during stress induced senescence. (A) Cells were fixed at the indicated timepoint with or without 1 hour pre treatment with 400 nM Bafilomycin A, and then stained for LC3 and COX-IV. Number of co-localised objects were determined as described in the Methods. Replicatively senescent MRC5 cells were included as a positive control. (B) Live cell microscopy was performed using co-transduction of GFP and RFP targeted lysosome and mitochdondrial baculovirus constructs respectively at the timepoints shown. Bafilomycin A treatment and analysis were performed as described for LC3-COX-IV stained cells. (TIF) [file pcbi.1003728.s001.tif]

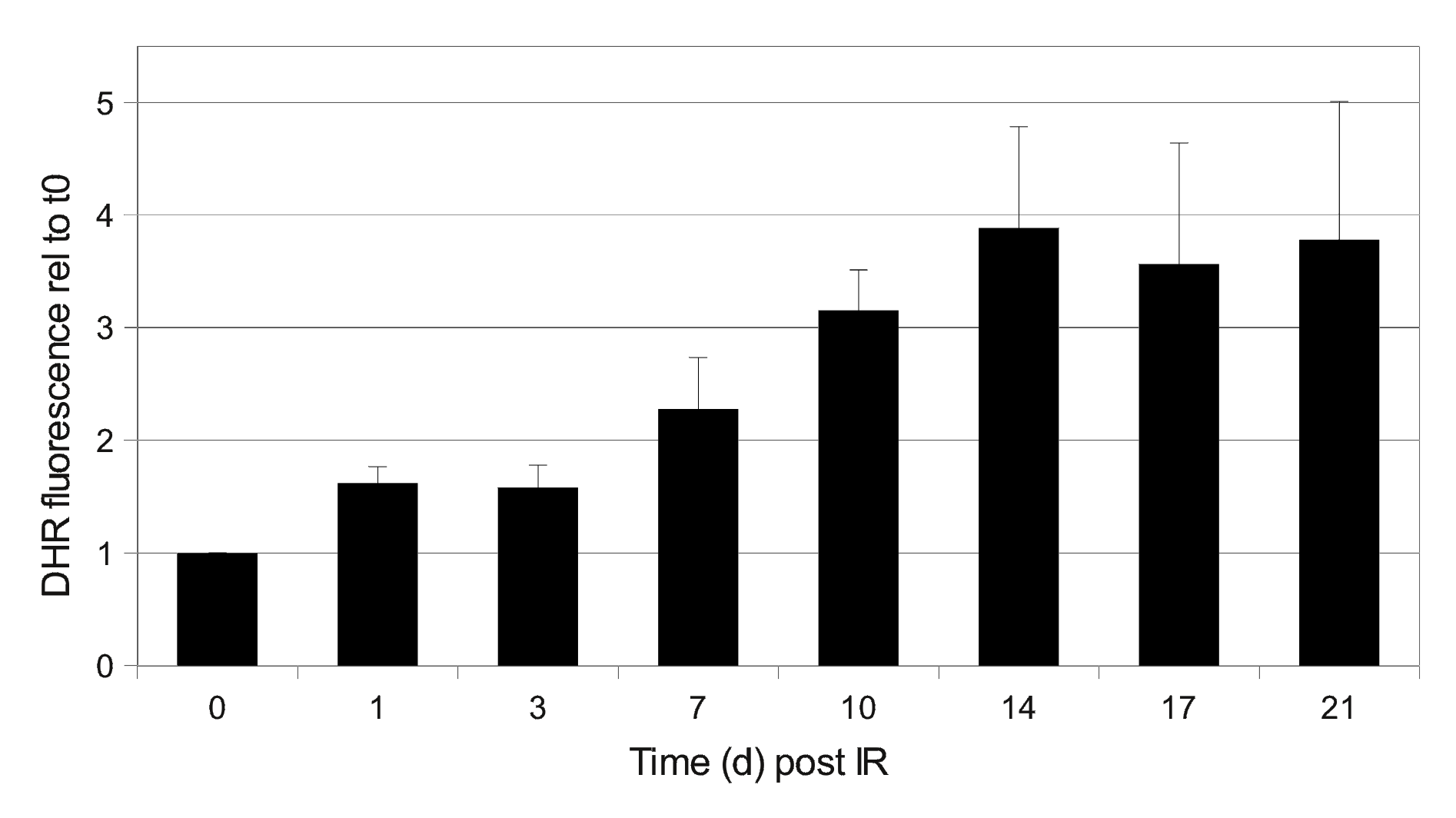

Supplement: Figure S2 — Mitochondrial mass data as determined by flow cytometry. Cells loaded with Mitotracker Green were analysed by flow cytometry at the time points indicated post 20 Gy irradiation. Data represent the median fluorescence intensity from 30,000 cells per time point relative to the 0 day control. (TIF) [file pcbi.1003728.s002.tif]

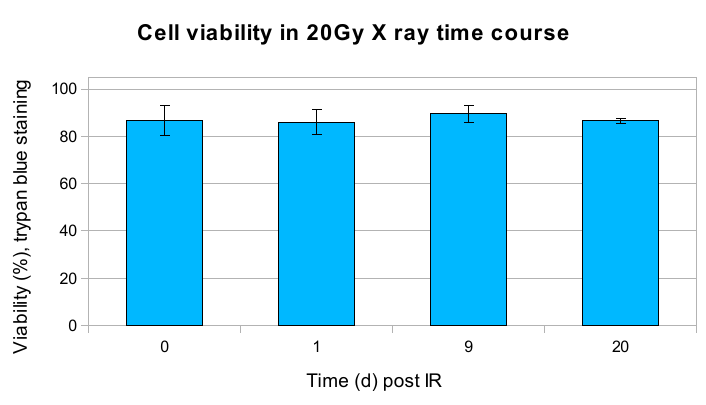

Supplement: Figure S3 — Cell viability. Cell viability was determined from trypsinised cells (plus spent medium) at the indicated time points post 20 Gy irradiation by counting live/dead cells using trypan blue and a haemocytometer. A minimum of 120 cells were recorded per dish per time point, 3 dishes per time point. Data are mean +/− SD. No significant changes in viability were seen over time. (TIF) [file pcbi.1003728.s003.tif]

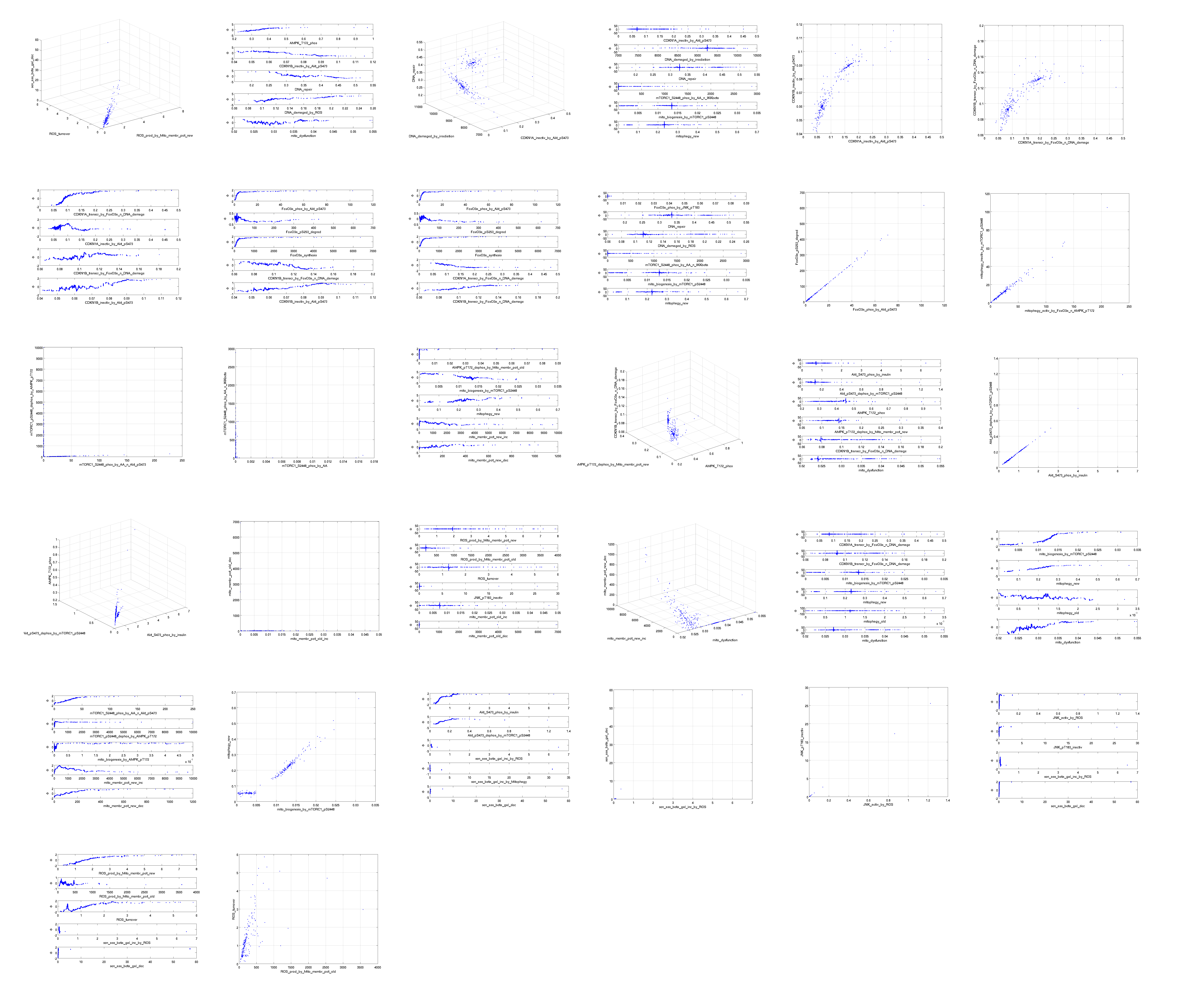

Supplement: Figure S5 — Correlation plots for round 1 of parameter estimation, as detected by MOTA identifiability analysis. Plots for the tuples of related parameters reported in the MOTA identifiability matrix in Figure S4. (TIF) [file pcbi.1003728.s005.tif]

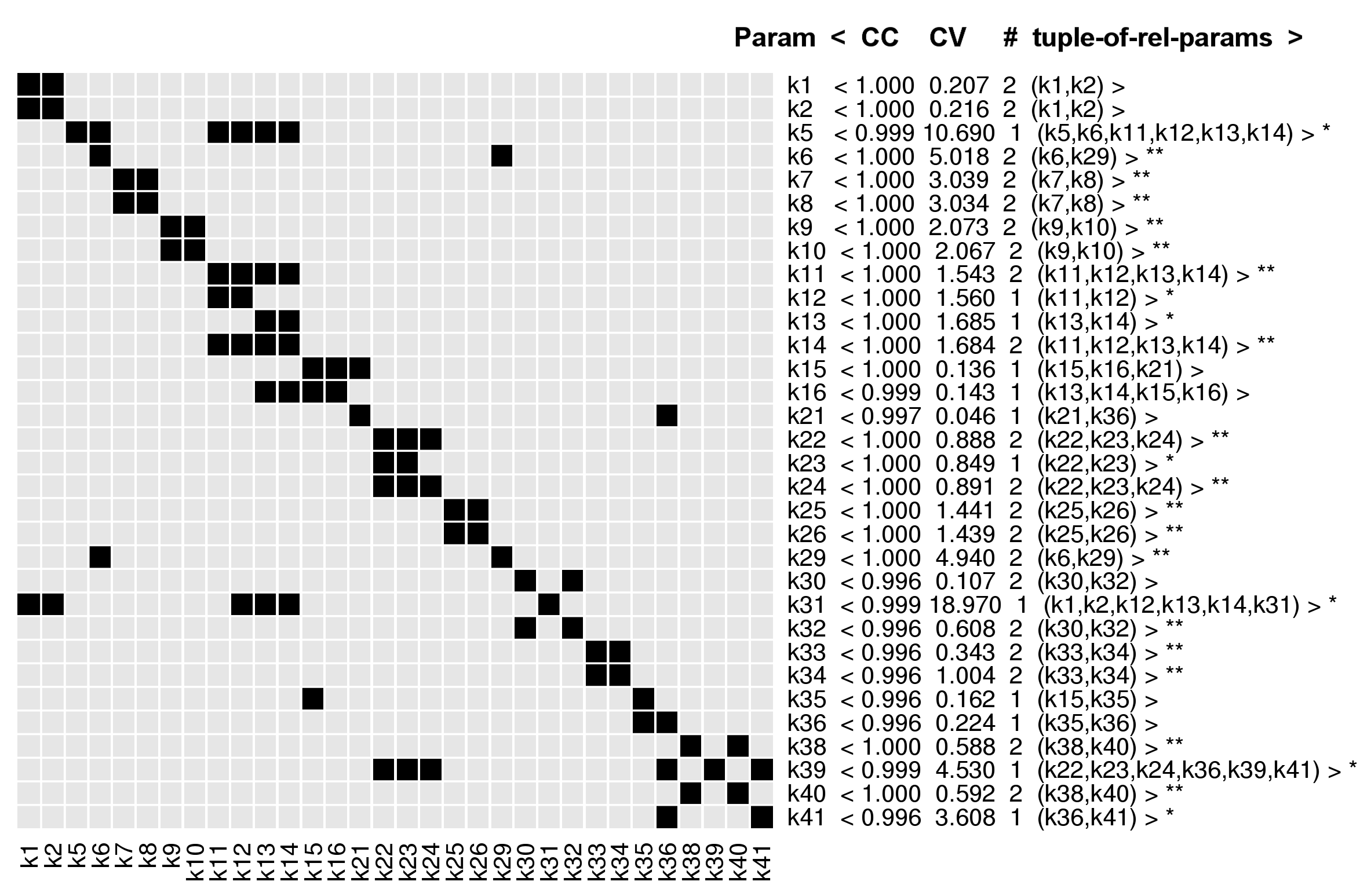

Supplement: Figure S6 — MOTA identifiability matrix for round 2 of parameter estimation. In this round the kinetic rate constants k1, k2, k15, k16, k21, k30, k35, and k36 were fixed since they were identifiable using MOTA identifiability analysis. (TIF) [file pcbi.1003728.s006.tif]

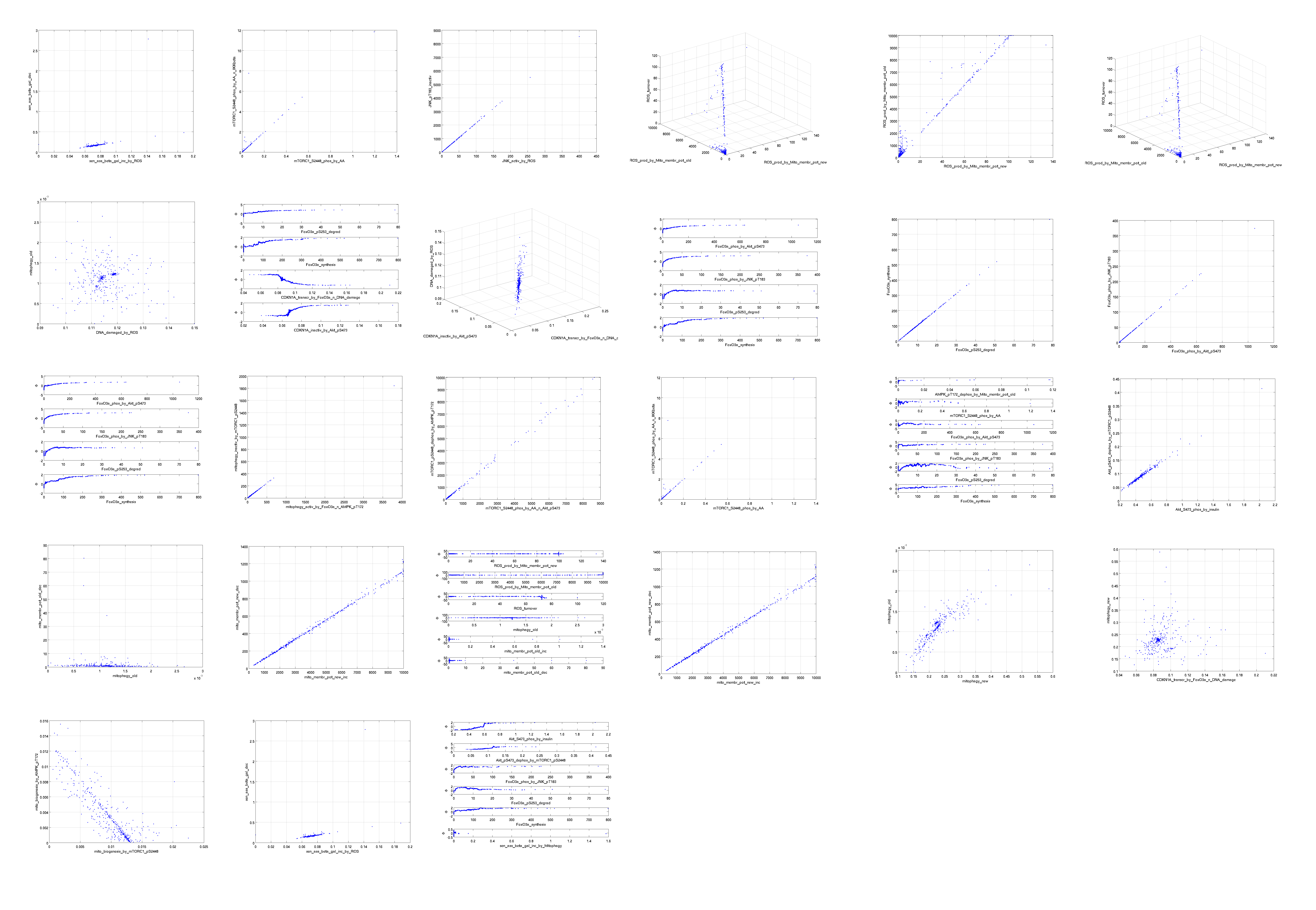

Supplement: Figure S7 — Correlation plots for round 2 of parameter estimation, as detected by MOTA identifiability analysis. Plots for the tuples of related parameters reported in the MOTA identifiability matrix in Figure S6. (TIF) [file pcbi.1003728.s007.tif]

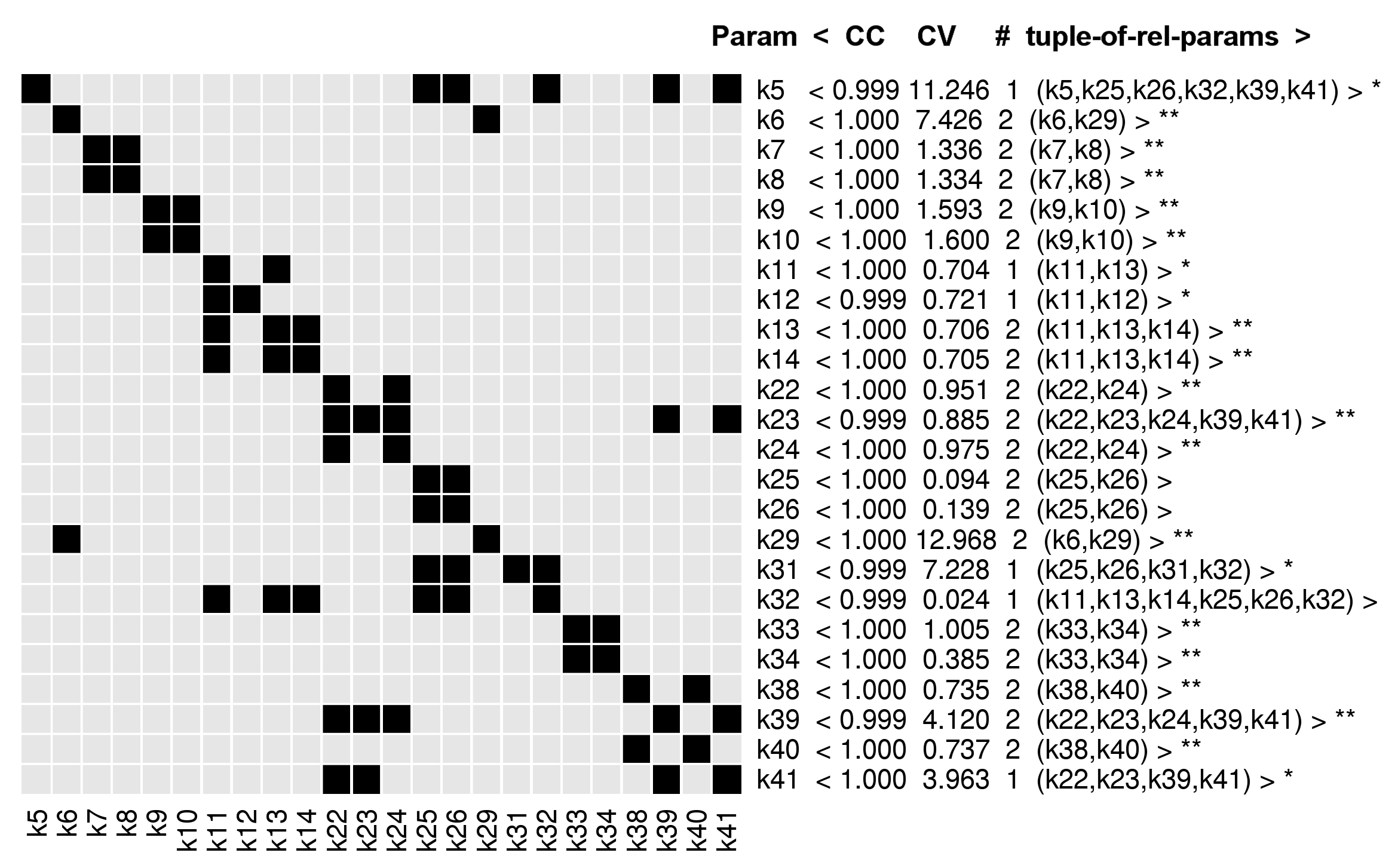

Supplement: Figure S8 — MOTA identifiability matrix for round 3 of parameter estimation. In this round the kinetic rate constants k25, k26, and k32 were fixed since they were identifiable using MOTA identifiability analysis. The parameters k8, k9, and k12 were locked as reported in Table S4. The parameters k8 and k9 were part of two couples of related parameters. The former k8 with k7, the latter k9 with k10. All these two couples only related internally with themselves and therefore formed two locally defined correlations. The parameter k12 only related with k11, although k11 was not dependent on k12. Hence, this also formed a locally defined correlation. (TIF) [file pcbi.1003728.s008.tif]

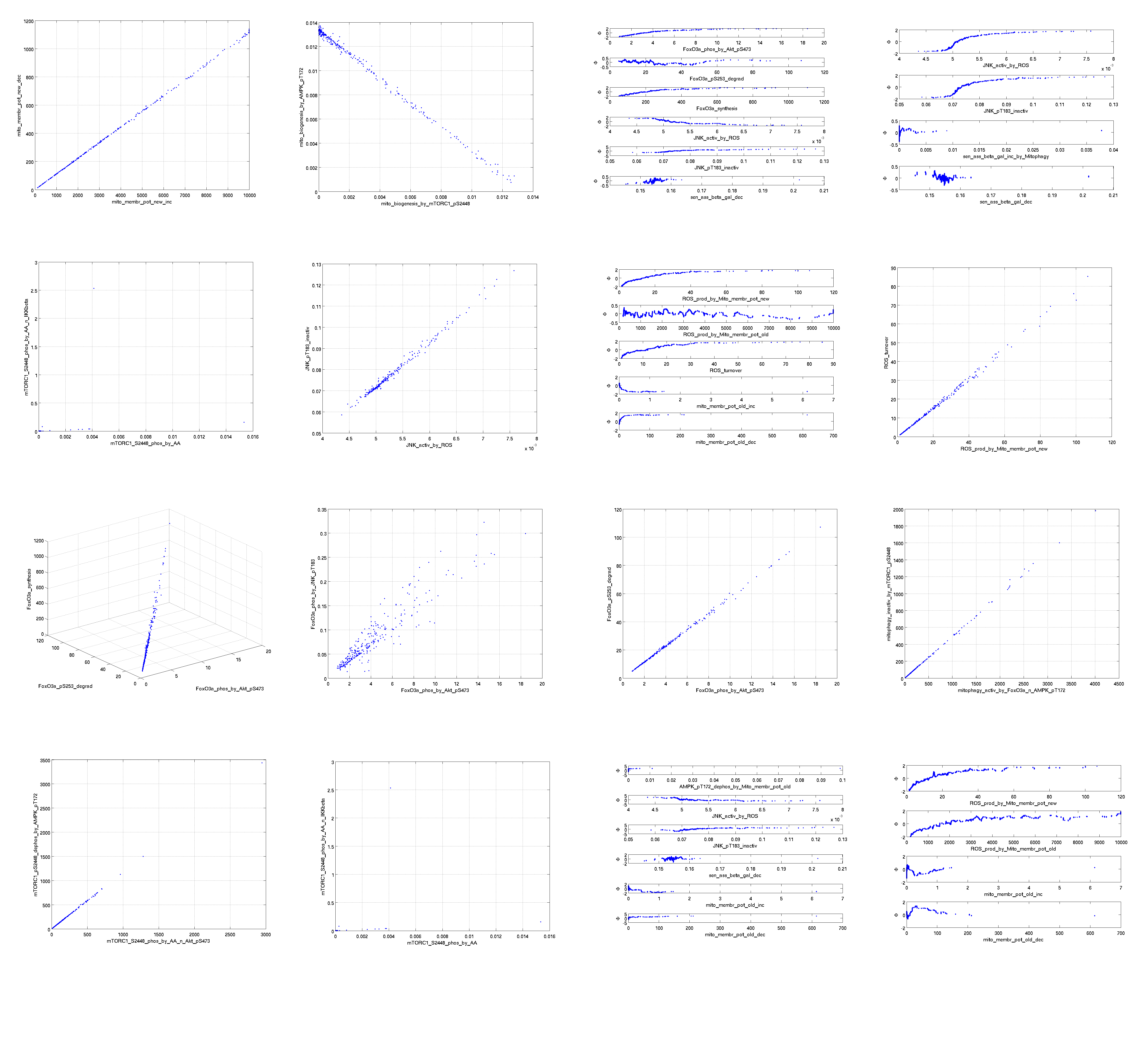

Supplement: Figure S9 — Correlation plots for round 3 of parameter estimation, as detected by MOTA identifiability analysis. Plots for the tuples of related parameters reported in the MOTA identifiability matrix in Figure S8. (TIF) [file pcbi.1003728.s009.tif]

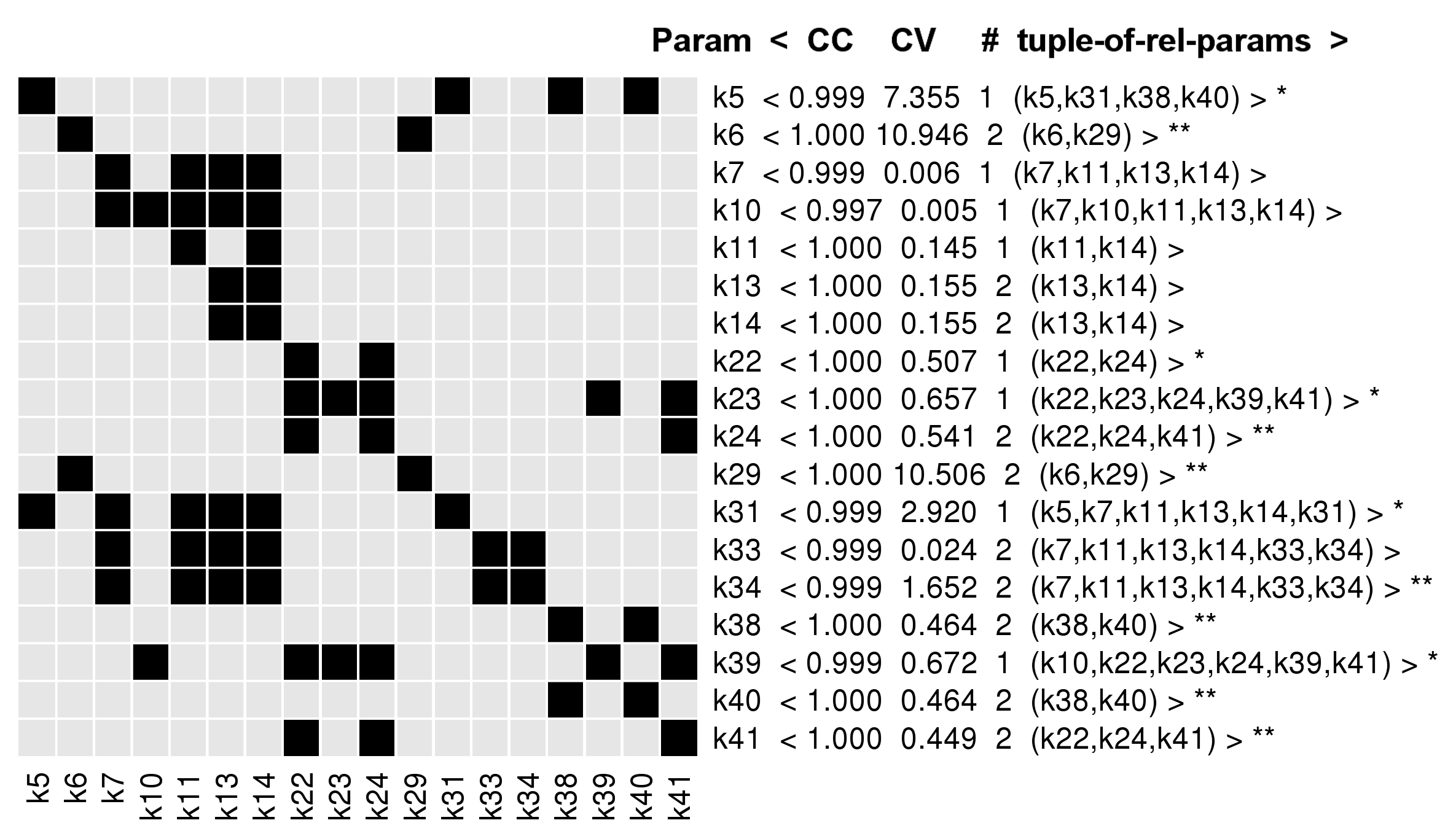

Supplement: Figure S10 — MOTA identifiability matrix for round 4 of parameter estimation. In this round the kinetic rate constants k7, k10, k11, k13, k14, and k33 were fixed since they were identifiable using MOTA identifiability analysis. (TIF) [file pcbi.1003728.s010.tif]

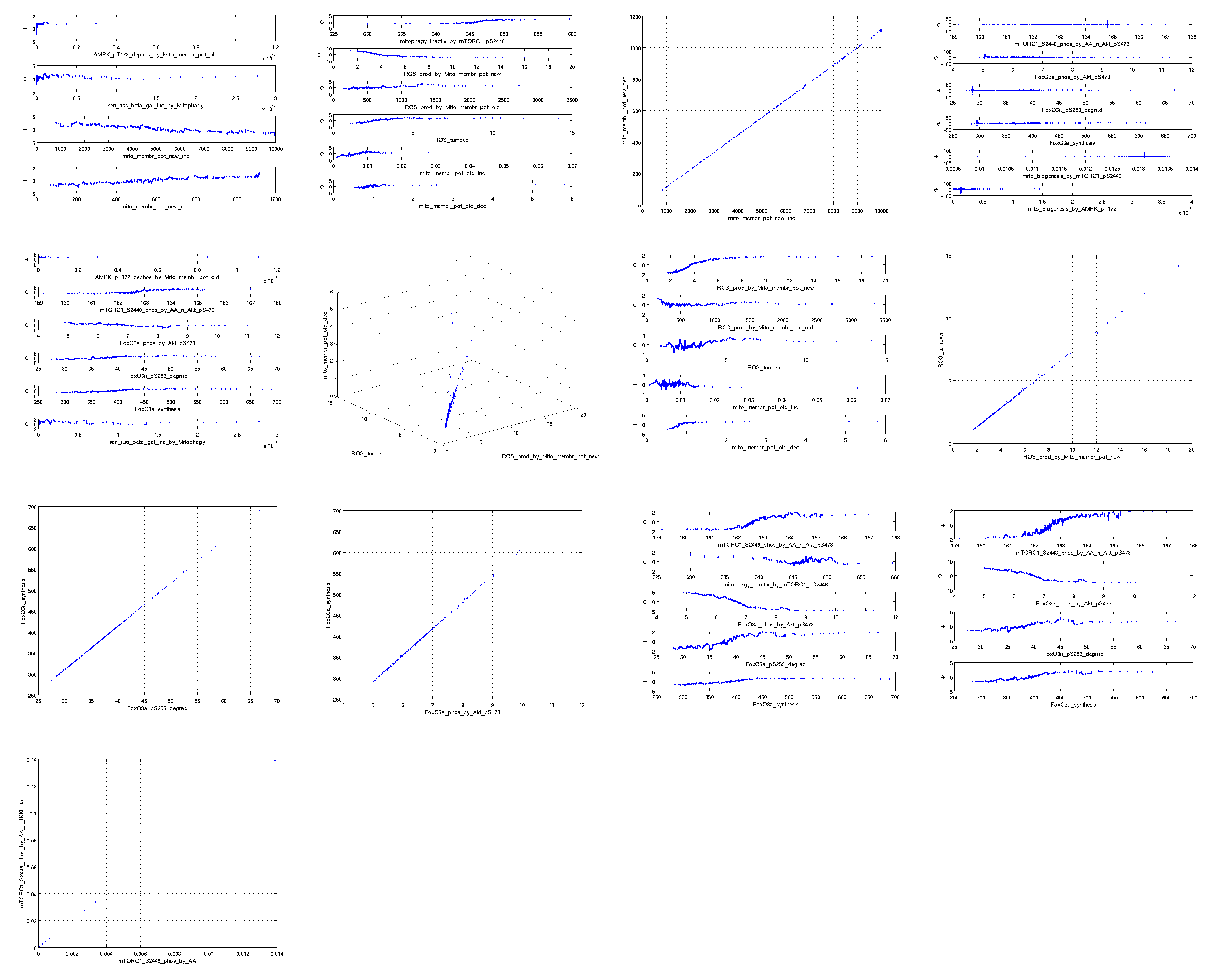

Supplement: Figure S11 — Correlation plots for round 4 of parameter estimation, as detected by MOTA identifiability analysis. Plots for the tuples of related parameters reported in the MOTA identifiability matrix in Figure S10. (TIF) [file pcbi.1003728.s011.tif]

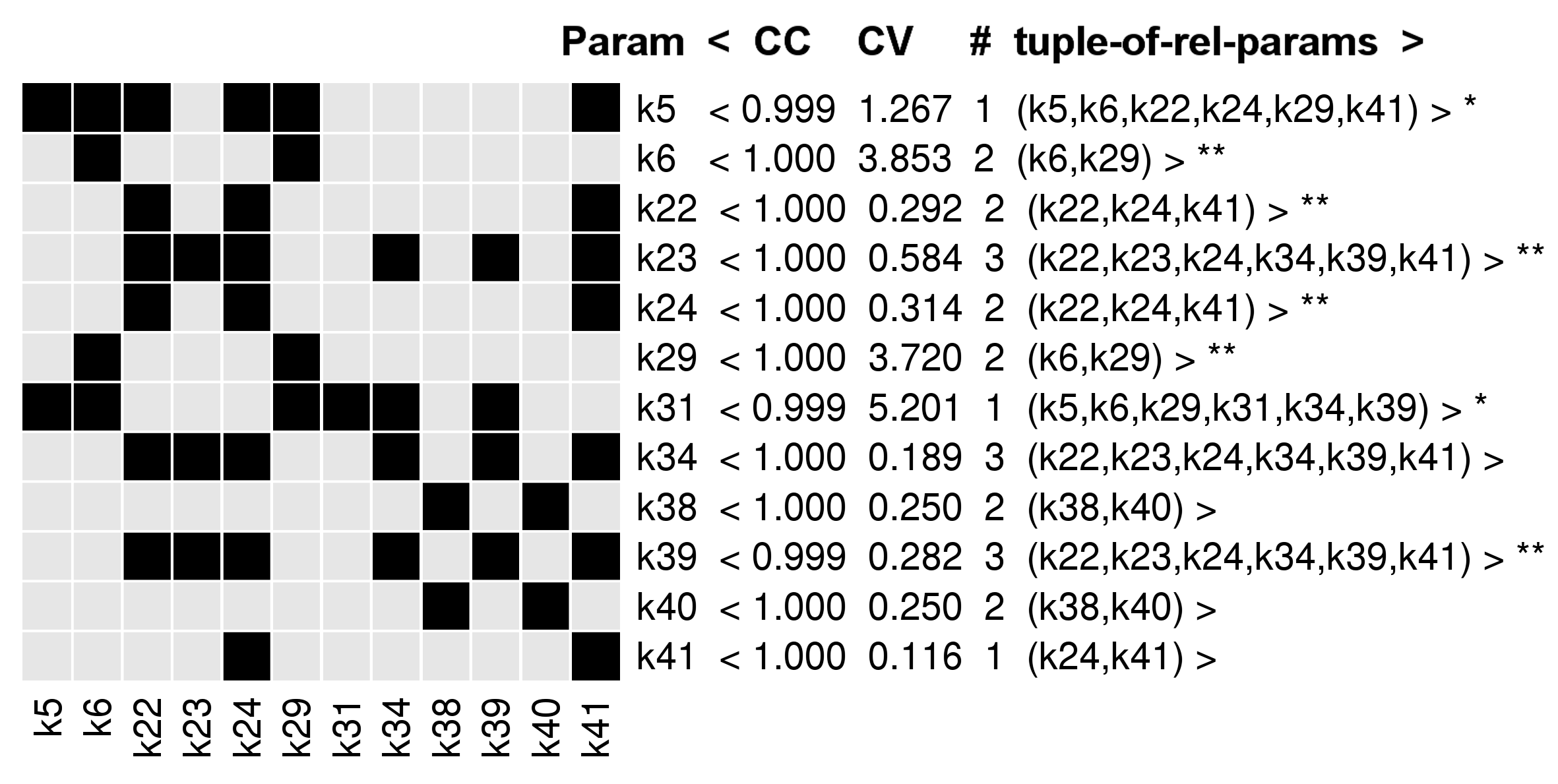

Supplement: Figure S12 — MOTA identifiability matrix for round 5 of parameter estimation. In this round the kinetic rate constants k34, k38, k40, and k41 were fixed since they were identifiable using MOTA identifiability analysis. (TIF) [file pcbi.1003728.s012.tif]

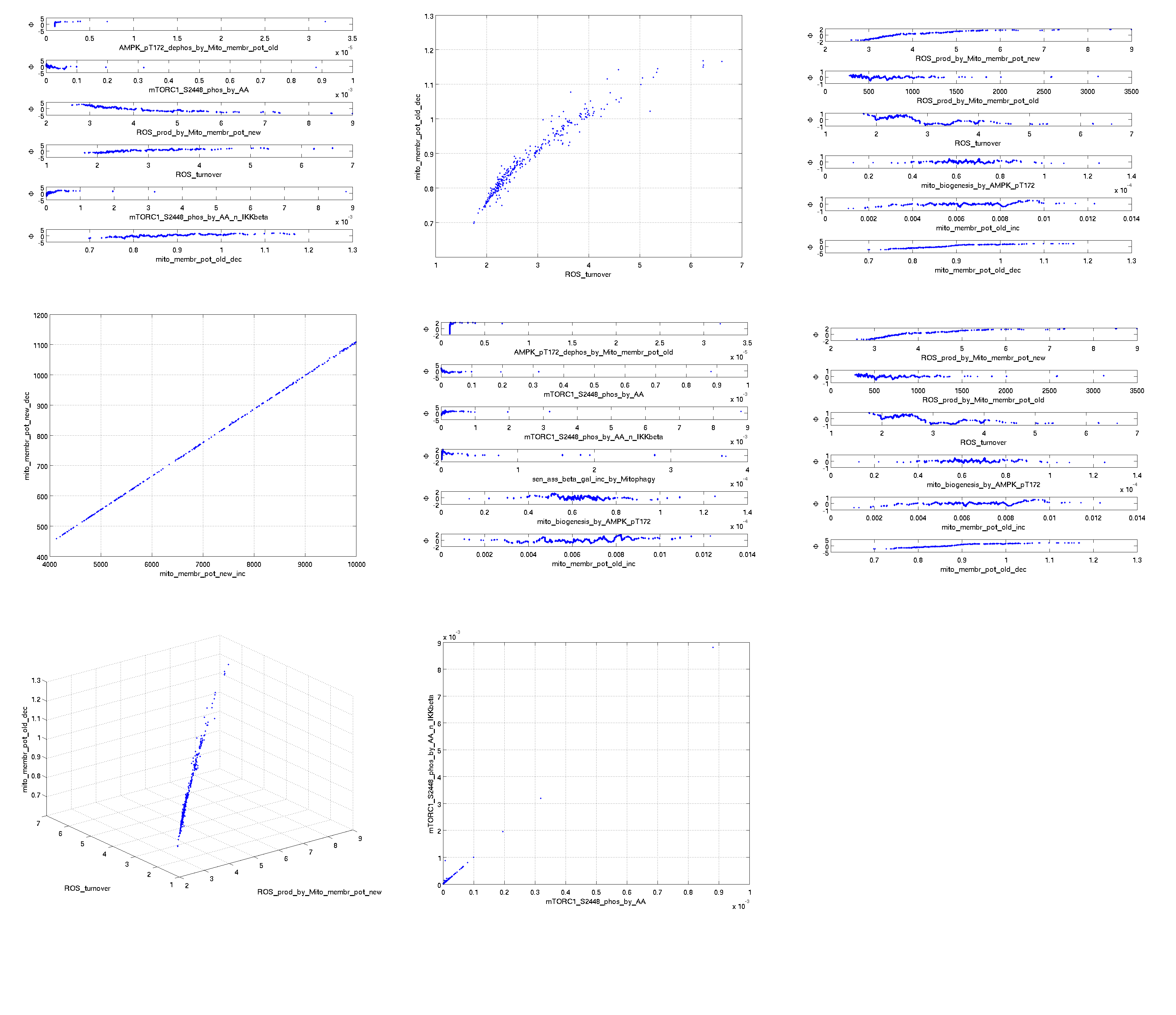

Supplement: Figure S13 — Correlation plots for round 5 of parameter estimation, as detected by MOTA identifiability analysis. Plots for the tuples of related parameters reported in the MOTA identifiability matrix in Figure S12. (TIF) [file pcbi.1003728.s013.tif]

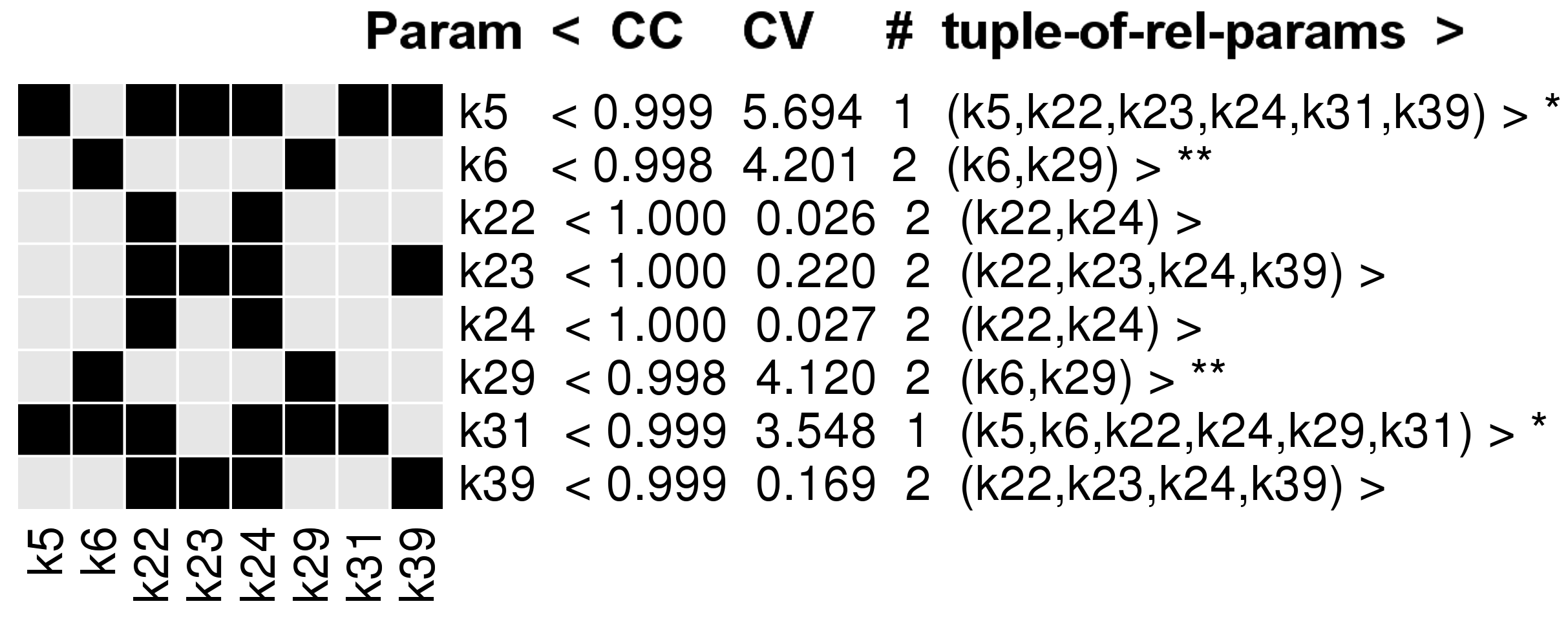

Supplement: Figure S14 — MOTA identifiability matrix for round 6 of parameter estimation. In this round the kinetic rate constants k22, k23, k24, and k39 were fixed since they were identifiable using MOTA identifiability analysis. (TIF) [file pcbi.1003728.s014.tif]

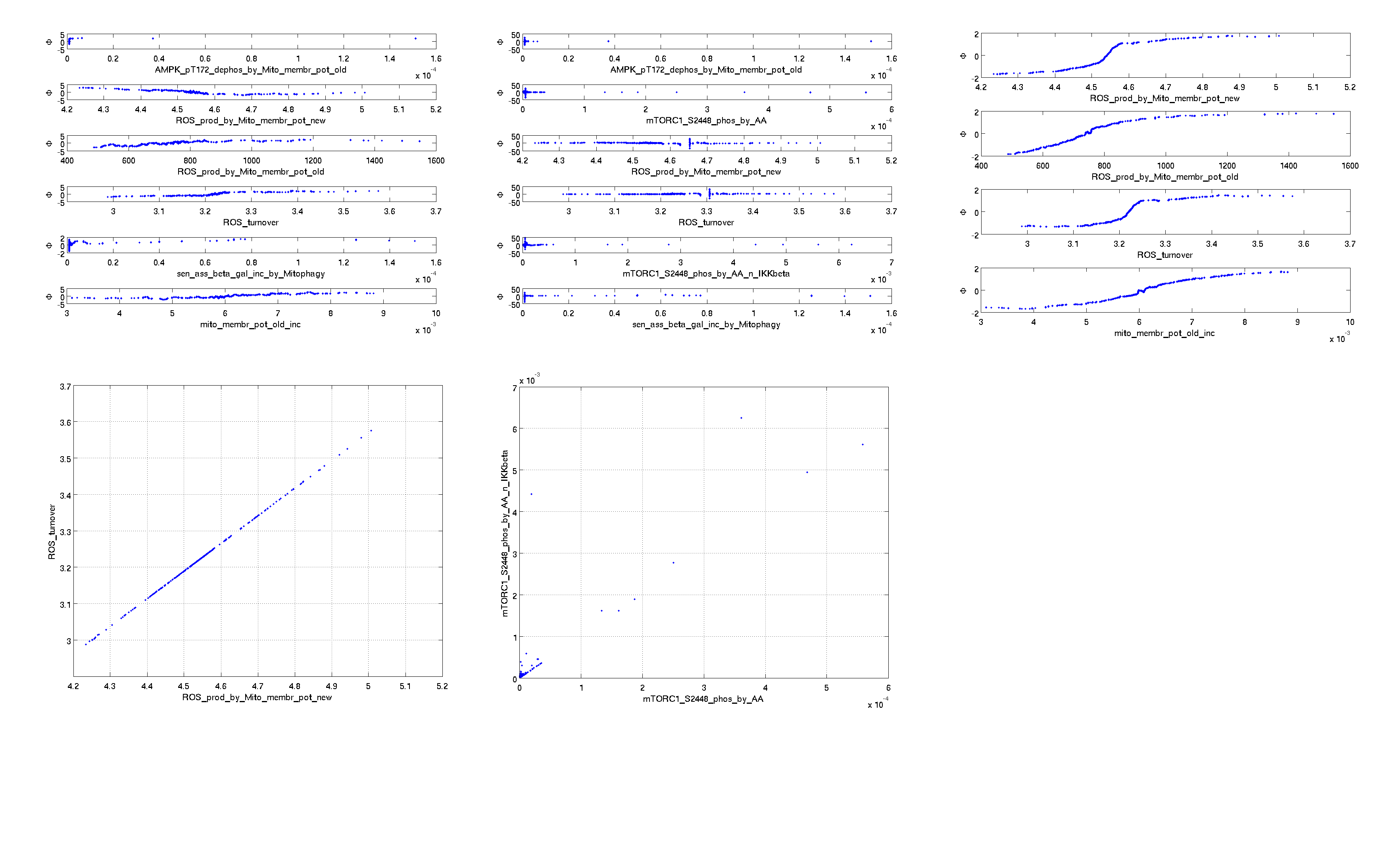

Supplement: Figure S15 — Correlation plots for round 6 of parameter estimation, as detected by MOTA identifiability analysis. Plots for the tuples of related parameters reported in the MOTA identifiability matrix in Figure S14. (TIF) [file pcbi.1003728.s015.tif]

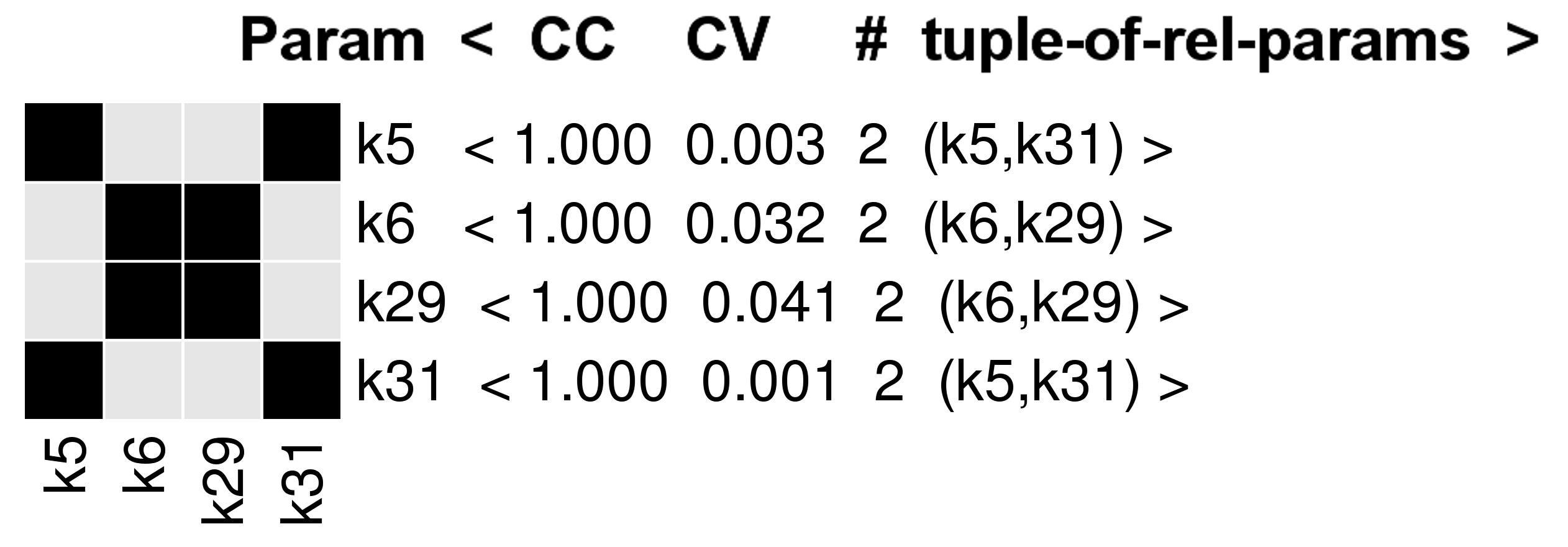

Supplement: Figure S16 — MOTA identifiability matrix for round 7 of parameter estimation. In this round the kinetic rate constants k5, k6, k29, and k31 were fixed since they were identifiable using MOTA identifiability analysis. (TIF) [file pcbi.1003728.s016.tif]

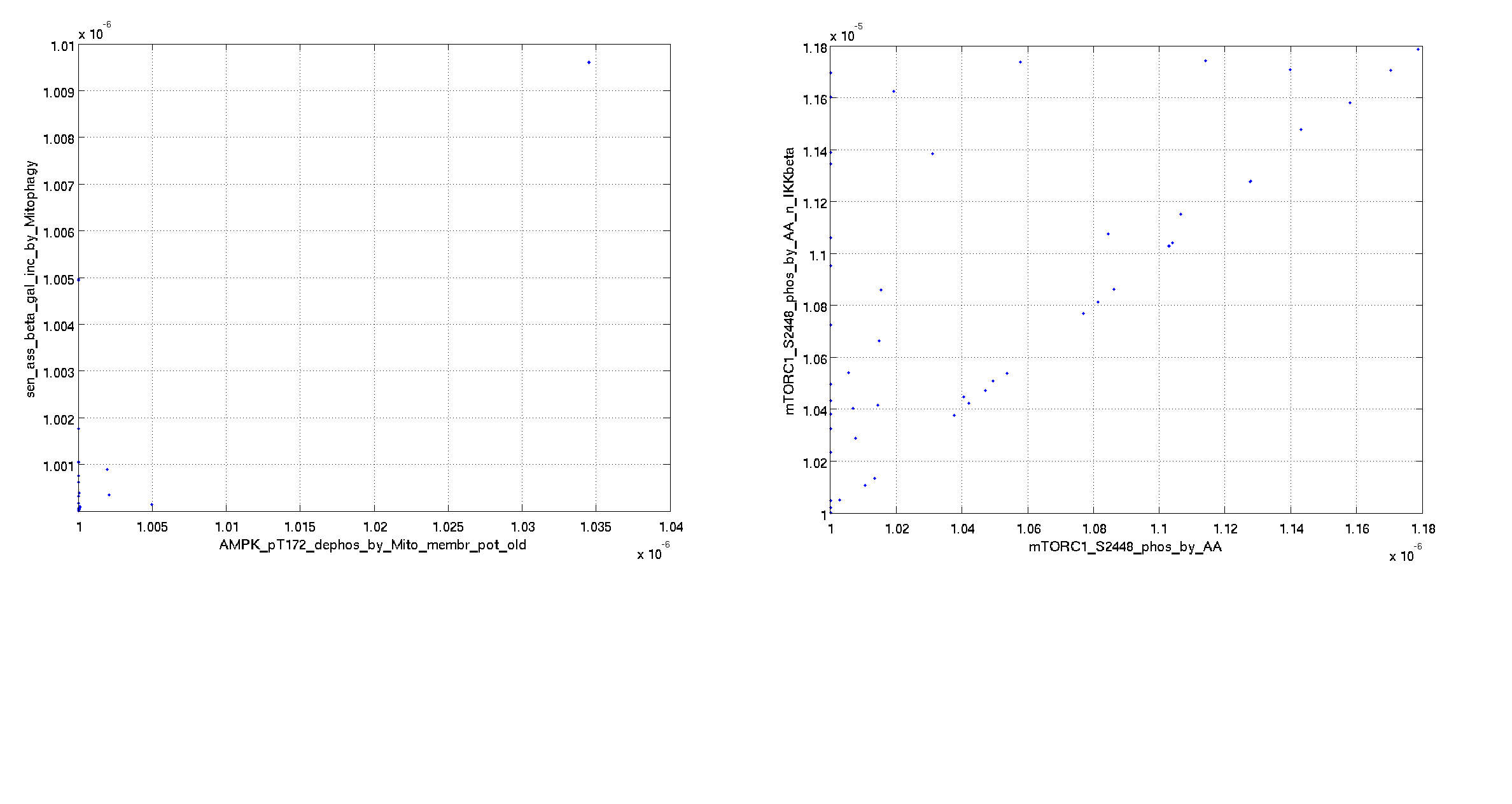

Supplement: Figure S17 — Correlation plots for round 7 of parameter estimation, as detected by MOTA identifiability analysis. Plots for the tuples of related parameters reported in the MOTA identifiability matrix in Figure S16. (TIF) [file pcbi.1003728.s017.tif]

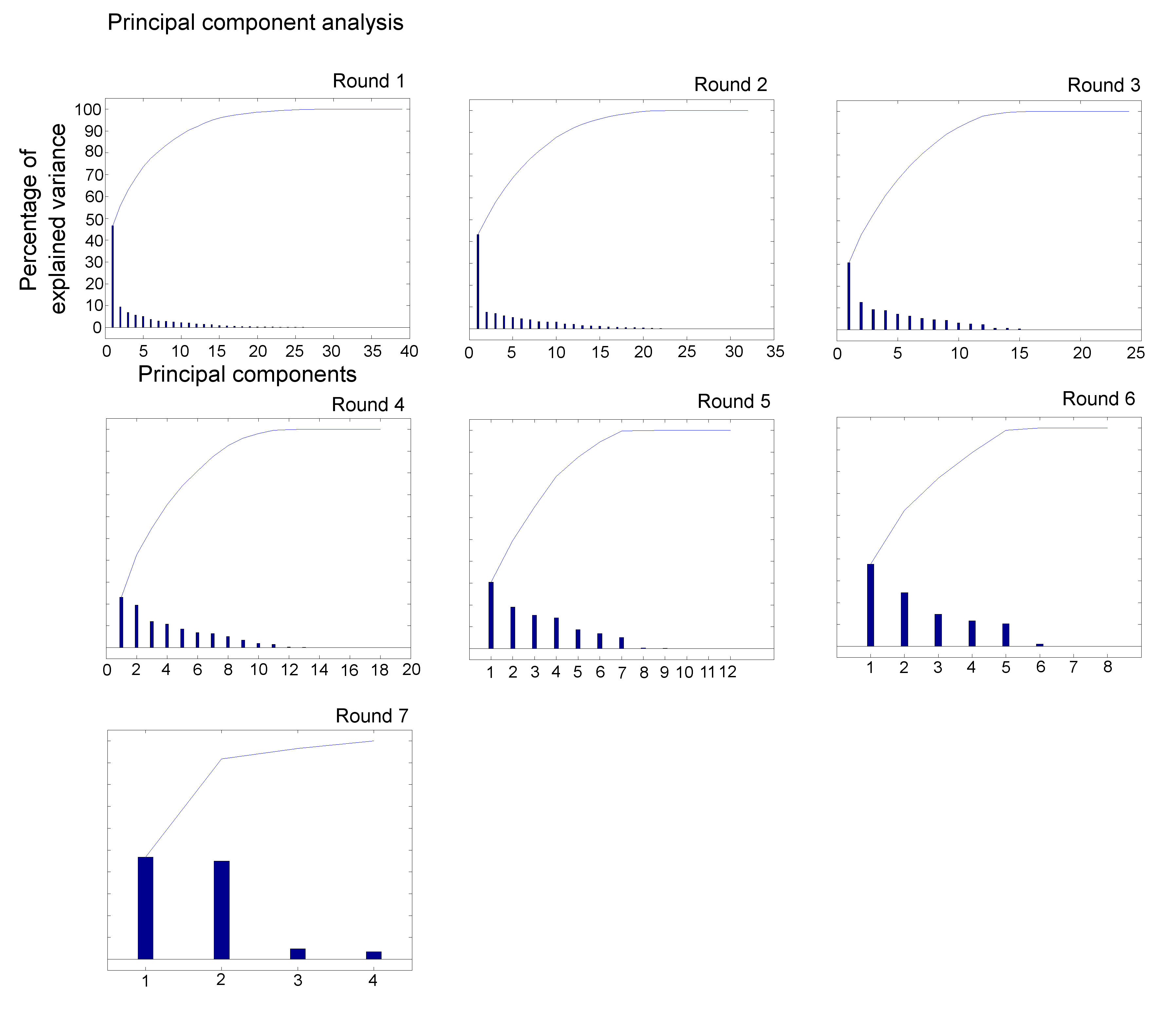

Supplement: Figure S18 — Principal component analysis for model at each round of parameter estimation. To further investigate the source of variability, principal component analysis (PCA) of the model was computed at each round of parameter estimation. Interestingly, this analysis indicated that only about half of the estimated parameters did not significantly contribute to the overall variance for each round. This suggested that a group of parameters could have been potentially identified at each round. (TIF) [file pcbi.1003728.s018.tif]

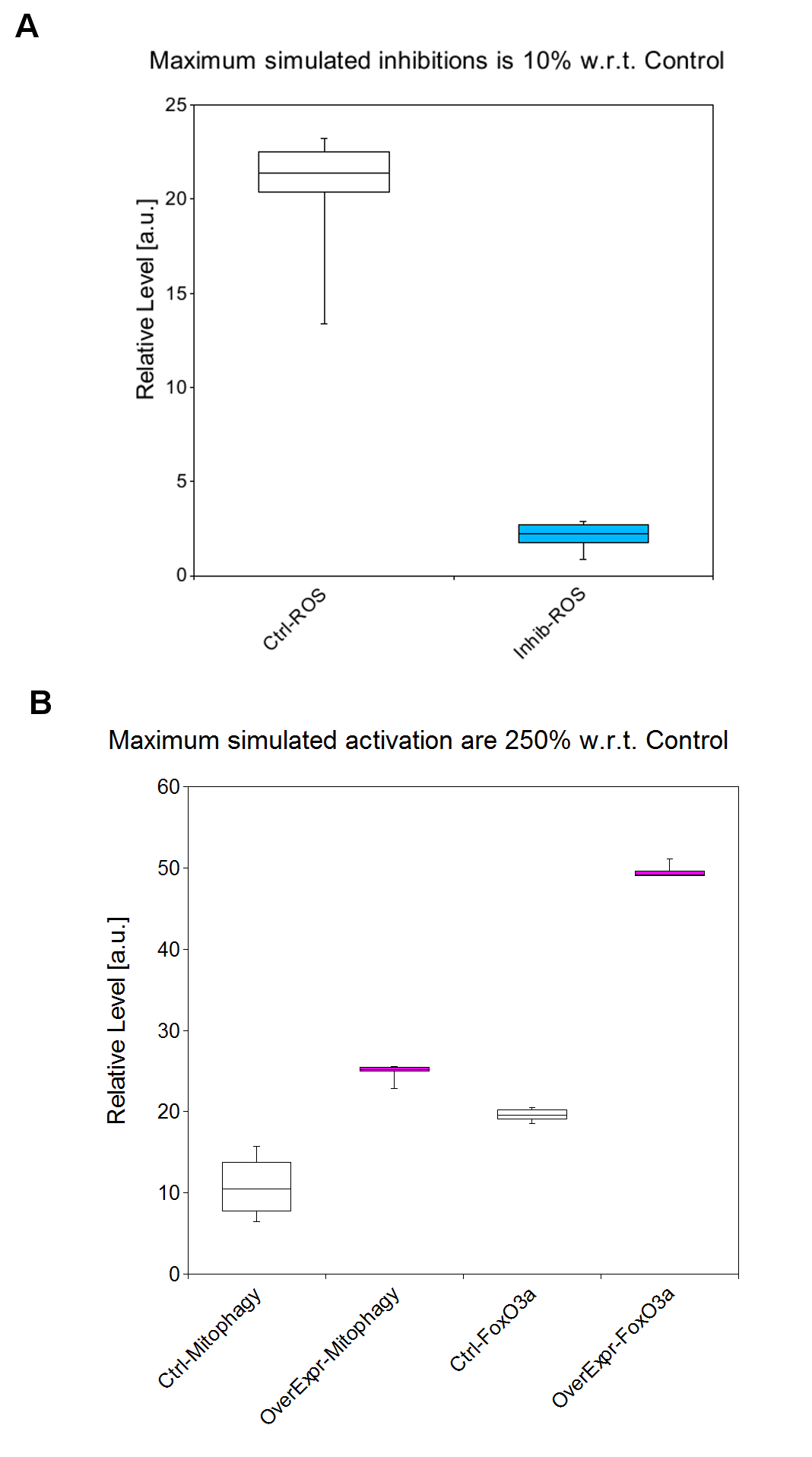

Supplement: Figure S19 — Simulated tools for model inhibition or over-activation over time. (A) To inhibit ROS over time, a simulated ROS inhibitor species was added to the model. This new species reduced ROS levels and acted as an in vitro ROS scavenger. The abundance for this species was estimated in order to achieve ROS inhibition to 10% (blue) as compared to the control (white) throughout the time course. (B) In analogy, two new species were created for over-activating mitophagy and FoxO3a, respectively. The abundance for these two species were estimated to achieve a mitophagy or FoxO3a over-activation of 150% (magenta) as compared to the corresponding control (white) throughout the time course. Each boxplot represents the median and two quartiles, whereas the bars indicate the minimum and the maximum values, as estimated from day 1 to day 21. (TIF) [file pcbi.1003728.s019.tif]

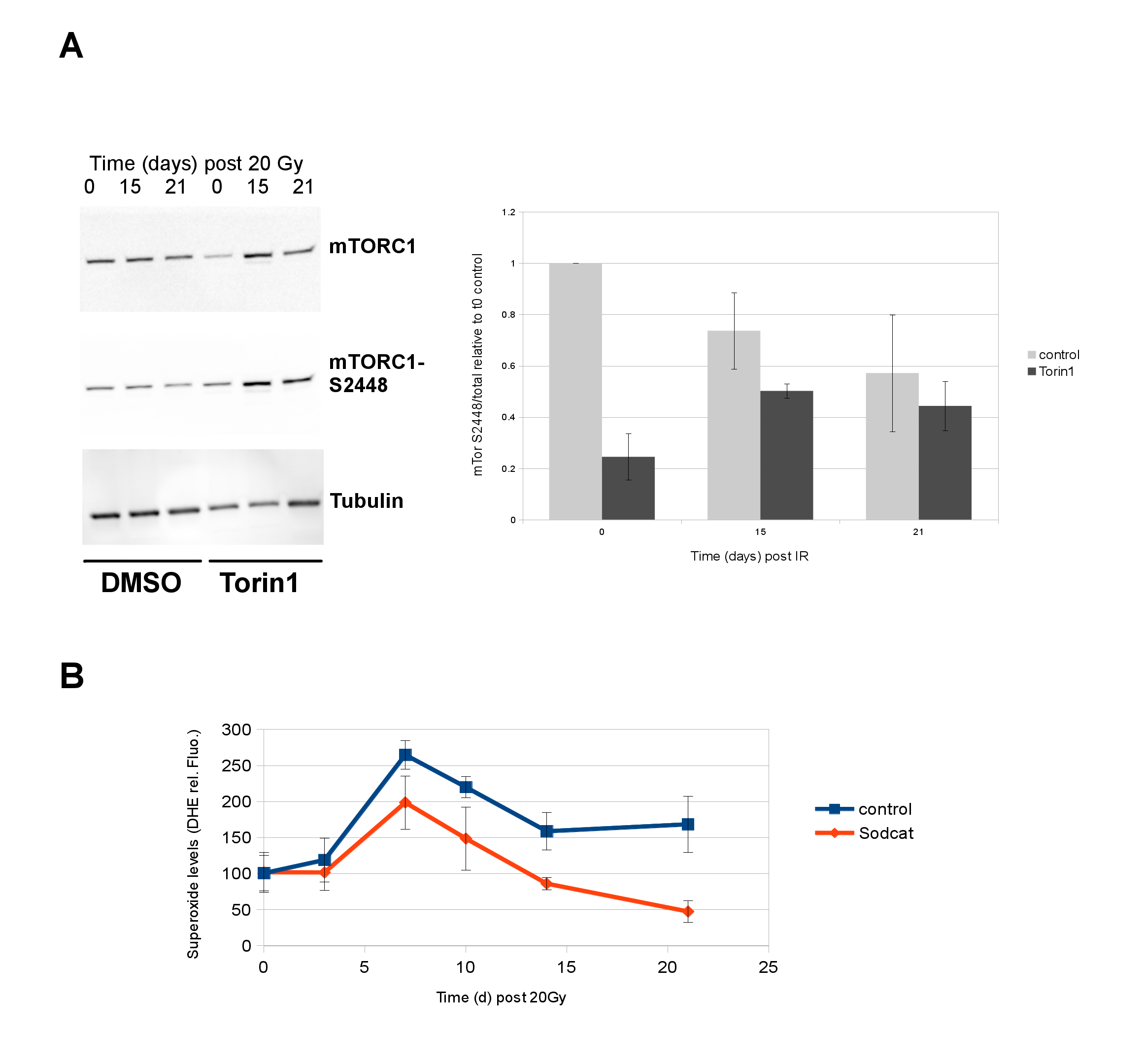

Supplement: Figure S20 — Inhibition of ROS and mTOR in vitro. Torin and ROS inhibition efficacy. (A) Cells were irradiated with 20 Gy X irradiation and then treated with Torin1 or DMSO as described in Methods. Lysates were probed with total mTORC1 and mTORC1-S2448 antibodies. Band intensities were quantified relative to Tubulin loading control and plotted as mean +/− SD ratios of mTORC1-S2448 to total mTORC1 (3 repeats). (B) Cells were irradiated with 20 Gy X irradiation and then treated with SOD and catalase as described in Methods. Cells were stained with DHE to measure intracellular superoxide levels by flow cytometry. Time course data over 21 days are plotted (n = 3). (TIF) [file pcbi.1003728.s020.tif]

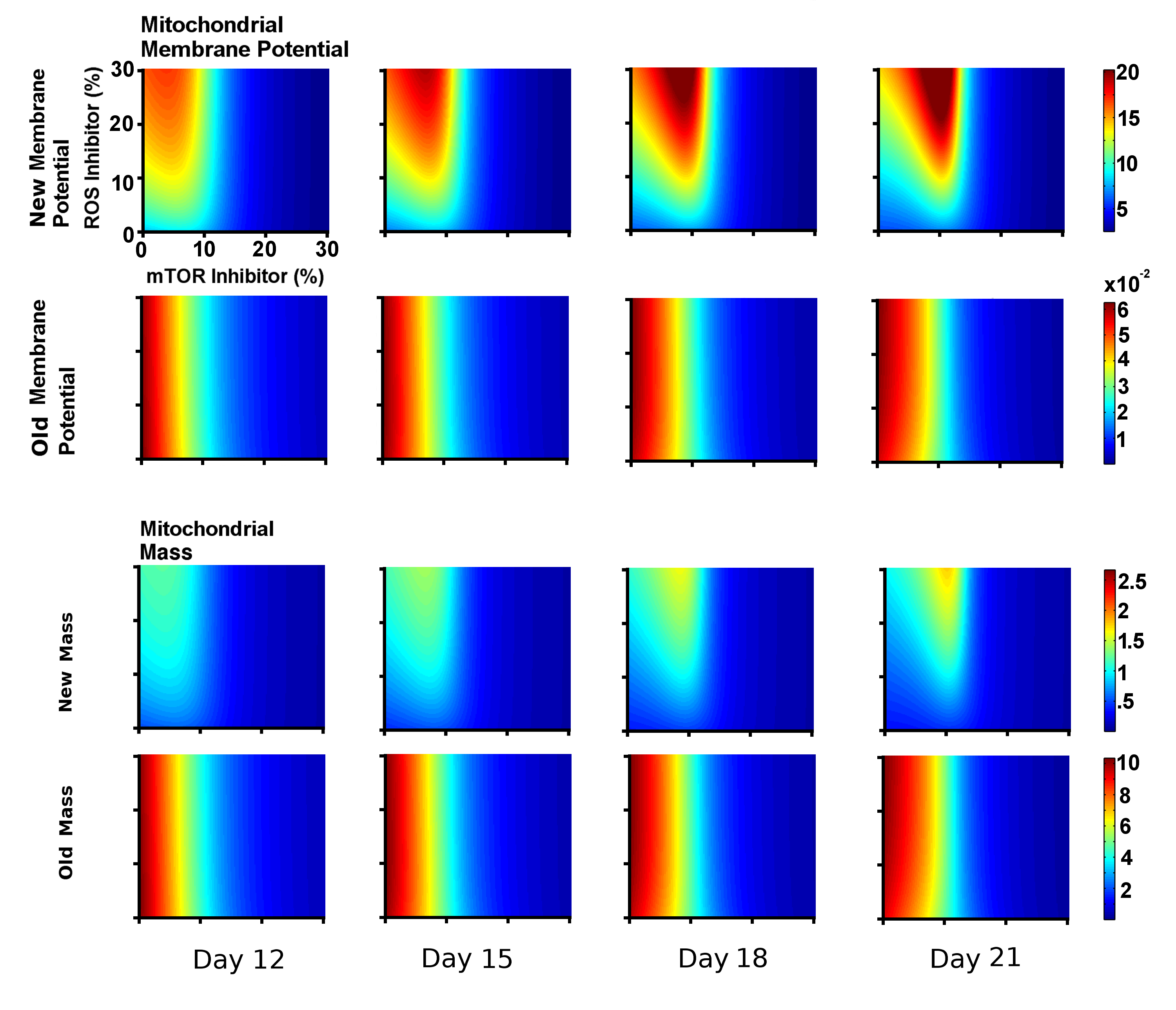

Supplement: Figure S21 — Analysis of the two mitochondrial sub-populations upon ROS-mTOR combined intervention. The internal states for the new and old mitochondrial sub-populations were also investigated upon combined ROS-mTOR simulated intervention. Regarding the mitochondrial membrane potential, the global effect of these interventions mainly resulted from changes in the sub-population of new mitochondria, which showed a strong synergistic response at particular levels of mTOR- ROS inhibition. The effect upon old mitochondrial øm was almost entirely dependent upon mTOR inhibition, but still changed their potential by an insignificant amount compared to the new mitochondrial population. Concerning the mitochondrial mass, the perturbation of combined ROS-mTOR acted predominantly on the young population, although these changes were largely hidden in the overall population (Figure 4A) due to the larger proportion being comprised of the old population. The point (0, 0) indicates the control (no inhibition). (TIF) [file pcbi.1003728.s021.tif]

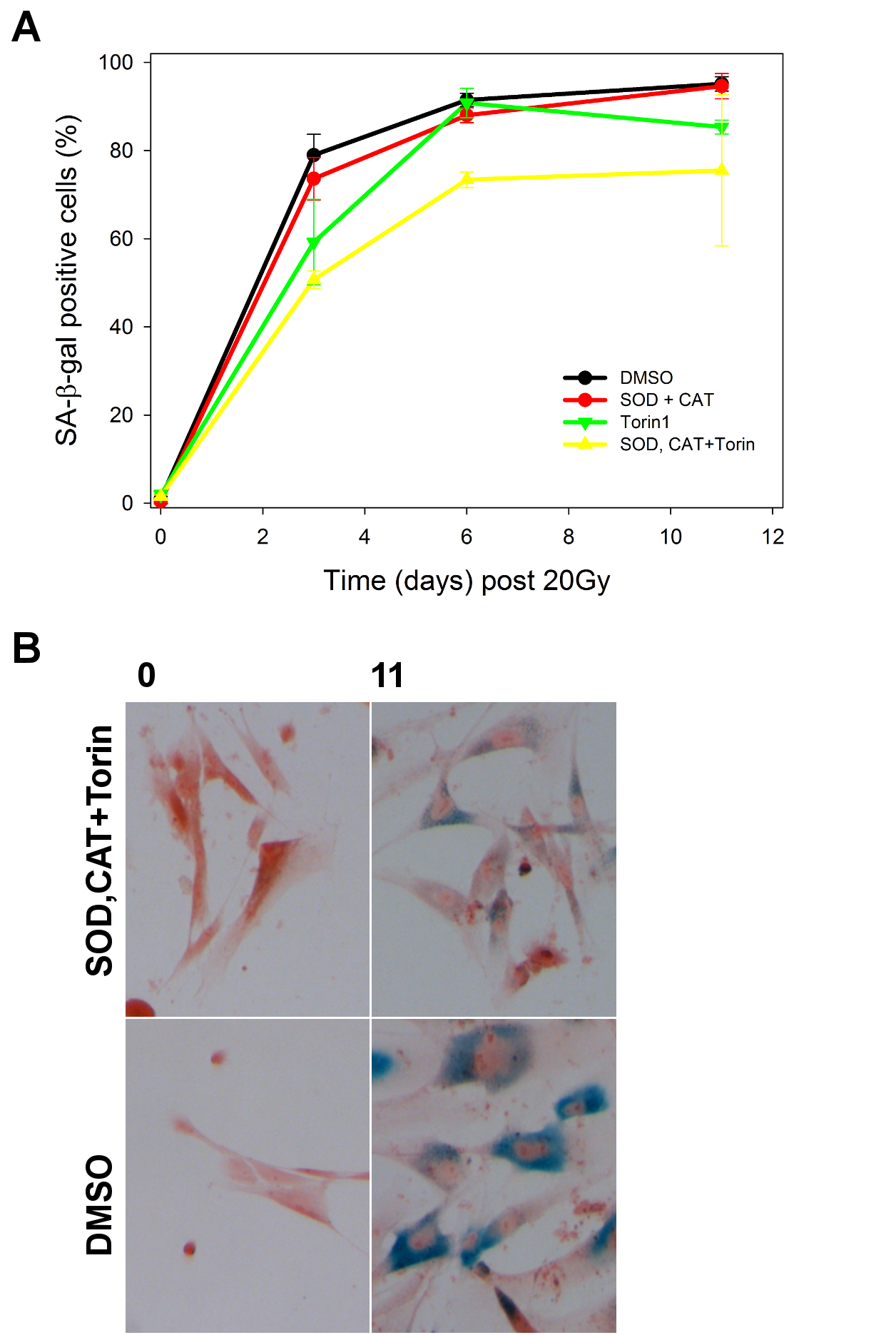

Supplement: Figure S22 — Analysis of senescence-associated β-galactosidase staining upon upon ROS-mTOR combined intervention. (A) Cells were fixed at the indicated timepoints and assayed for senescence–associated β-galactosidase followed by counterstaining with nuclear fast red prior to imaging. An average of ∼300 cells were counted per coverslip, with any blue cytoplasmic staining being considered positive. Data are n = 2 ± SD. Combined intervention produced significantly lower positive cells at 3 and 6 days post irradiation relative to DMSO control (P<0.05). (B) Example images of stained coverslips showing the decreased intensity of staining observed in treated cells. (TIF) [file pcbi.1003728.s022.tif]

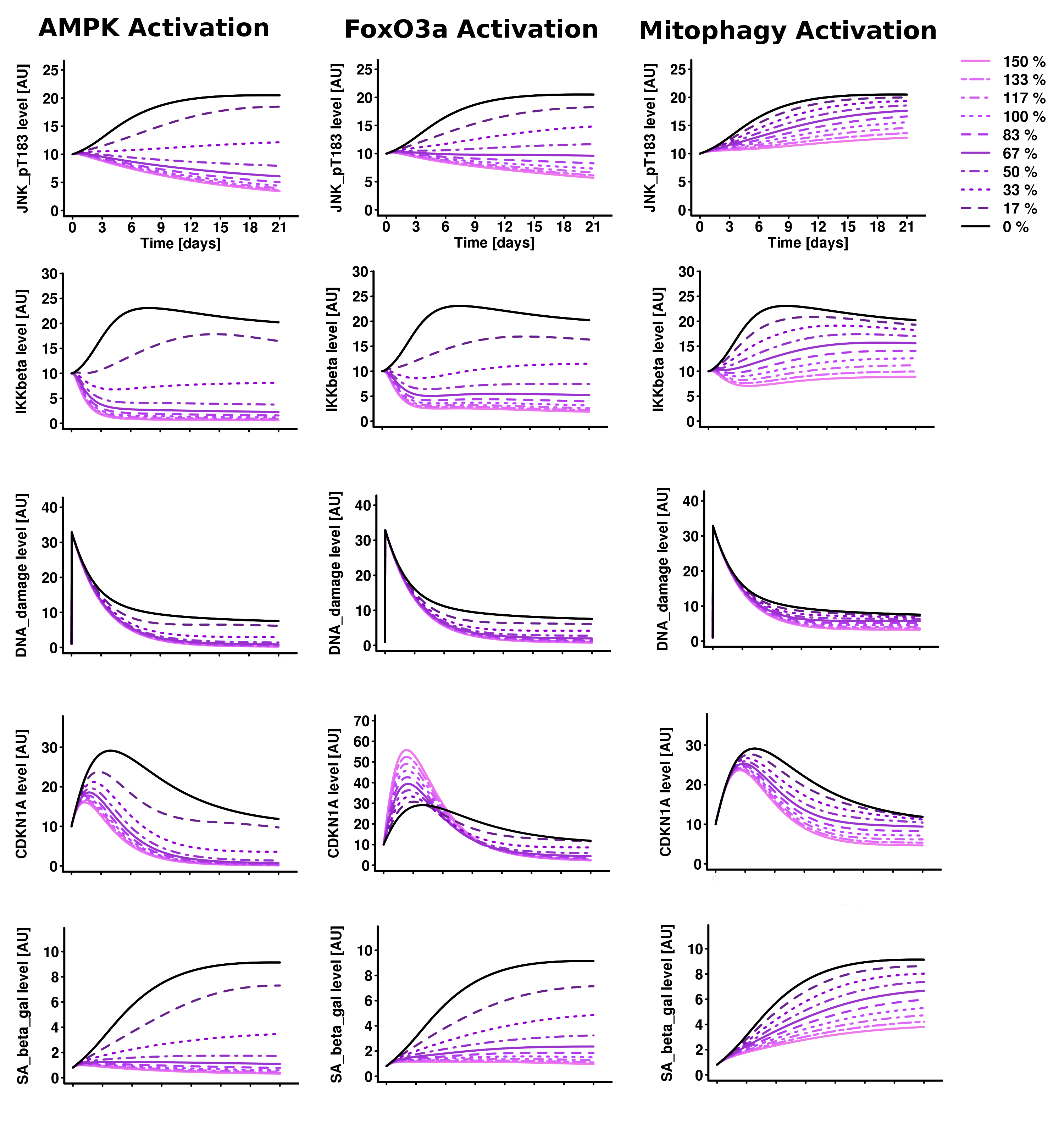

Supplement: Figure S23 — Additional readouts upon AMPK, FoxO3a or mitophagy simulated over-activation. The model predicted a decrease in the DNA damage/oxidative stress response pathways upon over-activation of AMPK, FoxO3a or mitophagy. AMPK was predicted to achieve the strongest inhibition throughout the time course as compared to FoxO3a or mitophagy. Interestingly, an over-activation of FoxO3a was predicted to initially increase and then reduce CDKN1A levels as compared to the control (black line), suggesting differential regulation of cell cycle arrest over time. (TIF) [file pcbi.1003728.s023.tif]

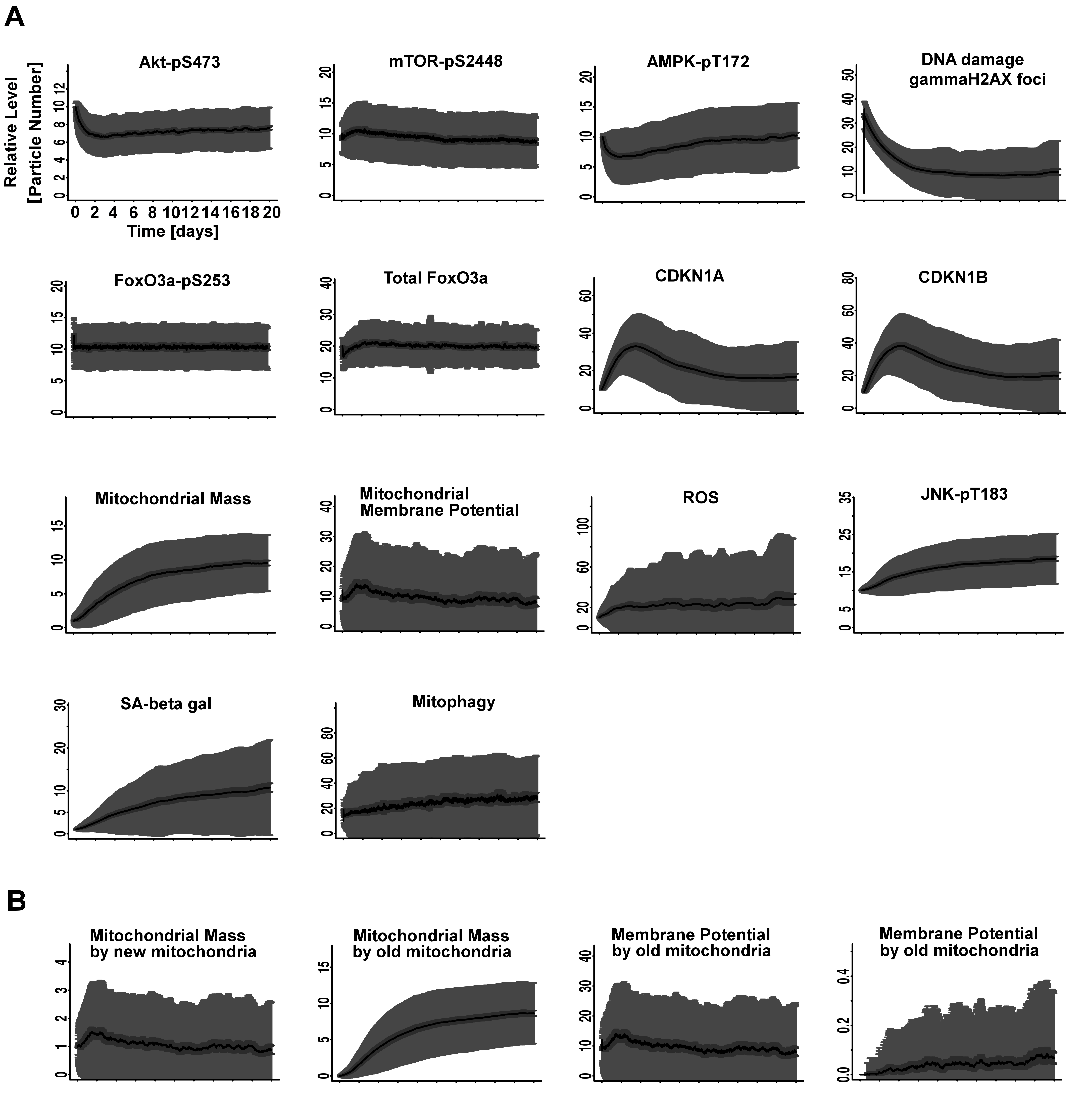

Supplement: Figure S24 — Model stochastic simulation showed increase stochasticity over time. Model stochastic simulations up to 20 days graphically showed increased stochasticity for the oxidative stress/DNA damage signalling species. Moderate increased variance was also detected for the species Mitophagy and the species representing mitochondrial mass and membrane potential. Number of stochastic runs: 500; black line indicates the means, dark grey area indicates 95% confidence interval of the mean and grey area indicates a standard deviation. (A) Species associated to in vitro data (observable variables). (B) Mitochondrial internal states (derived variables). (TIF) [file pcbi.1003728.s024.tif]

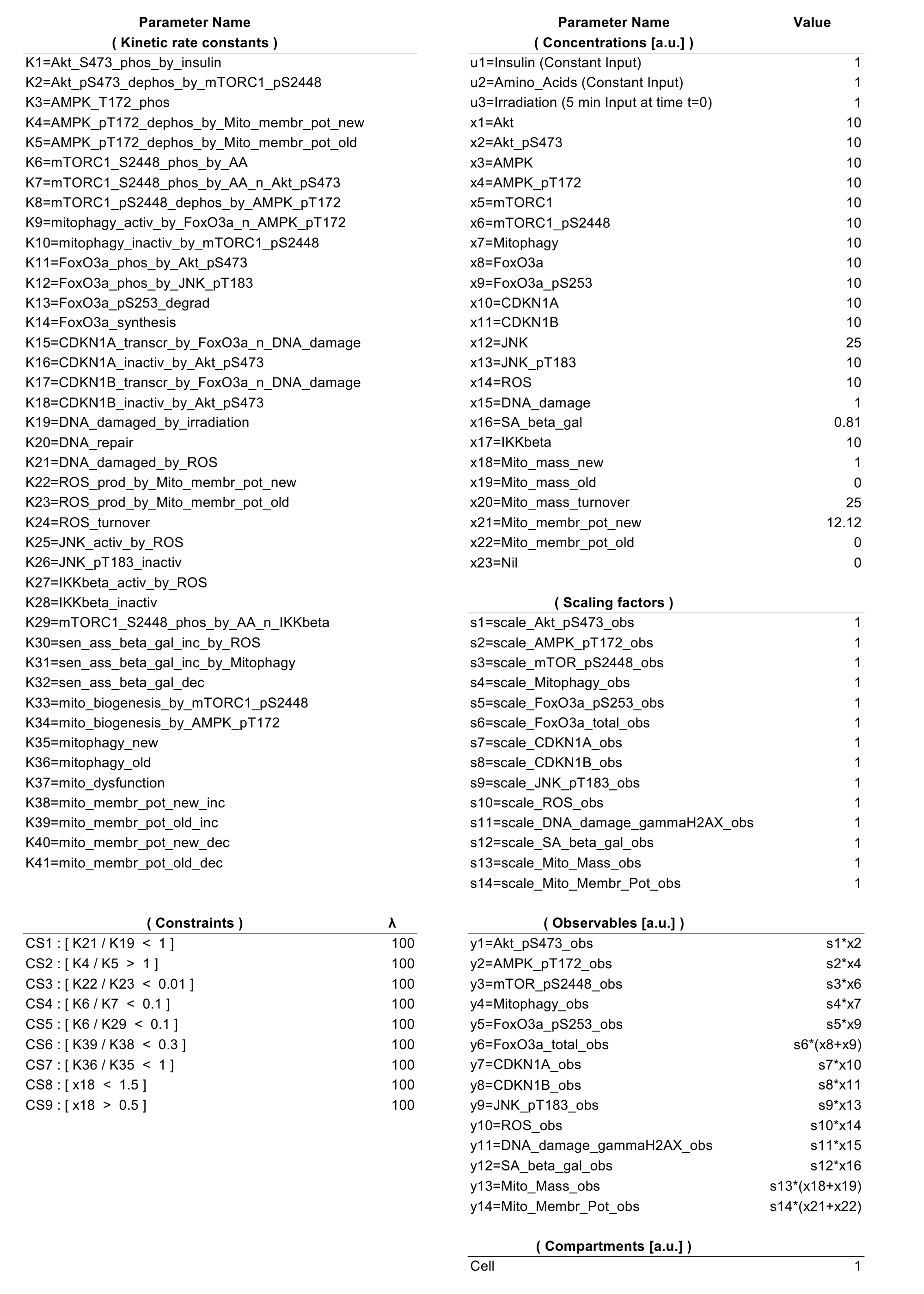

Supplement: Table S2 — Legend of the model variables. This table defines the unique codes for the model kinetic rate constants (Ki), species (Xi), scaling factors (Si), observables (Yi) and constraints (CSi). The values for the non-estimated parameters are also provided. The lambda term in the block ‘Constraints’ defines the constraint strength (higher values means harder constraints) as implemented in PottersWheel. (TIF) [file pcbi.1003728.s029.tif]

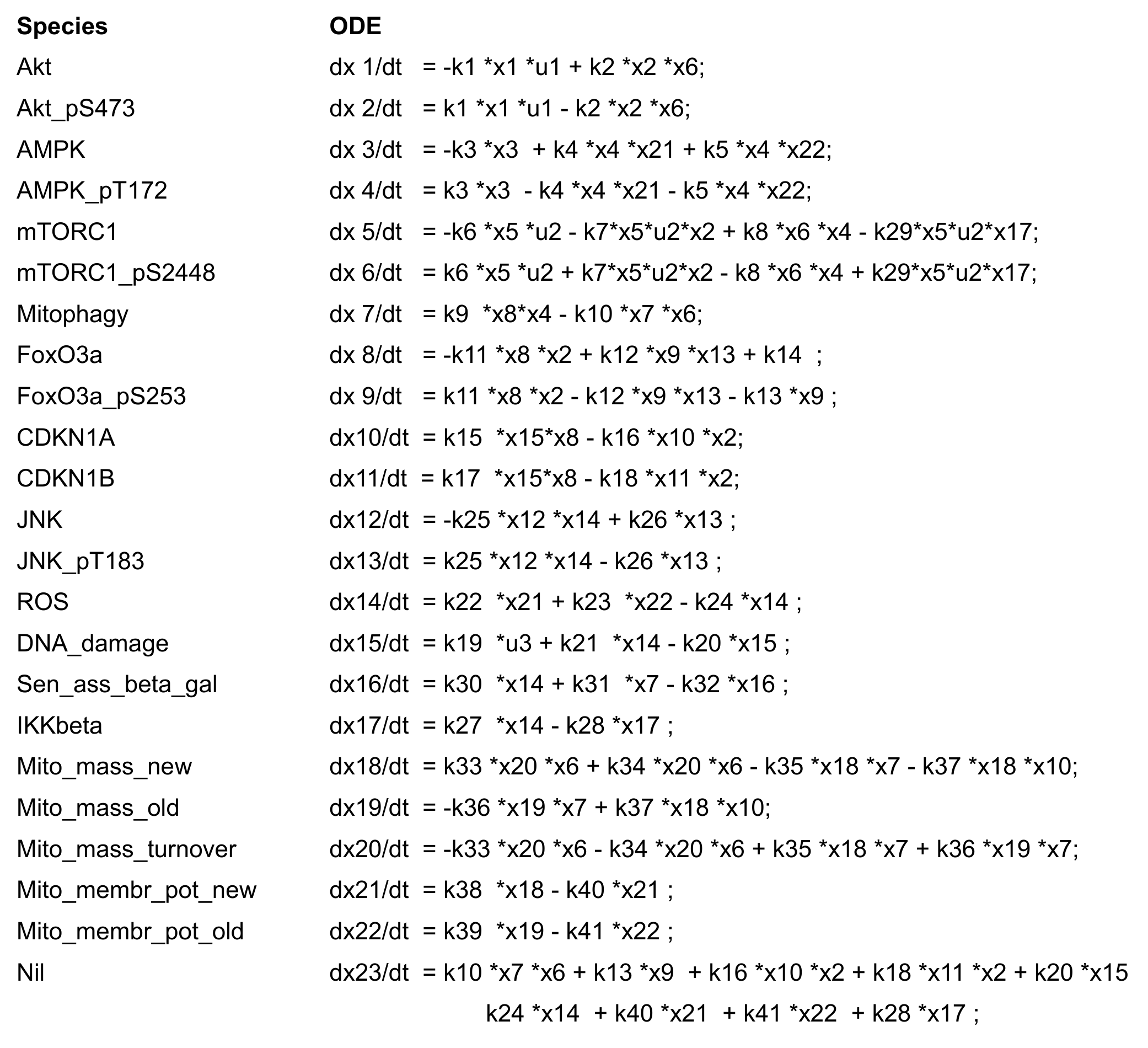

Supplement: Table S3 — ODEs table of the model. The Ordinary Differential Equations (ODEs) for the 23 species defining the model. (TIF) [file pcbi.1003728.s030.tif]

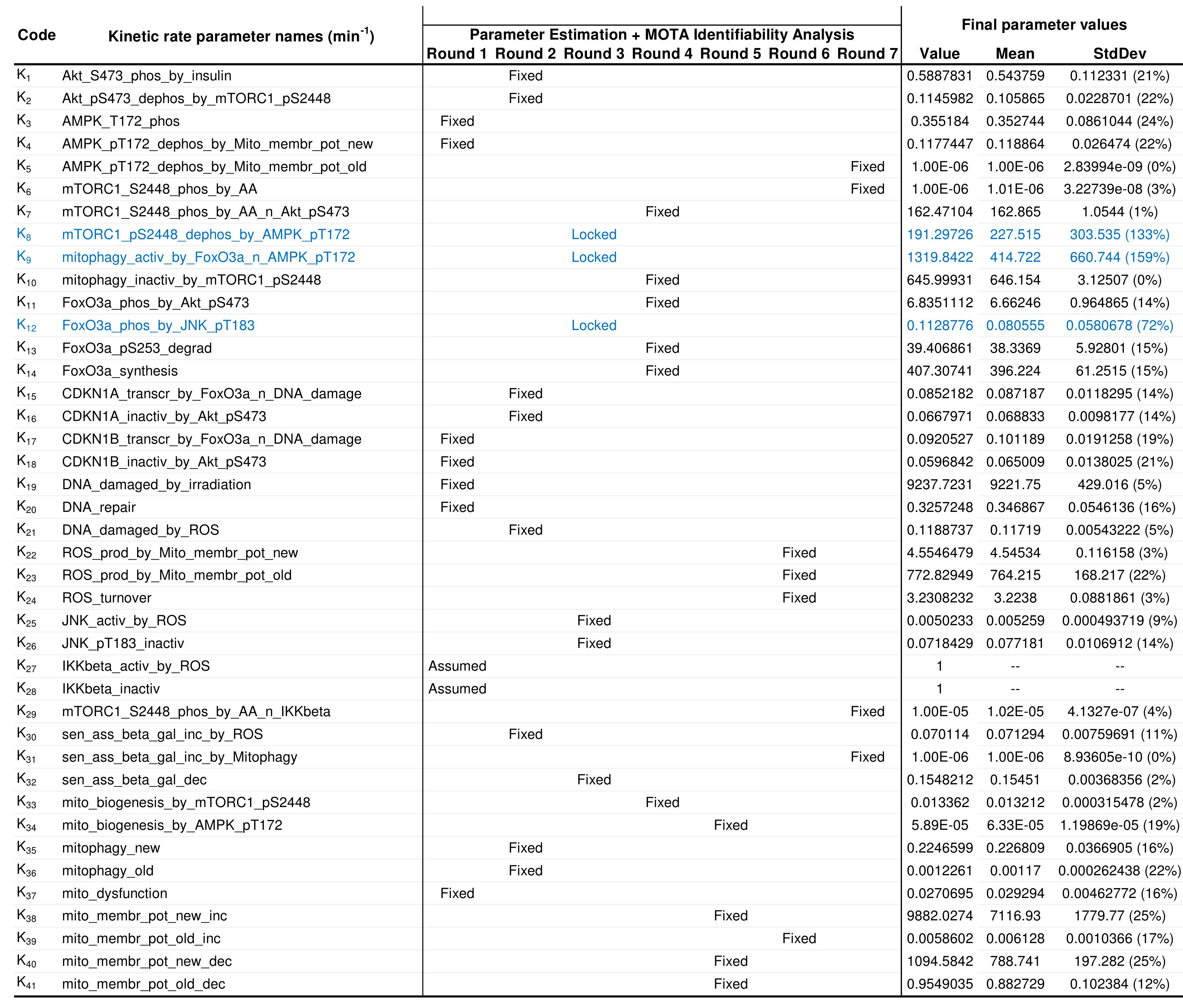

Supplement: Table S4 — Table of the estimated parameters in the model. Up to 7 rounds of alternated parameter estimation and MOTA identifiability analysis were computed in order to progressively determine all the kinetic rate constant parameters. The internal round columns include the following labels: Assumed, Fixed and Locked. A parameter was termed assumed when it was assumed a priori, and therefore was not estimated. The only assumed parameters were related to the IKK-β dynamics. A parameter was termed fixed when it could be estimated and identified based on MOTA analysis within a confidence of variance lower than 25% or a correlation coefficient lower than 0.9. A parameter p was termed locked when it was fixed without being completely identifiable according to MOTA analysis. This was done only when 1) p belonged to a tuple of related parameters and each parameter in this tuple only related to the same parameter tuple (e.g. case for k8, k9), or 2) the other parameters in this tuple were not found to significantly relate with p, creating a one-way correlation (e.g. case for k12, see Figure S8). Since these correlations were local and completely confined to the tuple parameters, they did not affect the other unrelated parameters. The parameters in the tuple were only linearly affected. MOTA correlation matrices and plots are provided in Figures S4, S5, S6, S7, S8, S9, S10, S11, S12, S13, S14, S15, S16, S17, and computed from the best 30% of 20,000 fits (see Materials and Methods for more details). For each parameter, the final value, mean, standard deviation and coefficient of variance are reported. Interestingly, parameter estimation reported an extremely low value for the parameter k5, suggesting a poor ATP production by dysfunctional mitochondria. Among the three modalities of mTORC1 activation (amino acids only, amino acids + insulin, amino acids + IKK-β; parameters k6, k7 and k29, respectively), it was detected that mTORC1 activation was significantly stronger in the presence of in [file pcbi.1003728.s031.tif]

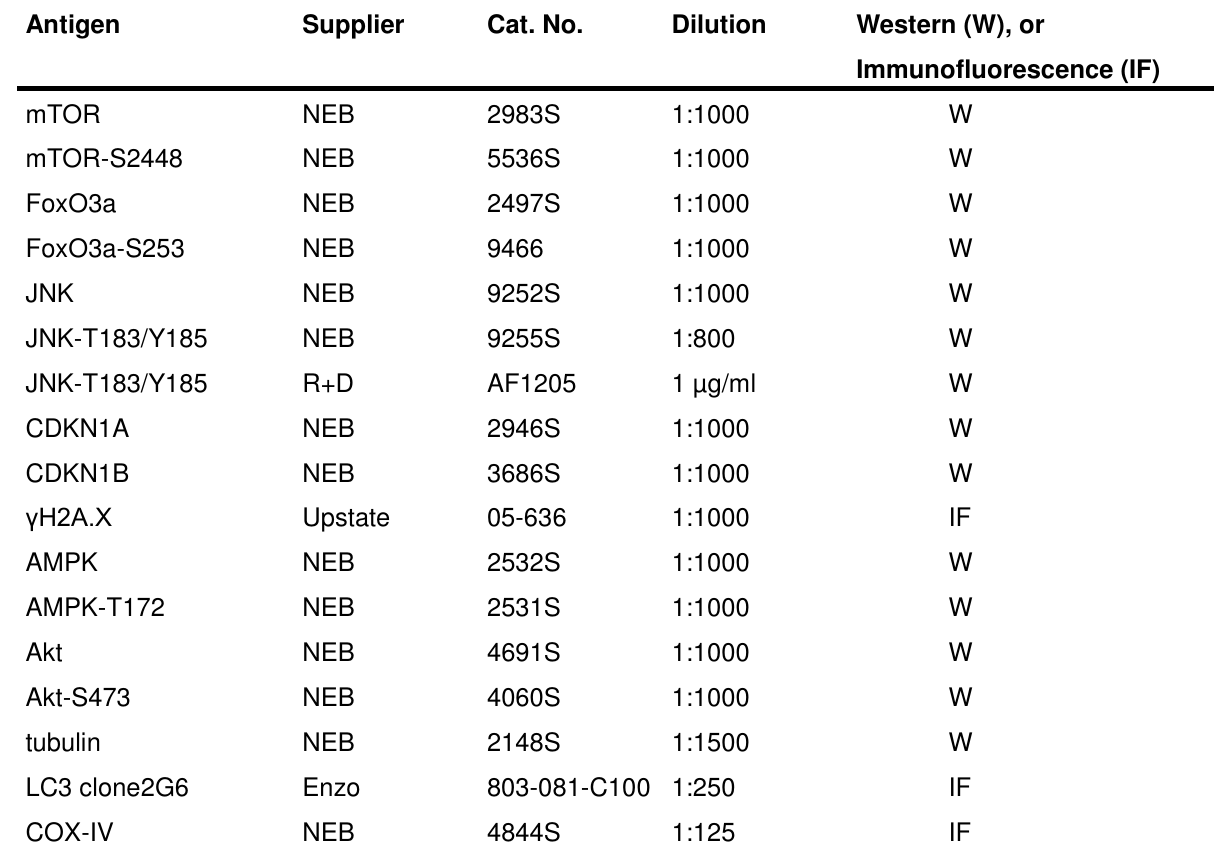

Supplement: Table S5 — List of antibodies. The antibodies used in this study are shown, with their respective catalogue numbers and the dilutions used for the respective staining (western or immunofluorescence). (TIF) [file pcbi.1003728.s032.tif]
