# Supplementary material for: Dynamic Modelling of Pathways to Cellular Senescence Reveals Strategies for Targeted Interventions
Source: PLoS Comput Biol. 2014 Aug 28;10(8):e1003728. doi: 10.1371/journal.pcbi.1003728 (PMC4159174; doi:10.1371/journal.pcbi.1003728)
Supplement: File S1 — File collecting all the supporting tables and figures. All the supporting tables and figures with their corresponding legends are combined into one document in this file. (PDF) [file pcbi.1003728.s025.pdf]

Supporting Information for

Dynamic modelling of pathways to cellular senescence reveals strategies for targeted interventions

Piero Dalle Pezze*[,1,2], Glyn Nelson*[,1,2], Gisela Otten[1,2], Viktor I. Korolchuk[1,2], Thomas B. L. Kirkwood[1,2], Thomas von Zglinicki[§,1,2], Daryl P. Shanley[§,1,2]

[1] Institute for Ageing and Health, Newcastle University, Campus for Ageing and Vitality, Newcastle upon Tyne, NE4 5PL, United Kingdom
[2] Centre for Integrated Systems Biology of Ageing and Nutrition, Institute for Ageing and Health, Newcastle University, Newcastle upon Tyne, United Kingdom

* these authors contributed equally to this work
§ to whom correspondence should be addressed

DOI: 10.1371/journal.pcbi.1003728

Table of Contents

Table S1. *In vitro* data set used for estimating the model parameters.
Table S2. Legend of the model variables.
Table S3. ODEs table of the model.
Table S4. Table of the estimated parameters in the model.
Table S5. List of antibodies.

Figure S1. Measures of mitophagy during stress induced senescence.
Figure S2. Mitochondrial mass data as determined by flow cytometry.
Figure S3. Cell viability.
Figure S4. MOTA identifiability matrix for round 1 of parameter estimation.
Figure S5. Correlation plots for round 1 of parameter estimation, as detected by MOTA identifiability analysis.
Figure S6. MOTA identifiability matrix round 2 of parameter estimation.
Figure S7. Correlation plots for round 2 of parameter estimation, as detected by MOTA identifiability analysis.
Figure S8. MOTA identifiability matrix for round 3 of parameter estimation.
Figure S9. Correlation plots for round 3 of parameter estimation, as detected by MOTA identifiability analysis.
Figure S10. MOTA identifiability matrix for round 4 of parameter estimation.
Figure S11. Correlation plots for round 4 of parameter estimation, as detected by MOTA identifiability analysis.
Figure S12. MOTA identifiability matrix for round 5 of parameter estimation.
Figure S13. Correlation plots for round 5 of parameter estimation, as detected by MOTA identifiability analysis.
Figure S14. MOTA identifiability matrix for round 6 of parameter estimation.
Figure S15. Correlation plots for round 6 of parameter estimation, as detected by MOTA identifiability analysis.
Figure S16. MOTA identifiability matrix for round 7 of parameter estimation.
Figure S17. Correlation plots for round 7 of parameter estimation, as detected by MOTA identifiability analysis.

Figure S18. Principal component analysis for the model at each round of parameter estimation.

Figure S19. Simulated tools for model inhibition or over-activation over time.

Figure S20. Inhibition of ROS and mTOR *in vitro*.

Figure S21. Analysis of the two mitochondrial sub-populations upon ROS-mTOR combined intervention.

Figure S22. Analysis of senescence–associated b-galactosidase staining upon upon ROS-mTOR combined intervention.

Figure S23. Additional readouts upon AMPK, FoxO3a or mitophagy simulated over-activation.

Figure S24. Model stochastic simulation showed increase stochasticity over time.

Movie S1. Mitochondrial fusion and fission in young and senescent fibroblasts over 30 minutes time courses [provided as external file].

Model S1. SBML code for the model [provided as external file].

File S1. File collecting all the supporting tables and figures [this file].

#PottersWheel Data Format 3.0.12

# Authors	Piero Dalle Pezze, Glyn Nelson, et al. Plos Computational Biology, 2014
# Title	Dynamic modelling of pathways to cellular senescence reveals strategies for targeted interventions
#Cells	Human Diploid Fibroblast MRC5
#System	A dynamic model for cellular senescence

	Name	Stimulus	Type	Time points	Values
Input	Irradiation	1	steps	-1;0;0.003472	0;1;0

Time (days)	Stimulus	FoxO3a_pS253_obs	stdCol-FoxO3a_pS253_obs	FoxO3a_total_obs	stdCol-FoxO3a_total_obs	AMPK_pT172_obs	stdCol-AMPK_pT172_obs	mTOR_pS2448_obs	stdCol-mTOR_pS2448_obs
0	1	10	1.5	20	1.5	10	1.5	10	1.5
0.083	1	NaN	NaN	NaN	NaN	NaN	NaN	NaN	NaN
0.2	1	NaN	NaN	NaN	NaN	NaN	NaN	NaN	NaN
1	1	7.6051292173	4.2854609141	39.2316391763	32.2920873504	7.4299286855	3.5125628495	12.6271758718	4.0284026645
2	1	NaN	NaN	NaN	NaN	NaN	NaN	NaN	NaN
3	1	6.5099030713	1.8938069908	27.6423935342	15.4717960137	4.7184252643	3.5017341456	13.4504849183	3.1134084464
4	1	NaN	NaN	NaN	NaN	NaN	NaN	NaN	NaN
5	1	14.8220377899	2.1707759489	14.7437585449	10.4614588874	1.9298583756	0.9276065305	14.9468971258	4.6233757608
6	1	NaN	NaN	NaN	NaN	NaN	NaN	NaN	NaN
7	1	8.3306439282	5.3994079565	22.3318579641	16.4412180036	3.2953463371	2.5929777858	13.7781529455	4.4175193916
9	1	12.5748785742	5.20489726	11.0990480839	13.6019392396	2.4131210564	1.1690012287	13.4408051278	5.912776546
10	1	12.9980273021	7.1452961323	36.3145437733	18.4399902938	5.7935843656	4.4312551186	18.4981	6.0000
11	1	14.5298999598	7.7874044332	9.9389771521	9.5877982779	3.045945903	3.5344772858	11.5216726425	7.2228536382
14	1	13.0403515424	8.4017879515	36.4426737868	19.8820807355	7.1524528652	4.7840091159	5.7465610995	5.9817410567
15	1	NaN	NaN	NaN	NaN	NaN	NaN	NaN	NaN
17	1	9.0220735373	5.6190902021	23.0905637462	12.6815445211	12.4261872904	8.2065719096	4.4766288273	5.4646008679
20	1	NaN	NaN	NaN	NaN	NaN	NaN	NaN	NaN
21	1	9.6231667994	5.7630834736	14.072964518	7.4942588657	15.3186493911	19.1498281858	6.1044537503	6.1281958182

Time (days)	Akt_pS473_obs	stdCol-Akt_pS473_obs	ROS_obs	stdCol-ROS_obs	Mito_Mass_obs	stdCol-Mito_Mass_obs	JNK_pT183_obs	stdCol-JNK_pT183_obs	Mitophagy_obs	stdCol-Mitophagy_obs
0	10	1.5	10	1.5	1	0.2	10	1.5	10	8.6551037228
0.083	NaN	NaN	NaN	NaN	NaN	NaN	NaN	NaN	NaN	NaN
0.2	NaN	NaN	NaN	NaN	NaN	NaN	NaN	NaN	NaN	NaN
1	10.8656954885	2.0741560675	13.8978494624	0.5820063197	1.3927031599	1.0907963537	11.9173008743	2.5252740121	10.4597701149	17.3948775507
2	NaN	NaN	NaN	NaN	NaN	NaN	NaN	NaN	NaN	NaN
3	4.4939013785	1.7887616952	18.9516129032	1.3026432863	2.1918899748	1.6754884483	13.9014963433	1.9808011943	5.7285873192	4.4824733944
4	NaN	NaN	NaN	NaN	NaN	NaN	NaN	NaN	NaN	NaN
5	6.1639178495	2.3586816809	NaN	NaN	NaN	NaN	16.345156975	3.3041347202	NaN	NaN
6	NaN	NaN	NaN	NaN	NaN	NaN	NaN	NaN	NaN	NaN
7	4.3659008784	2.4955525764	22.7822580645	2.3047450262	5.8156255591	6.3921910538	14.9981700389	3.4064833393	9.5977011494	6.7109895127
9	6.9646191923	0.76412538	NaN	NaN	NaN	NaN	NaN	NaN	NaN	NaN
10	10.0629	3.0000	25.9139784946	2.3047450262	11.174251974	9.8792836758	20.2957757996	3.8609924225	11.6796	10.2458
11	8.9201124714	3.9899833929	NaN	NaN	NaN	NaN	NaN	NaN	NaN	NaN
14	7.057594435	3.7150755509	24.6639784946	2.0476113755	9.9659566426	7.2238024751	19.2572896917	5.0466172763	10.5064655172	11.5481074062
15	NaN	NaN	NaN	NaN	NaN	NaN	NaN	NaN	NaN	NaN
17	5.759853554	3.2020032139	18.6693548387	1.8957642974	9.0048148296	7.2834127954	22.080570042	4.4765716601	15.933412604	14.6127695199
20	NaN	NaN	NaN	NaN	NaN	NaN	NaN	NaN	NaN	NaN
21	6.9634711602	1.8693922365	18.4274193548	1.8957642974	11.1573001603	14.8779874883	19.9462264644	5.0202392793	14.0625	13.1679037568

Time (days)	Mito_Membr_Pot_obs	stdCol-Mito_Membr_Pot_obs	CDKN1A_obs	stdCol-CDKN1A_obs	CDKN1B_obs	stdCol-CDKN1B_obs	DNA_damage_gammaH2AX_obs	stdCol-DNA_damage_gammaH2AX_obs	SA_beta_gal_obs	stdCol-SA_beta_gal_obs
0	12.1290598563	4.0327899166	10	1.5	10	1.5	1	0.3333333335	0.81	0.08
0.083	NaN	NaN	NaN	NaN	NaN	NaN	34.1666666665	4.1666666665	NaN	NaN
0.2	NaN	NaN	NaN	NaN	NaN	NaN	NaN	NaN	NaN	NaN
1	10.3697872118	2.5031595001	21.8450436948	3.4503673314	15.2258700866	5.3307058026	26.6666666665	5	NaN	NaN
2	NaN	NaN	NaN	NaN	NaN	NaN	15	1.6666666665	NaN	NaN
3	14.8814849102	4.6600294091	28.5611521279	3.8372035376	39.5729121874	16.146629472	NaN	NaN	NaN	NaN
4	NaN	NaN	NaN	NaN	NaN	NaN	NaN	NaN	3.8000	0.3800
5	NaN	NaN	27.0965349772	3.8046	36.8490320641	13.1449560672	NaN	NaN	NaN	NaN
6	NaN	NaN	NaN	NaN	NaN	NaN	NaN	NaN	NaN	NaN
7	10.6026353957	2.0409958775	23.5683053235	5.0204128957	31.0668025326	12.1737700359	NaN	NaN	NaN	NaN
9	NaN	NaN	23.9833295216	6.3445888278	27.8535836209	12.8111657449	NaN	NaN	7.8300	0.7800
10	5.1365748778	1.8376317107	NaN	NaN	NaN	NaN	8.3333333335	1.3333333335	NaN	NaN
11	NaN	NaN	19.8861322223	4.6742930842	26.3432368713	13.6764548531	NaN	NaN	NaN	NaN
14	9.7638089346	3.962951911	10.85853216	3.3768607811	12.8123582261	13.7521734335	NaN	NaN	NaN	NaN
15	NaN	NaN	NaN	NaN	NaN	NaN	NaN	NaN	8.8500	0.8800
17	5.5670329265	2.1363920097	11.9250575366	2.4583650191	17.5323149705	19.7521859498	NaN	NaN	NaN	NaN
20	NaN	NaN	NaN	NaN	NaN	NaN	11.6666666665	1.6666666665	NaN	NaN
21	4.1905414922	3.525643199	16.5517496161	5.5436326776	14.3918505443	13.3407502665	NaN	NaN	9.0800	0.9000

Table S1. *In vitro* data set used for estimating the model parameters.

For each model observable, the mean and standard deviation (reported as StdCol) of the quantified *in vitro* intensities are reported. The table is formatted for Potterswheel v. 3.0.12.

Parameter Name (Kinetic rate constants)
K1=Akt_S473_phos_by_insulin
K2=Akt_pS473_dephos_by_mTORC1_pS2448
K3=AMPK_T172_phos
K4=AMPK_pT172_dephos_by_Mito_membr_pot_new
K5=AMPK_pT172_dephos_by_Mito_membr_pot_old
K6=mTORC1_S2448_phos_by_AA
K7=mTORC1_S2448_phos_by_AA_n_Akt_pS473
K8=mTORC1_pS2448_dephos_by_AMPK_pT172
K9=mitophagy_activ_by_FoxO3a_n_AMPK_pT172
K10=mitophagy_inactiv_by_mTORC1_pS2448
K11=FoxO3a_phos_by_Akt_pS473
K12=FoxO3a_phos_by_JNK_pT183
K13=FoxO3a_pS253_degrad
K14=FoxO3a_synthesis
K15=CDKN1A_transcr_by_FoxO3a_n_DNA_damage
K16=CDKN1A_inactiv_by_Akt_pS473
K17=CDKN1B_transcr_by_FoxO3a_n_DNA_damage
K18=CDKN1B_inactiv_by_Akt_pS473
K19=DNA_damaged_by_irradiation
K20=DNA_repair
K21=DNA_damaged_by_ROS
K22=ROS_prod_by_Mito_membr_pot_new
K23=ROS_prod_by_Mito_membr_pot_old
K24=ROS_turnover
K25=JNK_activ_by_ROS
K26=JNK_pT183_inactiv
K27=IKKbeta_activ_by_ROS
K28=IKKbeta_inactiv
K29=mTORC1_S2448_phos_by_AA_n_IKKbeta
K30=sen_ass_beta_gal_inc_by_ROS
K31=sen_ass_beta_gal_inc_by_Mitophagy
K32=sen_ass_beta_gal_dec
K33=mito_biogenesis_by_mTORC1_pS2448
K34=mito_biogenesis_by_AMPK_pT172
K35=mitophagy_new
K36=mitophagy_old
K37=mito_dysfunction
K38=mito_membr_pot_new_inc
K39=mito_membr_pot_old_inc
K40=mito_membr_pot_new_dec
K41=mito_membr_pot_old_dec

(Constraints)	λ
CS1 : [K21 / K19 < 1]	100
CS2 : [K4 / K5 > 1]	100
CS3 : [K22 / K23 < 0.01]	100
CS4 : [K6 / K7 < 0.1]	100
CS5 : [K6 / K29 < 0.1]	100
CS6 : [K39 / K38 < 0.3]	100
CS7 : [K36 / K35 < 1]	100
CS8 : [x18 < 1.5]	100
CS9 : [x18 > 0.5]	100

Parameter Name (Concentrations [a.u.])	Value
u1=Insulin (Constant Input)	1
u2=Amino_Acids (Constant Input)	1
u3=Irradiation (5 min Input at time t=0)	1
x1=Akt	10
x2=Akt_pS473	10
x3=AMPK	10
x4=AMPK_pT172	10
x5=mTORC1	10
x6=mTORC1_pS2448	10
x7=Mitophagy	10
x8=FoxO3a	10
x9=FoxO3a_pS253	10
x10=CDKN1A	10
x11=CDKN1B	10
x12=JNK	25
x13=JNK_pT183	10
x14=ROS	10
x15=DNA_damage	1
x16=SA_beta_gal	0.81
x17=IKKbeta	10
x18=Mito_mass_new	1
x19=Mito_mass_old	0
x20=Mito_mass_turnover	25
x21=Mito_membr_pot_new	12.12
x22=Mito_membr_pot_old	0
x23=Nil	0

(Scaling factors)	
s1=scale_Akt_pS473_obs	1
s2=scale_AMPK_pT172_obs	1
s3=scale_mTOR_pS2448_obs	1
s4=scale_Mitophagy_obs	1
s5=scale_FoxO3a_pS253_obs	1
s6=scale_FoxO3a_total_obs	1
s7=scale_CDKN1A_obs	1
s8=scale_CDKN1B_obs	1
s9=scale_JNK_pT183_obs	1
s10=scale_ROS_obs	1
s11=scale_DNA_damage_gammaH2AX_obs	1
s12=scale_SA_beta_gal_obs	1
s13=scale_Mito_Mass_obs	1
s14=scale_Mito_Membr_Pot_obs	1

(Observables [a.u.])	
y1=Akt_pS473_obs	s1*x2
y2=AMPK_pT172_obs	s2*x4
y3=mTOR_pS2448_obs	s3*x6
y4=Mitophagy_obs	s4*x7
y5=FoxO3a_pS253_obs	s5*x9
y6=FoxO3a_total_obs	s6*(x8+x9)
y7=CDKN1A_obs	s7*x10
y8=CDKN1B_obs	s8*x11
y9=JNK_pT183_obs	s9*x13
y10=ROS_obs	s10*x14
y11=DNA_damage_gammaH2AX_obs	s11*x15
y12=SA_beta_gal_obs	s12*x16
y13=Mito_Mass_obs	s13*(x18+x19)
y14=Mito_Membr_Pot_obs	s14*(x21+x22)

(Compartments [a.u.])	
Cell	1

Table S2. Legend of the model variables.
This table defines the unique codes for the model kinetic rate constants (Ki), species (Xi), scaling factors (Si), observables (Yi) and constraints (CSi). The values for the non-estimated parameters are also provided. The lambda term in the block 'Constraints' defines the constraint strength (higher values means harder constraints) as implemented in PottersWheel.

Species	ODE
Akt	dx 1/dt = -k1 *x1 *u1 + k2 *x2 *x6;
Akt_pS473	dx 2/dt = k1 *x1 *u1 - k2 *x2 *x6;
AMPK	dx 3/dt = -k3 *x3 + k4 *x4 *x21 + k5 *x4 *x22;
AMPK_pT172	dx 4/dt = k3 *x3 - k4 *x4 *x21 - k5 *x4 *x22;
mTORC1	dx 5/dt = -k6 *x5 *u2 - k7*x5*u2*x2 + k8 *x6 *x4 - k29*x5*u2*x17;
mTORC1_pS2448	dx 6/dt = k6 *x5 *u2 + k7*x5*u2*x2 - k8 *x6 *x4 + k29*x5*u2*x17;
Mitophagy	dx 7/dt = k9 *x8*x4 - k10 *x7 *x6;
FoxO3a	dx 8/dt = -k11 *x8 *x2 + k12 *x9 *x13 + k14 ;
FoxO3a_pS253	dx 9/dt = k11 *x8 *x2 - k12 *x9 *x13 - k13 *x9 ;
CDKN1A	dx10/dt = k15 *x15*x8 - k16 *x10 *x2;
CDKN1B	dx11/dt = k17 *x15*x8 - k18 *x11 *x2;
JNK	dx12/dt = -k25 *x12 *x14 + k26 *x13 ;
JNK_pT183	dx13/dt = k25 *x12 *x14 - k26 *x13 ;
ROS	dx14/dt = k22 *x21 + k23 *x22 - k24 *x14 ;
DNA_damage	dx15/dt = k19 *u3 + k21 *x14 - k20 *x15 ;
Sen_ass_beta_gal	dx16/dt = k30 *x14 + k31 *x7 - k32 *x16 ;
IKKbeta	dx17/dt = k27 *x14 - k28 *x17 ;
Mito_mass_new	dx18/dt = k33 *x20 *x6 + k34 *x20 *x6 - k35 *x18 *x7 - k37 *x18 *x10;
Mito_mass_old	dx19/dt = -k36 *x19 *x7 + k37 *x18 *x10;
Mito_mass_turnover	dx20/dt = -k33 *x20 *x6 - k34 *x20 *x6 + k35 *x18 *x7 + k36 *x19 *x7;
Mito_membr_pot_new	dx21/dt = k38 *x18 - k40 *x21 ;
Mito_membr_pot_old	dx22/dt = k39 *x19 - k41 *x22 ;
Nil	dx23/dt = k10 *x7 *x6 + k13 *x9 + k16 *x10 *x2 + k18 *x11 *x2 + k20 *x15<br>k24 *x14 + k40 *x21 + k41 *x22 + k28 *x17 ;

Table S3. ODEs table of the model.
The Ordinary Differential Equations (ODEs) for the 23 species defining the model.

Code	Kinetic rate parameter names (min^{-1})	Parameter Estimation + MOTA Identifiability Analysis							Final parameter values		
		Round 1	Round 2	Round 3	Round 4	Round 5	Round 6	Round 7	Value	Mean	StdDev
K_1	Akt_S473_phos_by_insulin		Fixed						0.5887831	0.543759	0.112331 (21%)
K_2	Akt_pS473_dephos_by_mTORC1_pS2448		Fixed						0.1145982	0.105865	0.0228701 (22%)
K_3	AMPK_T172_phos	Fixed							0.355184	0.352744	0.0861044 (24%)
K_4	AMPK_pT172_dephos_by_Mito_membr_pot_new	Fixed							0.1177447	0.118864	0.026474 (22%)
K_5	AMPK_pT172_dephos_by_Mito_membr_pot_old							Fixed	1.00E-06	1.00E-06	2.83994e-09 (0%)
K_6	mTORC1_S2448_phos_by_AA							Fixed	1.00E-06	1.01E-06	3.22739e-08 (3%)
K_7	mTORC1_S2448_phos_by_AA_n_Akt_pS473				Fixed				162.47104	162.865	1.0544 (1%)
K_8	mTORC1_pS2448_dephos_by_AMPK_pT172			Locked					191.29726	227.515	303.535 (133%)
K_9	mitophagy_activ_by_FoxO3a_n_AMPK_pT172			Locked					1319.8422	414.722	660.744 (159%)
K_{10}	mitophagy_inactiv_by_mTORC1_pS2448				Fixed				645.99931	646.154	3.12507 (0%)
K_{11}	FoxO3a_phos_by_Akt_pS473				Fixed				6.8351112	6.66246	0.964865 (14%)
K_{12}	FoxO3a_phos_by_JNK_pT183			Locked					0.1128776	0.080555	0.0580678 (72%)
K_{13}	FoxO3a_pS253_degrad				Fixed				39.406861	38.3369	5.92801 (15%)
K_{14}	FoxO3a_synthesis				Fixed				407.30741	396.224	61.2515 (15%)
K_{15}	CDKN1A_transcr_by_FoxO3a_n_DNA_damage		Fixed						0.0852182	0.087187	0.0118295 (14%)
K_{16}	CDKN1A_inactiv_by_Akt_pS473		Fixed						0.0667971	0.068833	0.0098177 (14%)
K_{17}	CDKN1B_transcr_by_FoxO3a_n_DNA_damage	Fixed							0.0920527	0.101189	0.0191258 (19%)
K_{18}	CDKN1B_inactiv_by_Akt_pS473	Fixed							0.0596842	0.065009	0.0138025 (21%)
K_{19}	DNA_damaged_by_irradiation	Fixed							9237.7231	9221.75	429.016 (5%)
K_{20}	DNA_repair	Fixed							0.3257248	0.346867	0.0546136 (16%)
K_{21}	DNA_damaged_by_ROS		Fixed						0.1188737	0.11719	0.00543222 (5%)
K_{22}	ROS_prod_by_Mito_membr_pot_new						Fixed		4.5546479	4.54534	0.116158 (3%)
K_{23}	ROS_prod_by_Mito_membr_pot_old						Fixed		772.82949	764.215	168.217 (22%)
K_{24}	ROS_turnover						Fixed		3.2308232	3.2238	0.0881861 (3%)
K_{25}	JNK_activ_by_ROS			Fixed					0.0050233	0.005259	0.000493719 (9%)
K_{26}	JNK_pT183_inactiv			Fixed					0.0718429	0.077181	0.0106912 (14%)
K_{27}	IKKbeta_activ_by_ROS	Assumed							1	--	--
K_{28}	IKKbeta_inactiv	Assumed							1	--	--
K_{29}	mTORC1_S2448_phos_by_AA_n_IKKbeta							Fixed	1.00E-05	1.02E-05	4.1327e-07 (4%)
K_{30}	sen_ass_beta_gal_inc_by_ROS		Fixed						0.070114	0.071294	0.00759691 (11%)
K_{31}	sen_ass_beta_gal_inc_by_Mitophagy							Fixed	1.00E-06	1.00E-06	8.93605e-10 (0%)
K_{32}	sen_ass_beta_gal_dec			Fixed					0.1548212	0.15451	0.00368356 (2%)
K_{33}	mito_biogenesis_by_mTORC1_pS2448				Fixed				0.013362	0.013212	0.000315478 (2%)
K_{34}	mito_biogenesis_by_AMPK_pT172					Fixed			5.89E-05	6.33E-05	1.19869e-05 (19%)
K_{35}	mitophagy_new		Fixed						0.2246599	0.226809	0.0366905 (16%)
K_{36}	mitophagy_old		Fixed						0.0012261	0.00117	0.000262438 (22%)
K_{37}	mito_dysfunction	Fixed							0.0270695	0.029294	0.00462772 (16%)
K_{38}	mito_membr_pot_new_inc					Fixed			9882.0274	7116.93	1779.77 (25%)
K_{39}	mito_membr_pot_old_inc						Fixed		0.0058602	0.006128	0.0010366 (17%)
K_{40}	mito_membr_pot_new_dec					Fixed			1094.5842	788.741	197.282 (25%)
K_{41}	mito_membr_pot_old_dec					Fixed			0.9549035	0.882729	0.102384 (12%)

Table S4. Table of the estimated parameters in the model.
Up to 7 rounds of alternated parameter estimation and MOTA identifiability analysis were computed in order to progressively determine all the kinetic rate constant parameters. The internal round columns include the following labels: Assumed, Fixed and Locked. A parameter was termed assumed when it was assumed a priori, and therefore was not estimated. The only assumed parameters were related to the IKK-β dynamics. A parameter was termed fixed when it could be estimated and identified based on MOTA analysis within a confidence of variance lower than 25% or a correlation coefficient lower than 0.9. A parameter *p* was termed locked when it was fixed without being completely identifiable according to MOTA analysis. This was done only when 1) *p* belonged to a tuple of related parameters and each parameter in this tuple only related to the same parameter tuple (e.g. case for k8, k9), or 2) the other parameters in this tuple were not found to significantly relate with p, creating a one-way correlation (e.g. case for k12, see Figure S8). Since these correlations were local and completely confined to the tuple parameters, they did not affect the other unrelated parameters. The parameters in the tuple were only linearly affected. MOTA correlation matrices and plots are provided in Figures S4-S17, and computed from the best 30% of 20,000 fits (see Materials and Methods for more details). For each parameter, the final value, mean, standard deviation and coefficient of variance are reported. Interestingly, parameter estimation reported an extremely low value for the parameter k5, suggesting a poor ATP production by dysfunctional mitochondria. Among the three modalities of mTORC1 activation (amino acids only, amino acids + insulin, amino acids + IKK-β; parameters k6, k7 and k29, respectively), it was detected that mTORC1 activation was significantly stronger in the presence of insulin. Finally, parameter estimation also suggested that the increase in SA-β-gal (see k30, k31) was largely dependent on ROS rather than mitophagy.

Antigen	Supplier	Cat. No.	Dilution	Western (W), or Immunofluorescence (IF)
mTOR	NEB	2983S	1:1000	W
mTOR-S2448	NEB	5536S	1:1000	W
FoxO3a	NEB	2497S	1:1000	W
FoxO3a-S253	NEB	9466	1:1000	W
JNK	NEB	9252S	1:1000	W
JNK-T183/Y185	NEB	9255S	1:800	W
JNK-T183/Y185	R+D	AF1205	1 µg/ml	W
CDKN1A	NEB	2946S	1:1000	W
CDKN1B	NEB	3686S	1:1000	W
γH2A.X	Upstate	05-636	1:1000	IF
AMPK	NEB	2532S	1:1000	W
AMPK-T172	NEB	2531S	1:1000	W
Akt	NEB	4691S	1:1000	W
Akt-S473	NEB	4060S	1:1000	W
tubulin	NEB	2148S	1:1500	W
LC3 clone2G6	Enzo	803-081-C100	1:250	IF
COX-IV	NEB	4844S	1:125	IF

Table S5. List of antibodies.
The antibodies used in this study are shown, with their respective catalogue numbers and the dilutions used for the respective staining (western or immunofluorescence).

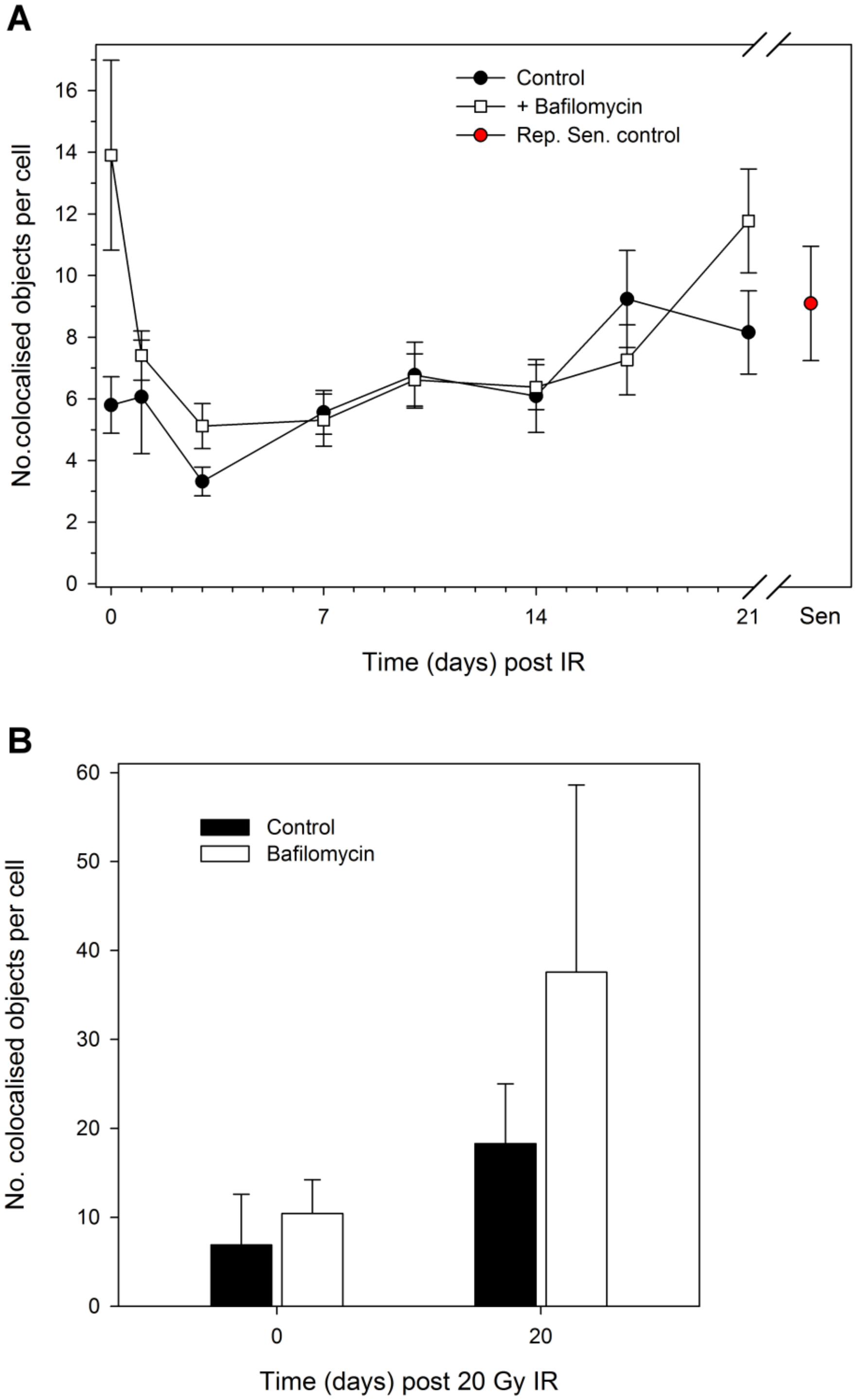

Figure S1. Measures of mitophagy during stress induced senescence.
(A) Cells were fixed at the indicated timepoint with or without 1 hour pre treatment with 400 nM Bafilomycin A, and then stained for LC3 and COX-IV. Number of co-localised objects were determined as described in the Methods. Replicatively senescent MRC5 cells were included as a positive control. (B) Live cell microscopy was performed using co-transduction of GFP and RFP targeted lysosome and mitochdondrial baculovirus constructs respectively at the timepoints shown. Bafilomycin A treatment and analysis were performed as described for LC3-COX-IV stained cells.

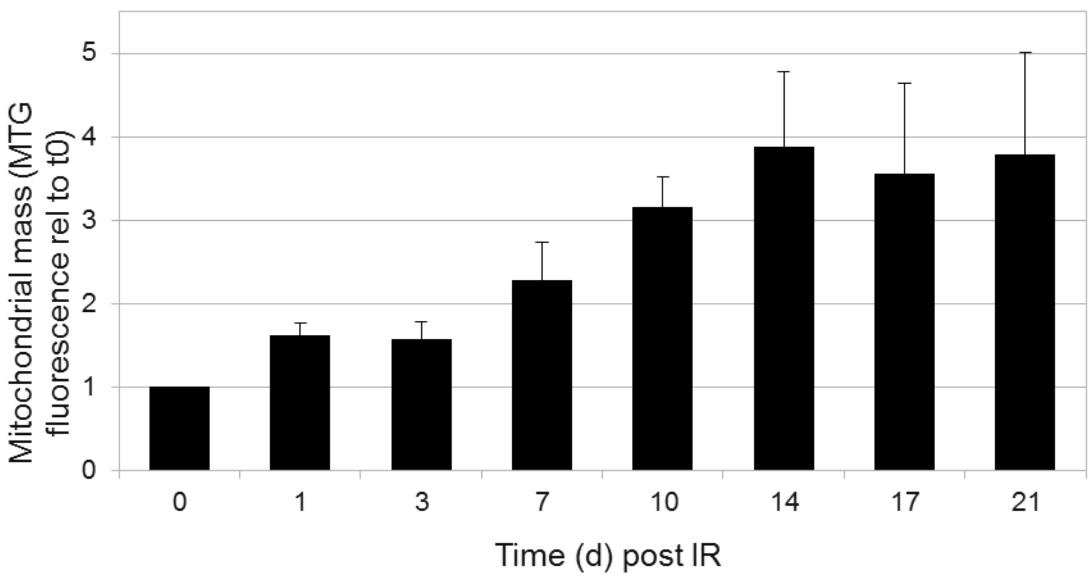

Figure S2. Mitochondrial mass data as determined by flow cytometry.
Cells loaded with Mitotracker Green were analysed by flow cytometry at the time points indicated post 20Gy irradiation. Data represent the median fluorescence intensity from 30,000 cells per time point relative to the 0 day control.

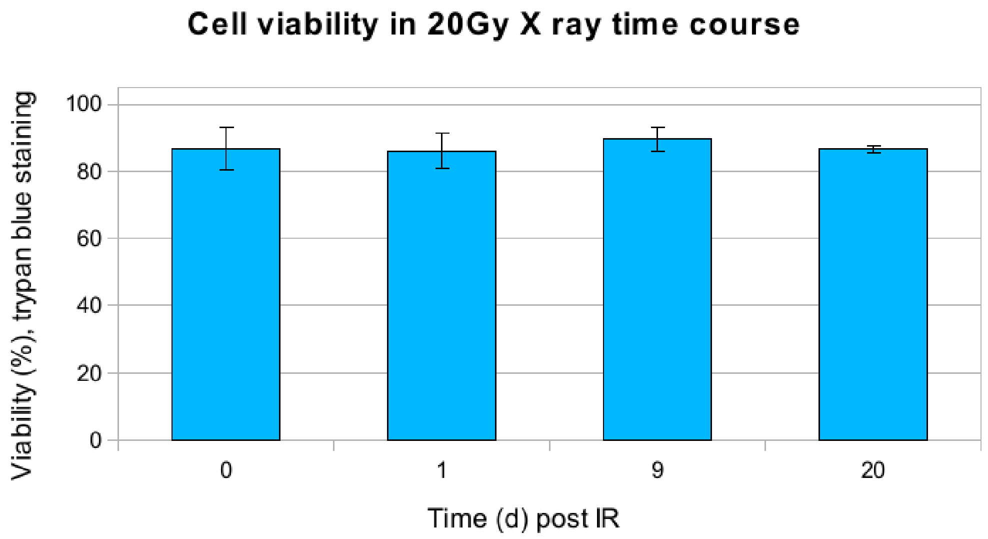

Figure S3. Cell viability.
Cell viability was determined from trypsinised cells (plus spent medium) at the indicated time points post 20Gy irradiation by counting live/dead cells using trypan blue and a haemocytometer. A minimum of 120 cells were recorded per dish per time point, 3 dishes per time point. Data are mean +/- SD. No significant changes in viability were seen over time.

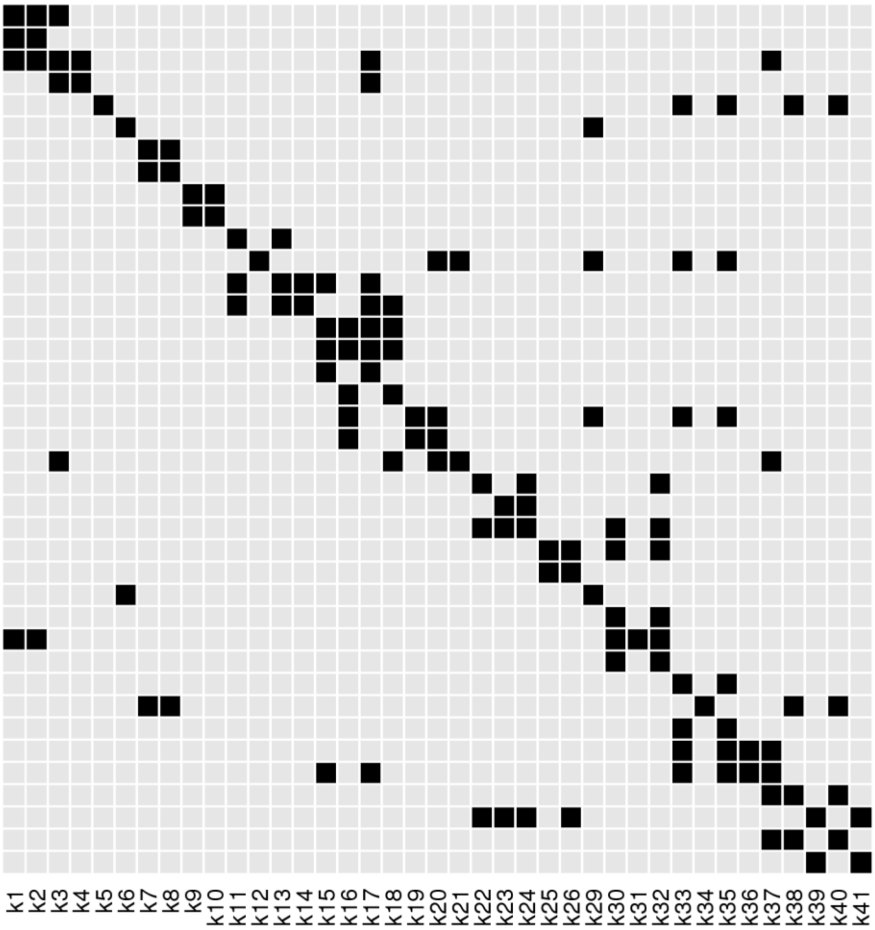

Param	< CC	CV	#	tuple-of-rel-params >
k1	< 1.000	0.881	1	(k1,k2,k3) > *
k2	< 1.000	0.869	1	(k1,k2) > *
k3	< 1.000	0.244	1	(k1,k2,k3,k4,k17,k37) >
k4	< 1.000	0.223	1	(k3,k4,k17) >
k5	< 0.999	6.167	1	(k5,k33,k35,k38,k40) > *
k6	< 0.999	9.354	2	(k6,k29) > **
k7	< 0.999	1.028	2	(k7,k8) > **
k8	< 1.000	3.034	2	(k7,k8) > **
k9	< 1.000	1.120	2	(k9,k10) > **
k10	< 1.000	1.107	2	(k9,k10) > **
k11	< 1.000	1.984	1	(k11,k13) > *
k12	< 0.999	12.967	1	(k12,k20,k21,k29,k33,k35) > *
k13	< 0.999	1.980	1	(k11,k13,k14,k15,k17) > *
k14	< 0.999	1.979	1	(k11,k13,k14,k17,k18) > *
k15	< 1.000	0.511	2	(k15,k16,k17,k18) > **
k16	< 1.000	0.574	2	(k15,k16,k17,k18) > **
k17	< 0.998	0.189	1	(k15,k17) >
k18	< 0.999	0.212	1	(k16,k18) >
k19	< 1.000	0.047	1	(k16,k19,k20,k29,k33,k35) >
k20	< 1.000	0.157	1	(k16,k19,k20) >
k21	< 0.999	0.266	1	(k3,k18,k20,k21,k37) > *
k22	< 1.000	0.680	1	(k22,k24,k32) > *
k23	< 0.998	1.324	1	(k23,k24) > *
k24	< 0.999	0.803	1	(k22,k23,k24,k30,k32) > *
k25	< 1.000	6.369	1	(k25,k26,k30,k32) > *
k26	< 1.000	7.049	1	(k25,k26) > *
k29	< 0.999	3.464	2	(k6,k29) > **
k30	< 0.999	4.382	2	(k30,k32) > **
k31	< 0.999	12.772	1	(k1,k2,k30,k31,k32) > *
k32	< 0.999	8.625	2	(k30,k32) > **
k33	< 1.000	0.422	2	(k33,k35) > **
k34	< 0.999	2.430	1	(k7,k8,k34,k38,k40) > *
k35	< 1.000	0.450	2	(k33,k35) > **
k36	< 1.000	0.480	1	(k33,k35,k36,k37) > *
k37	< 1.000	0.158	1	(k15,k17,k33,k35,k36,k37) >
k38	< 1.000	1.134	2	(k37,k38,k40) > **
k39	< 0.999	0.835	1	(k22,k23,k24,k26,k39,k41) > *
k40	< 1.000	1.125	2	(k37,k38,k40) > **
k41	< 0.999	8.633	1	(k39,k41) > *

Figure S4. MOTA identifiability analysis matrix for round 1.
In this round the kinetic rate constants k3, k4, k17, k18, k19, k20, and k37 were fixed since they were identifiable using MOTA identifiability analysis. * : Correlation Coefficient (CC) > 0.9 and Coefficient of Variation (CV) > 0.25; ** : Correlation Coefficient (CC) > 0.9, Coefficient of Variation (CV) > 0.25 and number of tuples showing this correlation (#) > 1. Format: ParameterCode < CC CV # (“Tuple of related parameters”)>.

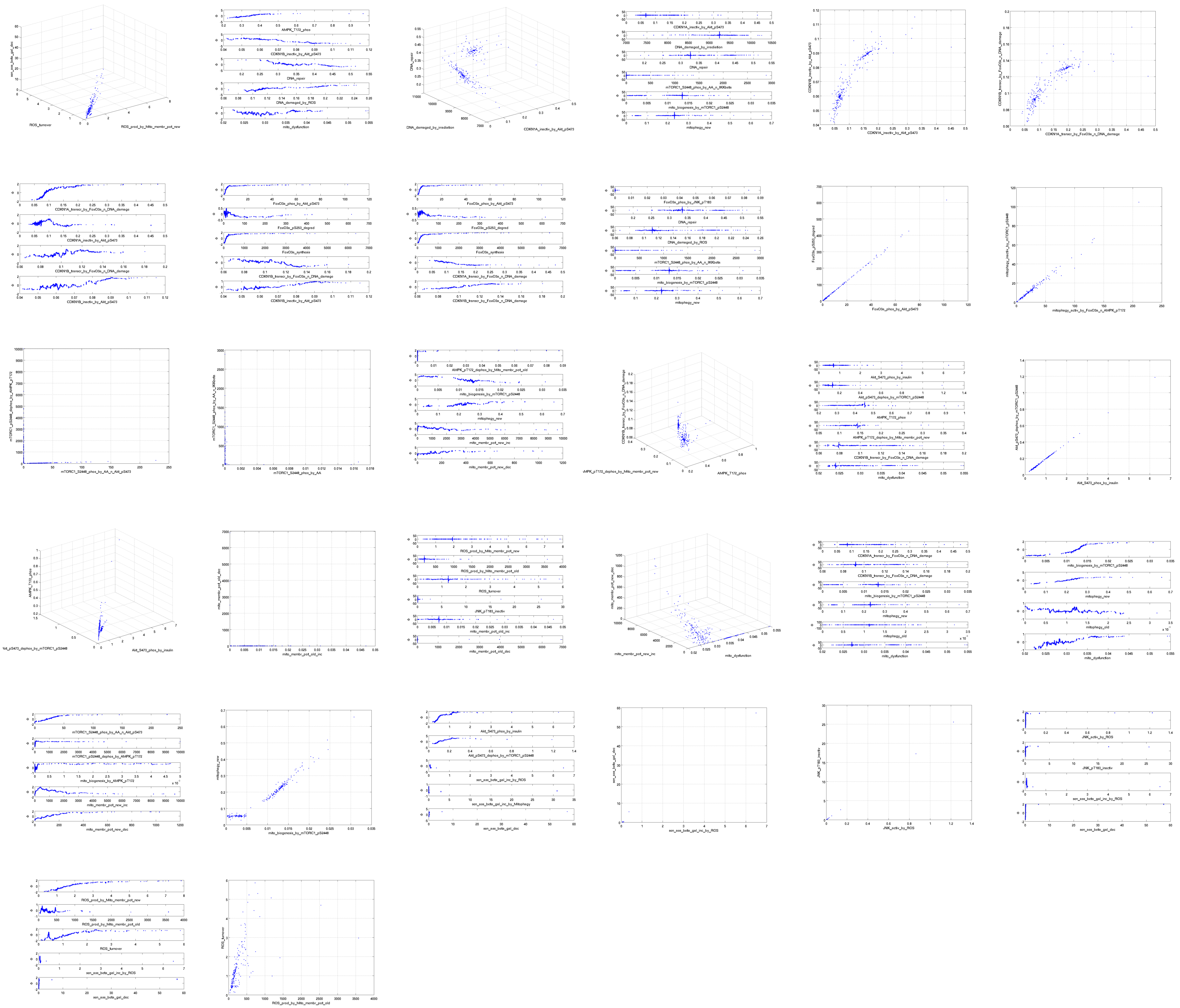

Figure S5. Correlation plots for the round 1 of parameter estimation, as detected by MOTA identifiability analysis.
Plots for the tuples of related parameters reported in the MOTA identifiability matrix in Figure S4.

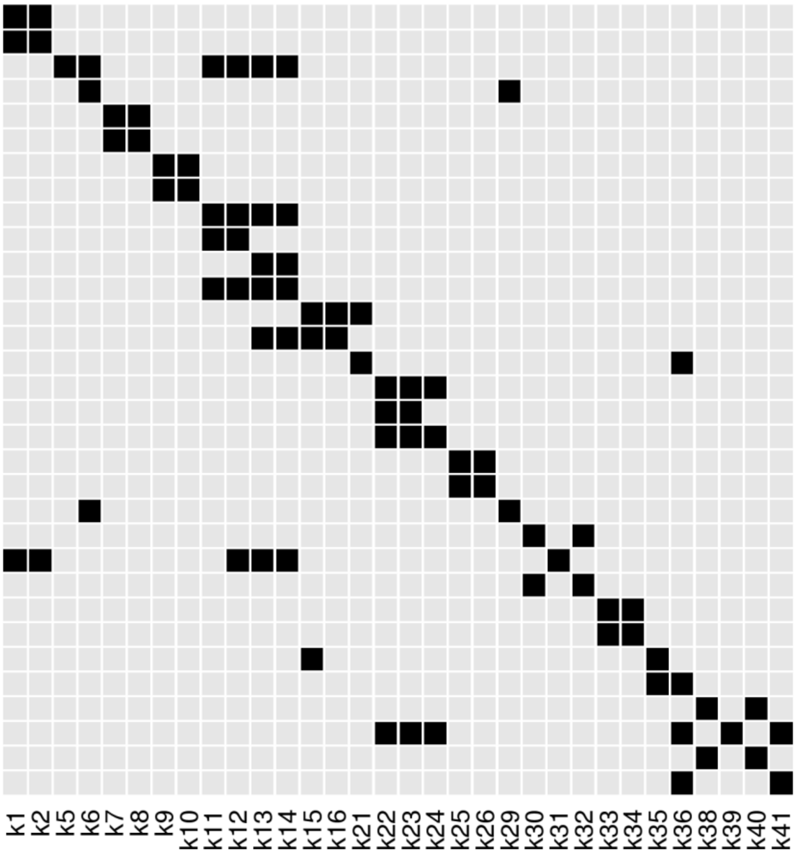

Param	<	CC	CV	#	tuple-of-rel-params	>	
k1	<	1.000	0.207	2	(k1,k2)	>	
k2	<	1.000	0.216	2	(k1,k2)	>	
k5	<	0.999	10.690	1	(k5,k6,k11,k12,k13,k14)	>	*
k6	<	1.000	5.018	2	(k6,k29)	>	**
k7	<	1.000	3.039	2	(k7,k8)	>	**
k8	<	1.000	3.034	2	(k7,k8)	>	**
k9	<	1.000	2.073	2	(k9,k10)	>	**
k10	<	1.000	2.067	2	(k9,k10)	>	**
k11	<	1.000	1.543	2	(k11,k12,k13,k14)	>	**
k12	<	1.000	1.560	1	(k11,k12)	>	*
k13	<	1.000	1.685	1	(k13,k14)	>	*
k14	<	1.000	1.684	2	(k11,k12,k13,k14)	>	**
k15	<	1.000	0.136	1	(k15,k16,k21)	>	
k16	<	0.999	0.143	1	(k13,k14,k15,k16)	>	
k21	<	0.997	0.046	1	(k21,k36)	>	
k22	<	1.000	0.888	2	(k22,k23,k24)	>	**
k23	<	1.000	0.849	1	(k22,k23)	>	*
k24	<	1.000	0.891	2	(k22,k23,k24)	>	**
k25	<	1.000	1.441	2	(k25,k26)	>	**
k26	<	1.000	1.439	2	(k25,k26)	>	**
k29	<	1.000	4.940	2	(k6,k29)	>	**
k30	<	0.996	0.107	2	(k30,k32)	>	
k31	<	0.999	18.970	1	(k1,k2,k12,k13,k14,k31)	>	*
k32	<	0.996	0.608	2	(k30,k32)	>	**
k33	<	0.996	0.343	2	(k33,k34)	>	**
k34	<	0.996	1.004	2	(k33,k34)	>	**
k35	<	0.996	0.162	1	(k15,k35)	>	
k36	<	0.996	0.224	1	(k35,k36)	>	
k38	<	1.000	0.588	2	(k38,k40)	>	**
k39	<	0.999	4.530	1	(k22,k23,k24,k36,k39,k41)	>	*
k40	<	1.000	0.592	2	(k38,k40)	>	**
k41	<	0.996	3.608	1	(k36,k41)	>	*

Figure S6. MOTA identifiability matrix for the round 2 of parameter estimation.
In this round the kinetic rate constants k1, k2, k15, k16, k21, k30, k35, and k36 were fixed since they were identifiable using MOTA identifiability analysis.

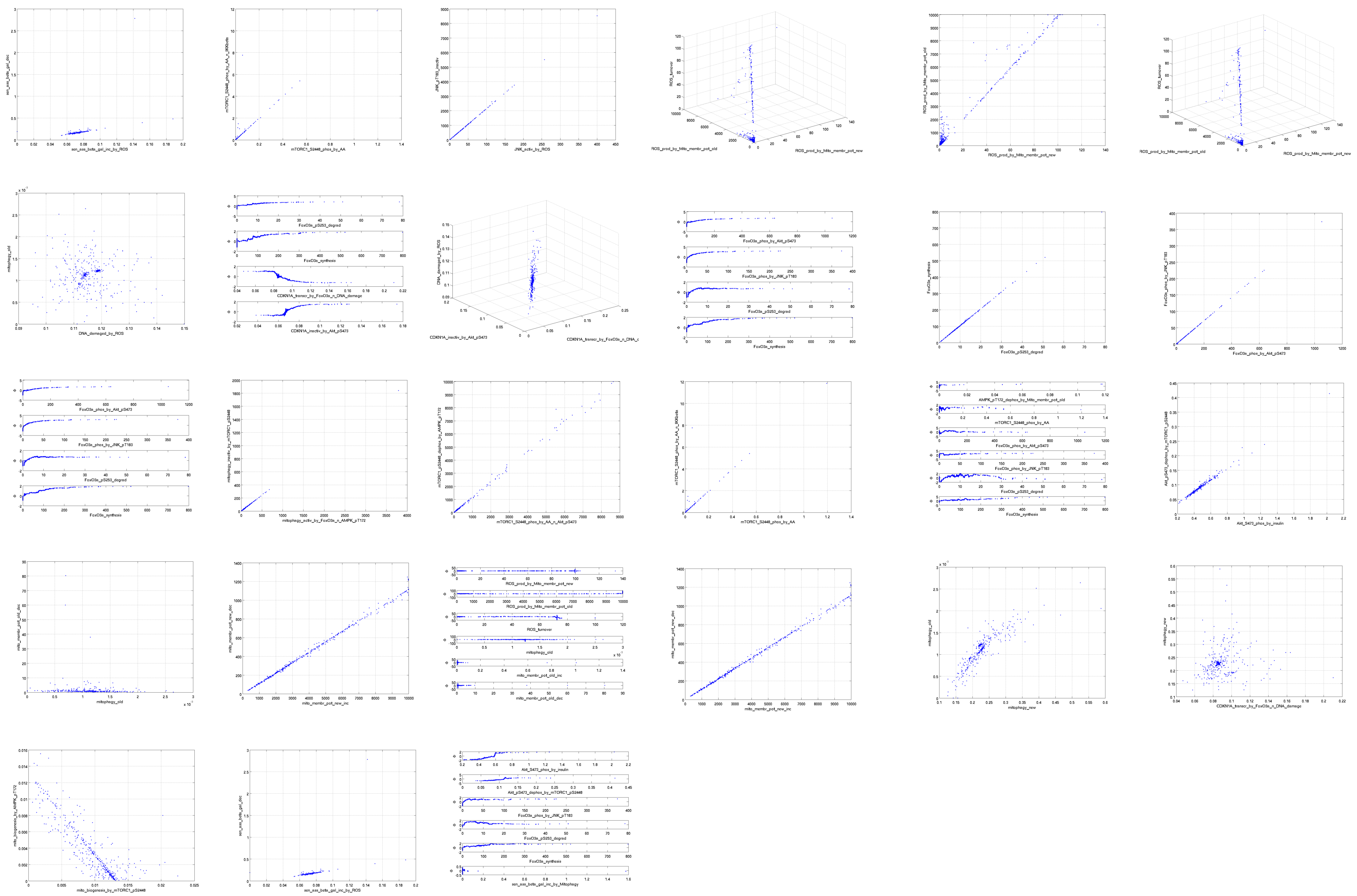

Figure S7. Correlation plots for the round 2 of parameter estimation, as detected by MOTA identifiability analysis.
Plots for the tuples of related parameters reported in the MOTA identifiability matrix in Figure S6.

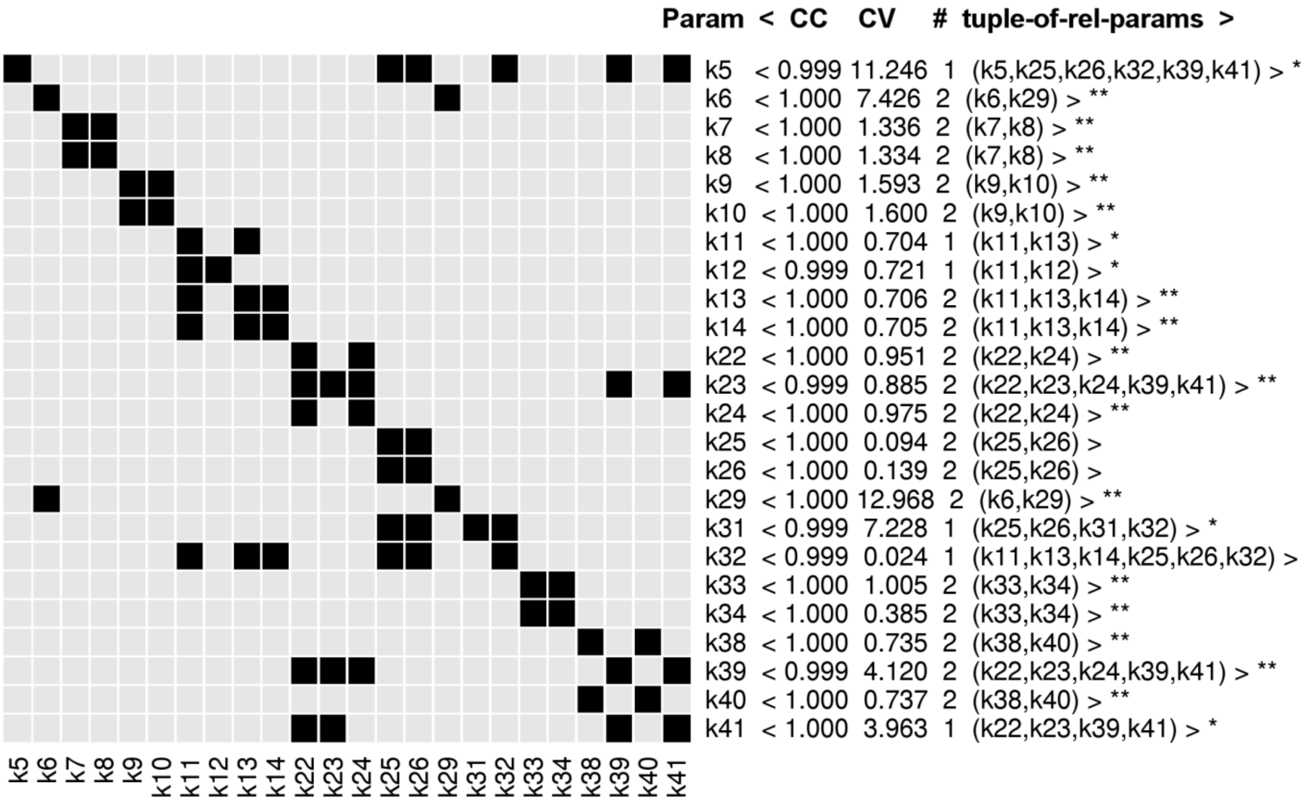

Figure S8. MOTA identifiability matrix for the round 3 of parameter estimation.
In this round the kinetic rate constants k25, k26, and k32 were fixed since they were identifiable using MOTA identifiability analysis. The parameters k8, k9, and k12 were locked as reported in Table S4. The parameters k8 and k9 were part of two couples of related parameters. The former k8 with k7, the latter k9 with k10. All these two couples only related internally with themselves and therefore formed two locally defined correlations. The parameter k12 only related with k11, although k11 was not dependent on k12. Hence, this also formed a locally defined correlation.

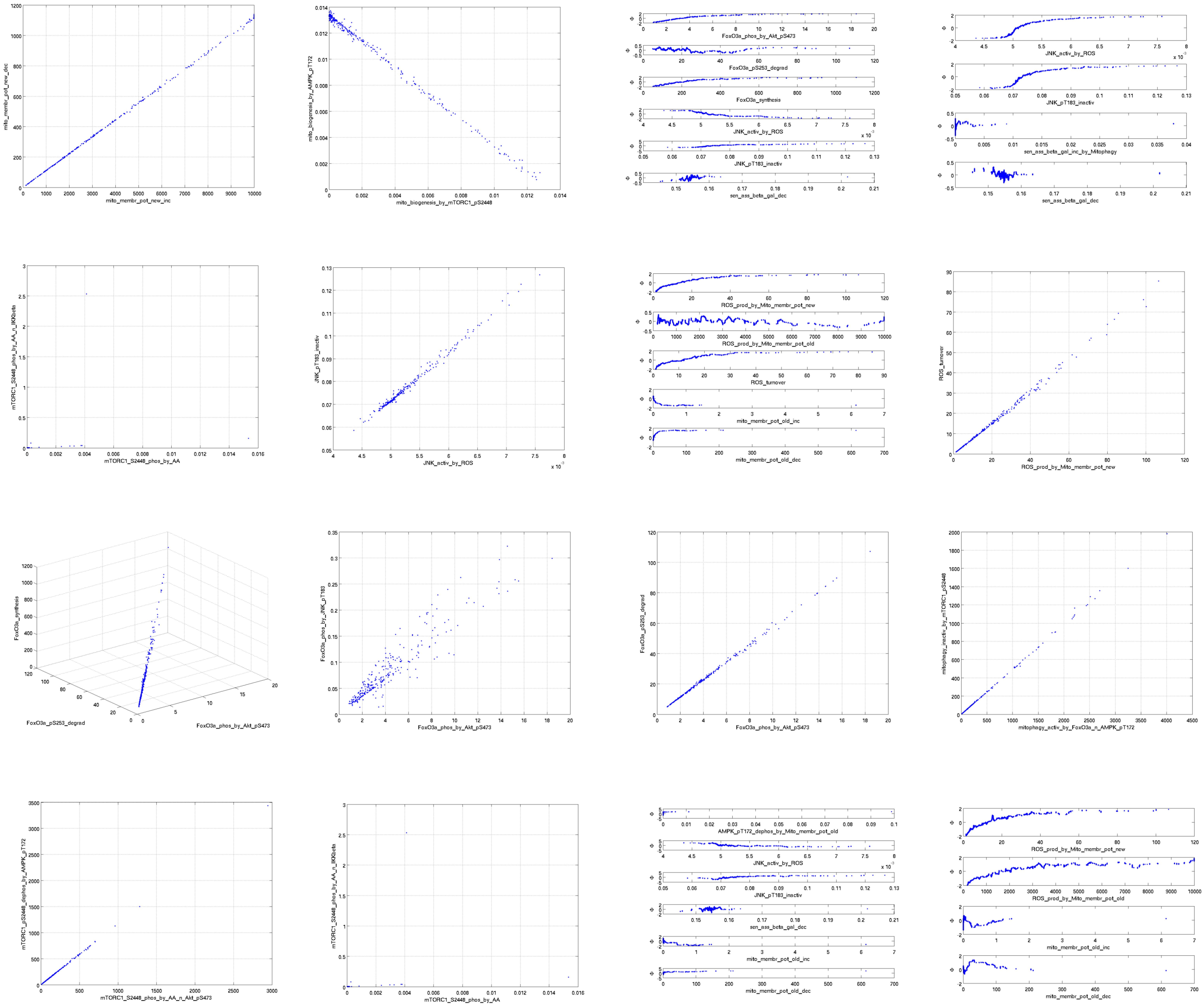

Figure S9. Correlation plots for the round 3 of parameter estimation, as detected by MOTA identifiability analysis.
Plots for the tuples of related parameters reported in the MOTA identifiability matrix in Figure S8.

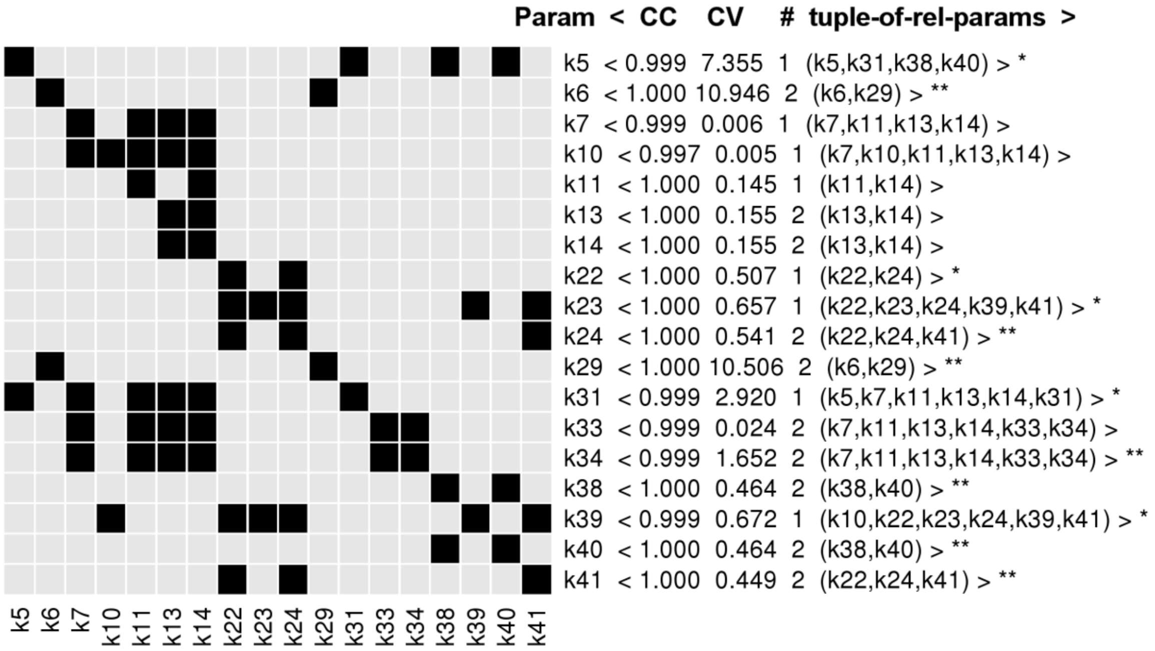

Figure S10. MOTA identifiability matrix for the round 4 of parameter estimation.
In this round the kinetic rate constants k7, k10, k11, k13, k14, and k33 were fixed since they were identifiable using MOTA identifiability analysis.

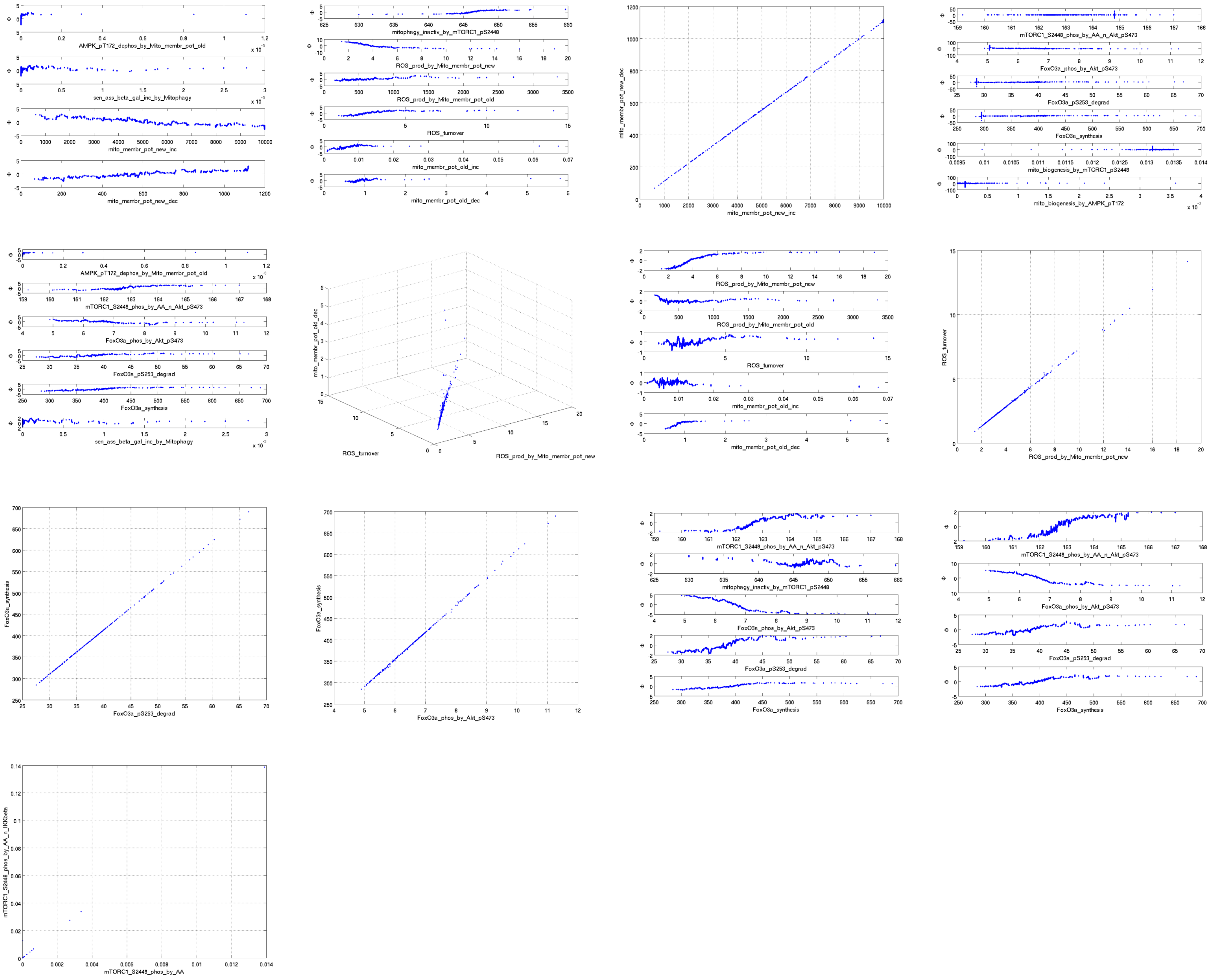

Figure S11. Correlation plots for the round 4 of parameter estimation, as detected by MOTA identifiability analysis. Plots for the tuples of related parameters reported in the MOTA identifiability matrix in Figure S10.

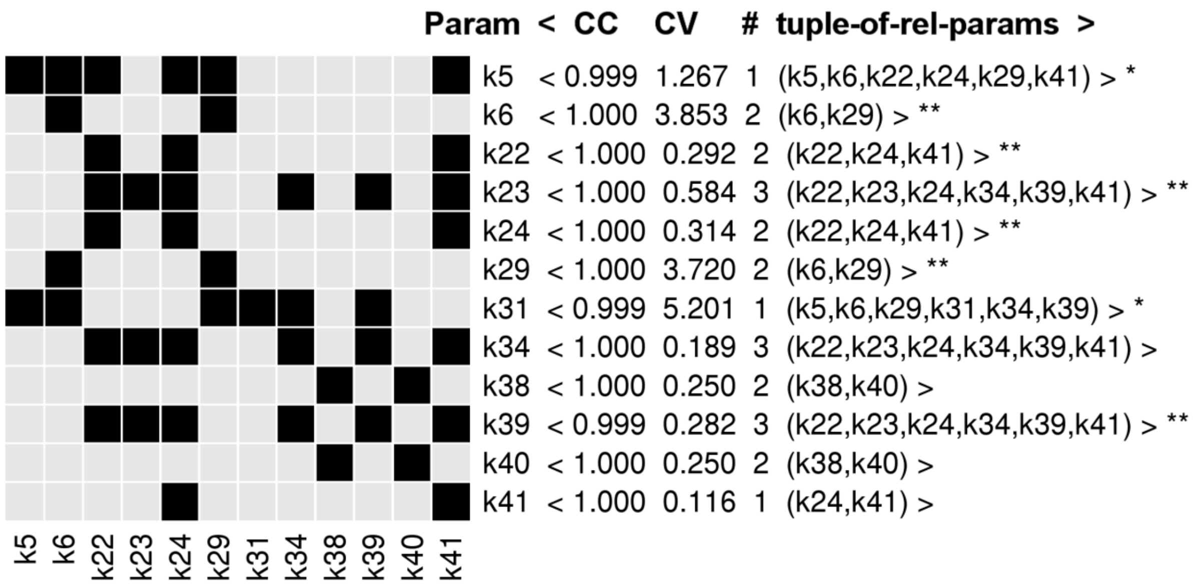

Figure S12. MOTA identifiability matrix for the round 5 of parameter estimation.
In this round the kinetic rate constants k34, k38, k40, and k41 were fixed since they were identifiable using MOTA identifiability analysis.

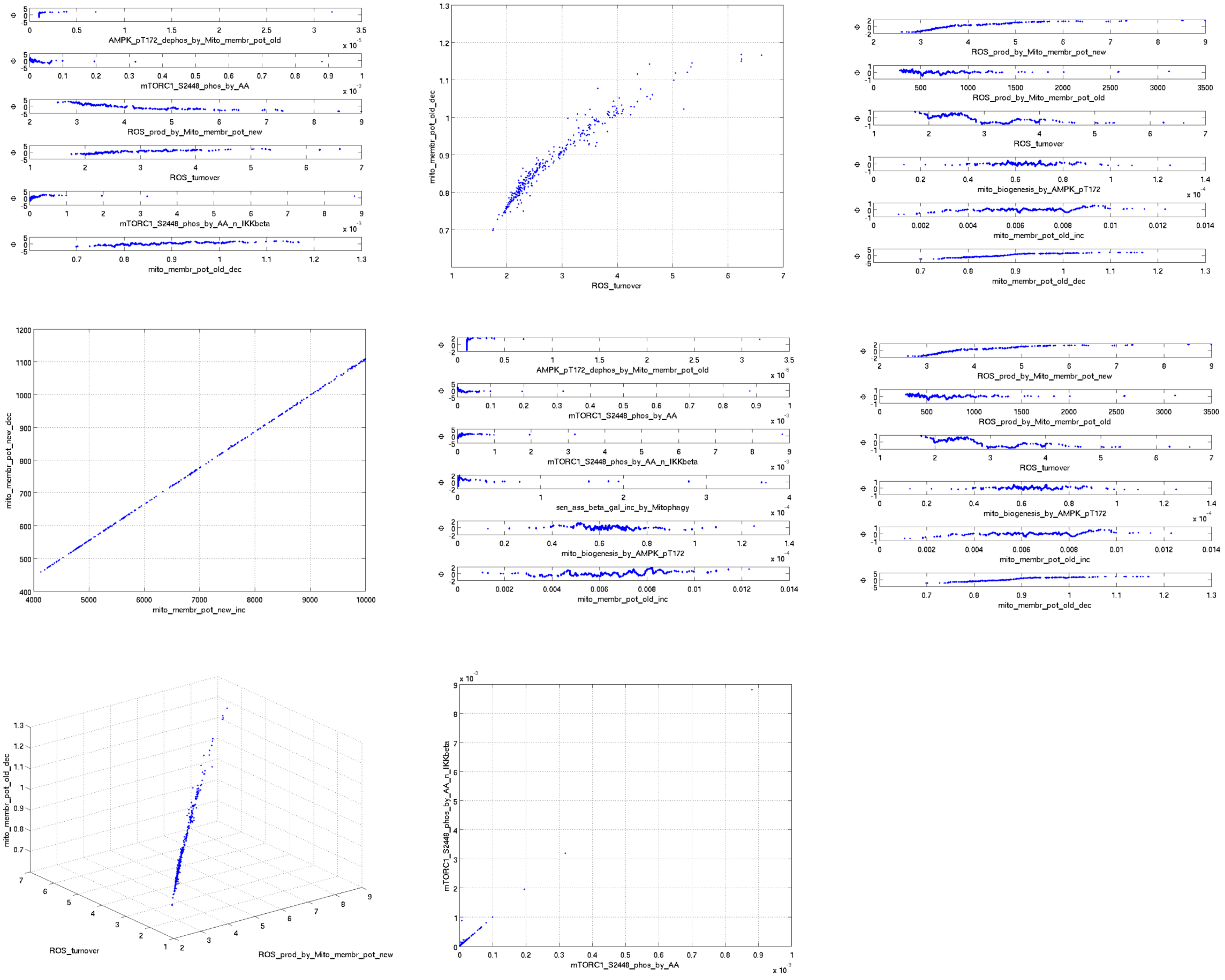

Figure S13. Correlation plots for the round 5 of parameter estimation, as detected by MOTA identifiability analysis. Plots for the tuples of related parameters reported in the MOTA identifiability matrix in Figure S12.

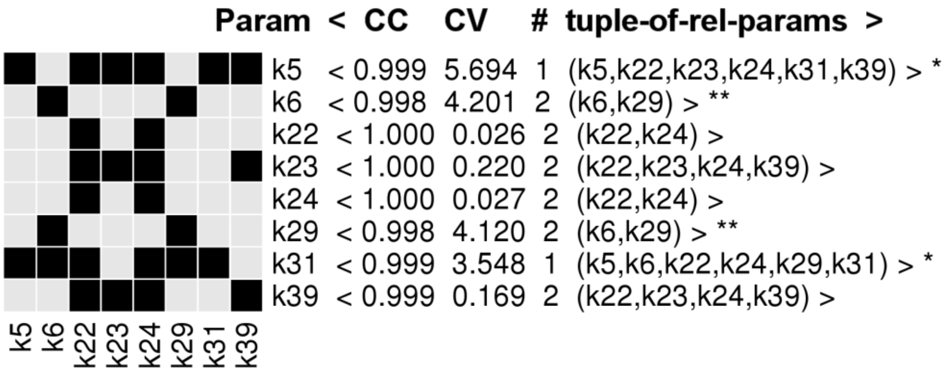

Figure S14. MOTA identifiability matrix for the round 6 of parameter estimation.
In this round the kinetic rate constants k22, k23, k24, and k39 were fixed since they were identifiable using MOTA identifiability analysis.

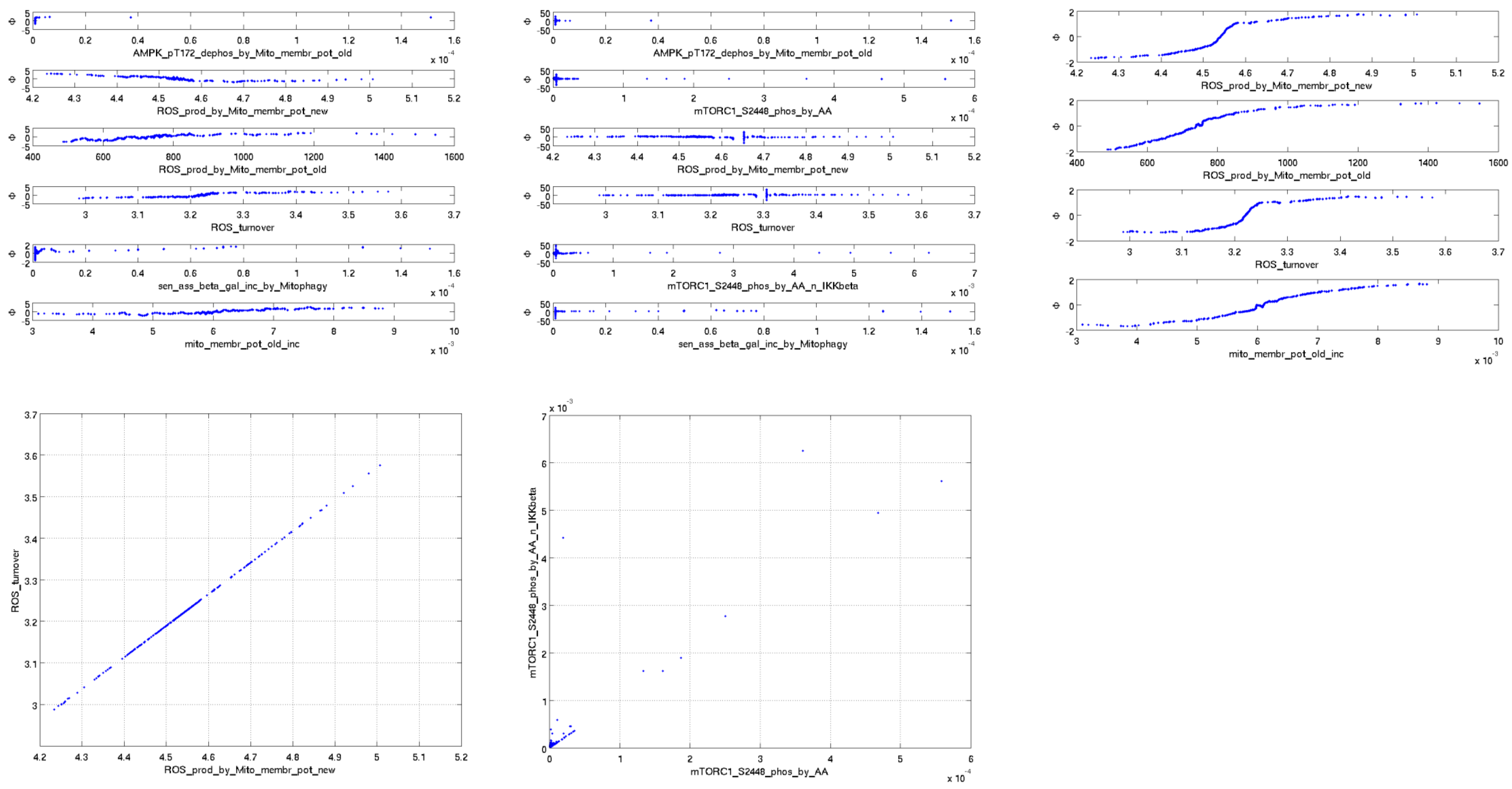

Figure S15. Correlation plots for the round 6 of parameter estimation, as detected by MOTA identifiability analysis. Plots for the tuples of related parameters reported in the MOTA identifiability matrix in Figure S14.

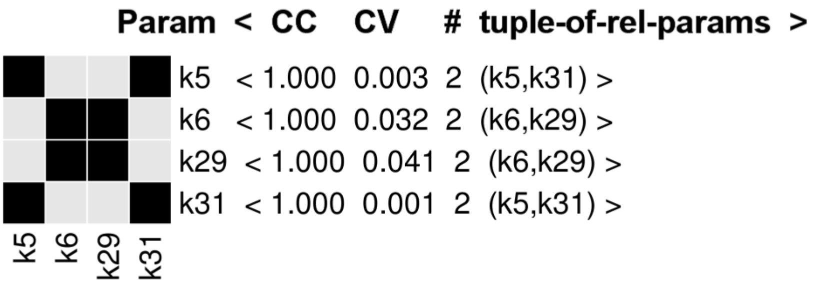

Figure S16. MOTA identifiability matrix for the round 7 of parameter estimation.
In this round the kinetic rate constants k5, k6, k29, and k31 were fixed since they were identifiable using MOTA identifiability analysis.

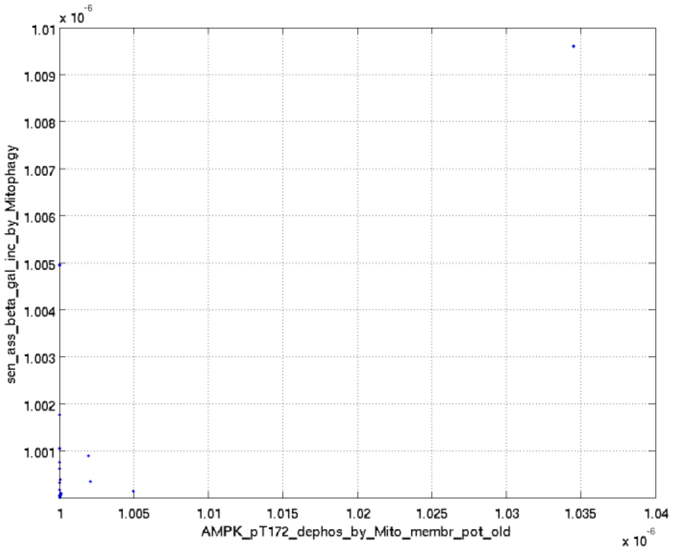

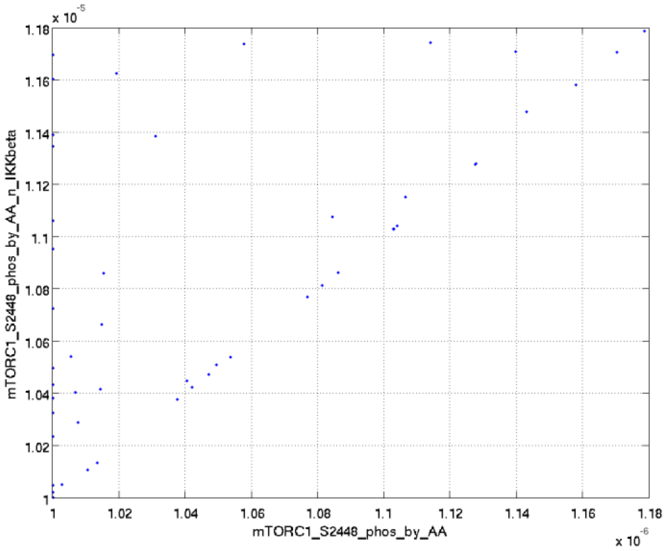

Figure S17. Correlation plots for the round 7 of parameter estimation, as detected by MOTA identifiability analysis.

Plots for the tuples of related parameters reported in the MOTA identifiability matrix in Figure S16.

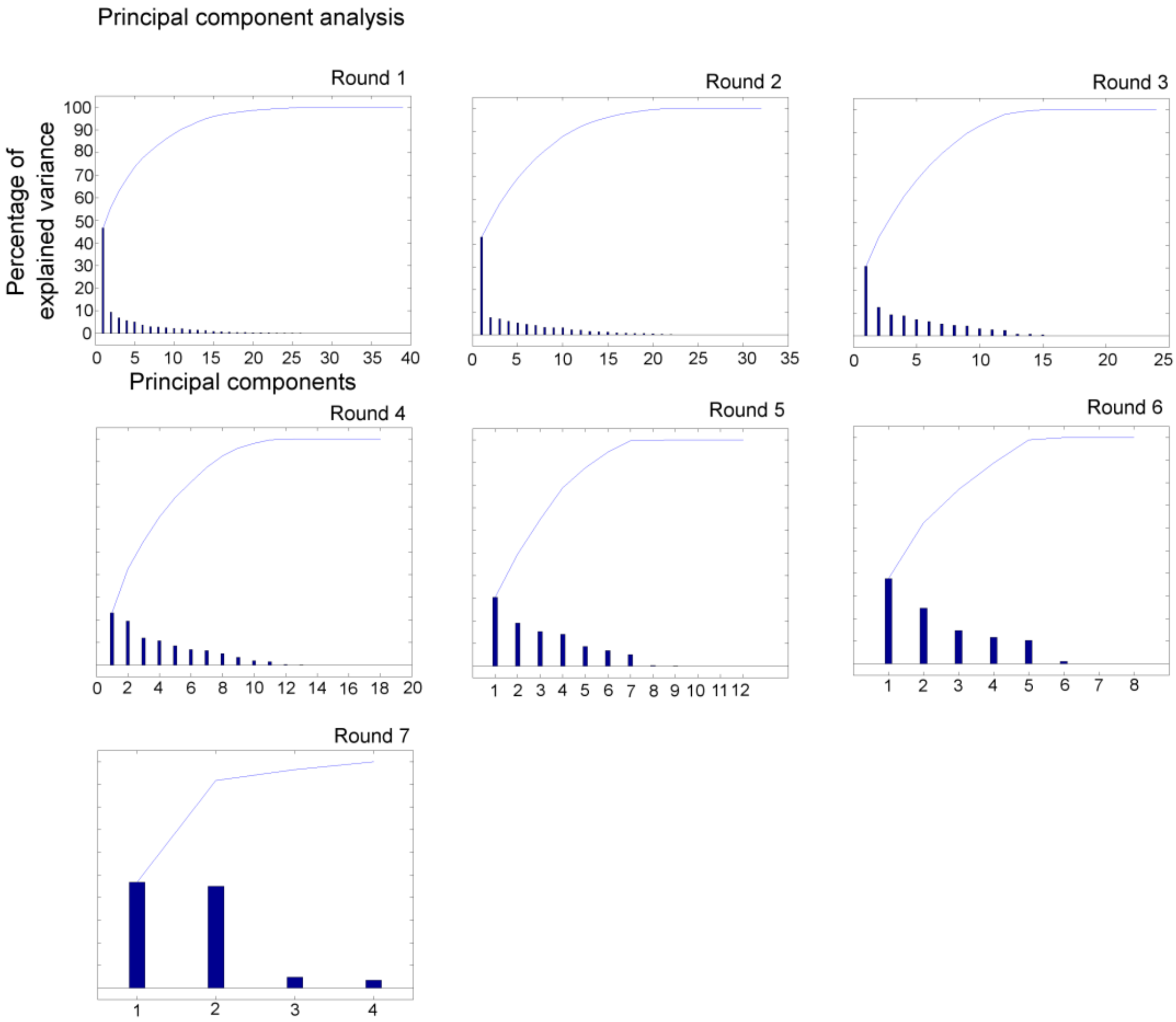

Figure S18. Principal component analysis for the model at each round of parameter estimation. To further investigate the source of variability, principal component analysis (PCA) of the model was computed at each round of parameter estimation. Interestingly, this analysis indicated that only about half of the estimated parameters did not significantly contribute to the overall variance for each round. This suggested that a group of parameters could have been potentially identified at each round.

A

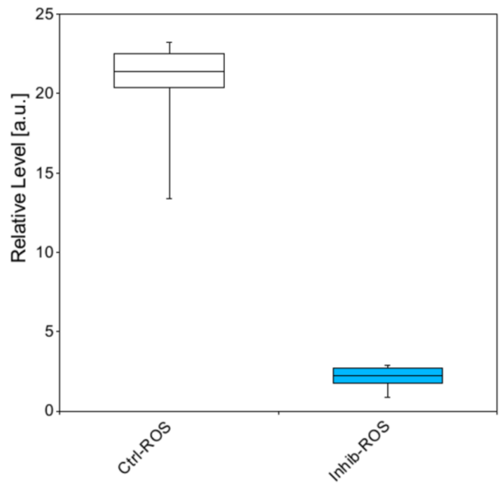

B

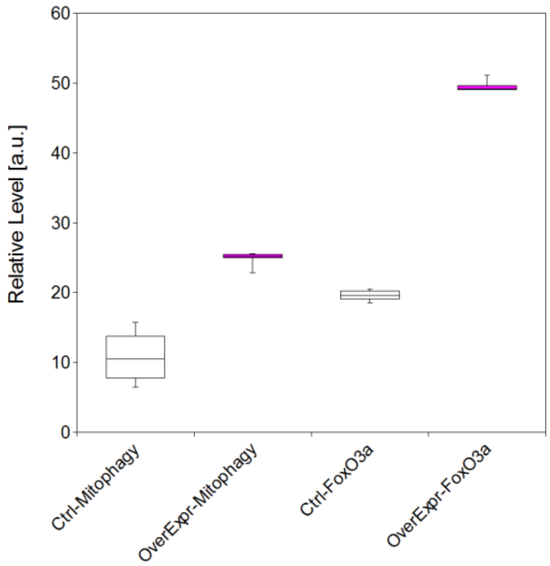

Figure S19. Simulated tools for model inhibition or over-activation over time.
(A) To inhibit ROS over time, a simulated ROS inhibitor species was added to the model. This new species reduced ROS levels and acted as an *in vitro* ROS scavenger. The abundance for this species was estimated in order to achieve ROS inhibition to 10% (blue) as compared to the control (white) throughout the time course. (B) In analogy, two new species were created for over-activating mitophagy and FoxO3a, respectively. The abundance for these two species were estimated to achieve a mitophagy or FoxO3a over-activation of 150% (magenta) as compared to the corresponding control (white) throughout the time course. Each boxplot represents the median and two quartiles, whereas the bars indicate the minimum and the maximum values, as estimated from day 1 to day 21.

A

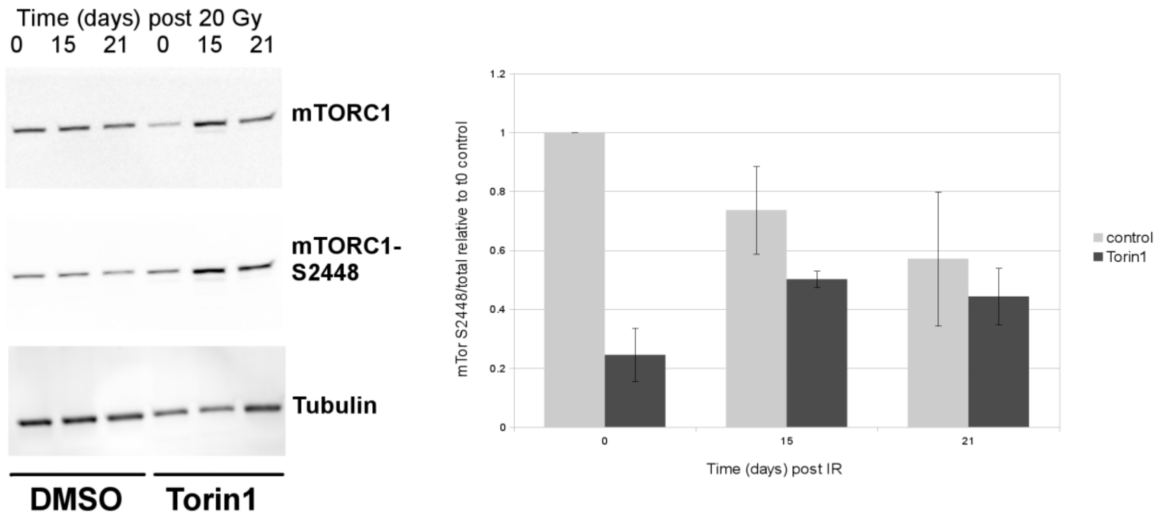

B

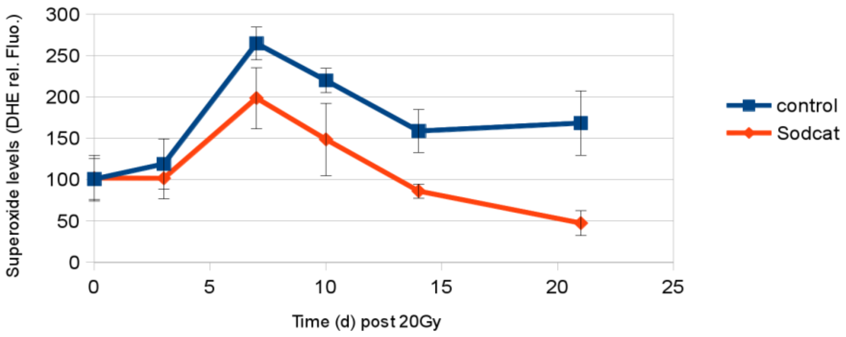

Figure S20. Inhibition of ROS and mTOR *in vitro*.
Torin and ROS inhibition efficacy. (A) Cells were irradiated with 20Gy X irradiation and then treated with Torin1 or DMSO as described in Methods. Lysates were probed with total mTORC1 and mTORC1-S2448 antibodies. Band intensities were quantified relative to Tubulin loading control and plotted as mean +/- SD ratios of mTORC1-S2448 to total mTORC1 (3 repeats). (B) Cells were irradiated with 20Gy X irradiation and then treated with SOD and catalase as described in Methods. Cells were stained with DHE to measure intracellular superoxide levels by flow cytometry. Time course data over 21 days are plotted (n=3).

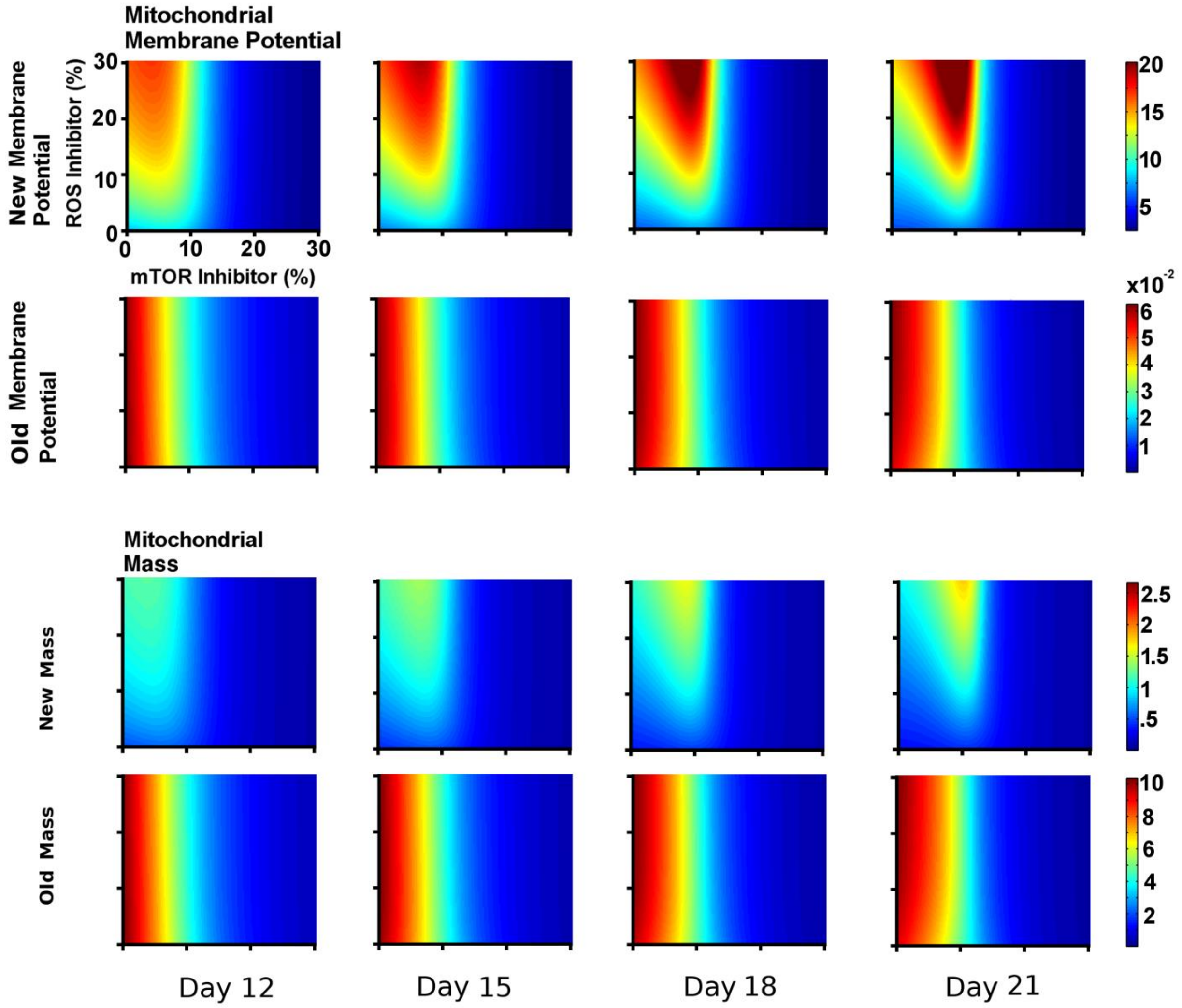

Figure S21. Analysis of the two mitochondrial sub-populations upon ROS-mTOR combined intervention. The internal states for the new and old mitochondrial sub-populations were also investigated upon combined ROS-mTOR simulated intervention. Regarding the mitochondrial membrane potential, the global effect of these interventions mainly resulted from changes in the sub-population of new mitochondria, which showed a strong synergistic response at particular levels of mTOR- ROS inhibition. The effect upon old mitochondrial ψm was almost entirely dependent upon mTOR inhibition, but still changed their potential by an insignificant amount compared to the new mitochondrial population. Concerning the mitochondrial mass, the perturbation of combined ROS-mTOR acted predominantly on the young population, although these changes were largely hidden in the overall population (Figure 4A) due to the larger proportion being comprised of the old population. The point (0, 0) indicates the control (no inhibition).

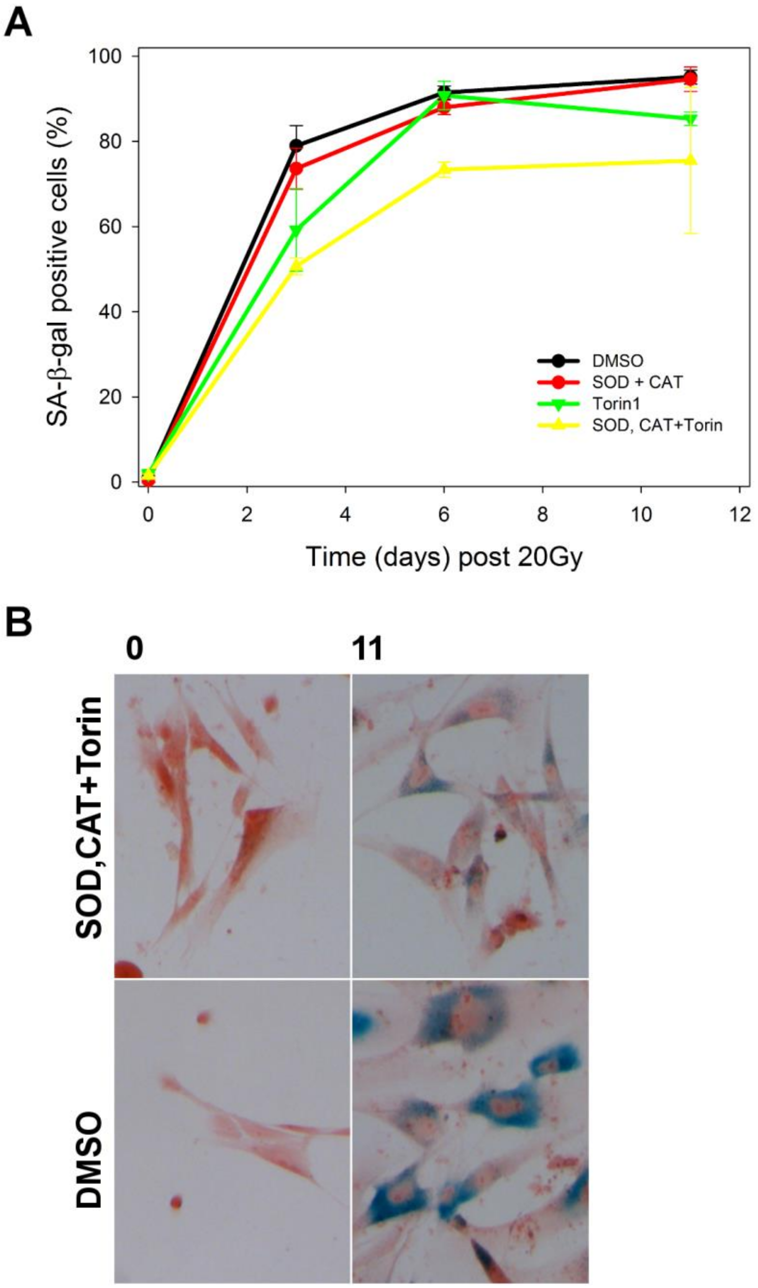

Figure S22. Analysis of senescence–associated b-galactosidase staining upon upon ROS-mTOR combined intervention.
(A) Cells were fixed at the indicated timepoints and assayed for senescence–associated b-galactosidase followed by counterstaining with nuclear fast red prior to imaging. An average of ~300 cells were counted per coverslip, with any blue cytoplasmic staining being considered positive. Data are n=2 ± SD. Combined intervention produced significantly lower positive cells at 3 and 6 days post irradiation relative to DMSO control ($P < 0.05$). (B) Example images of stained coverslips showing the decreased intensity of staining observed in treated cells.

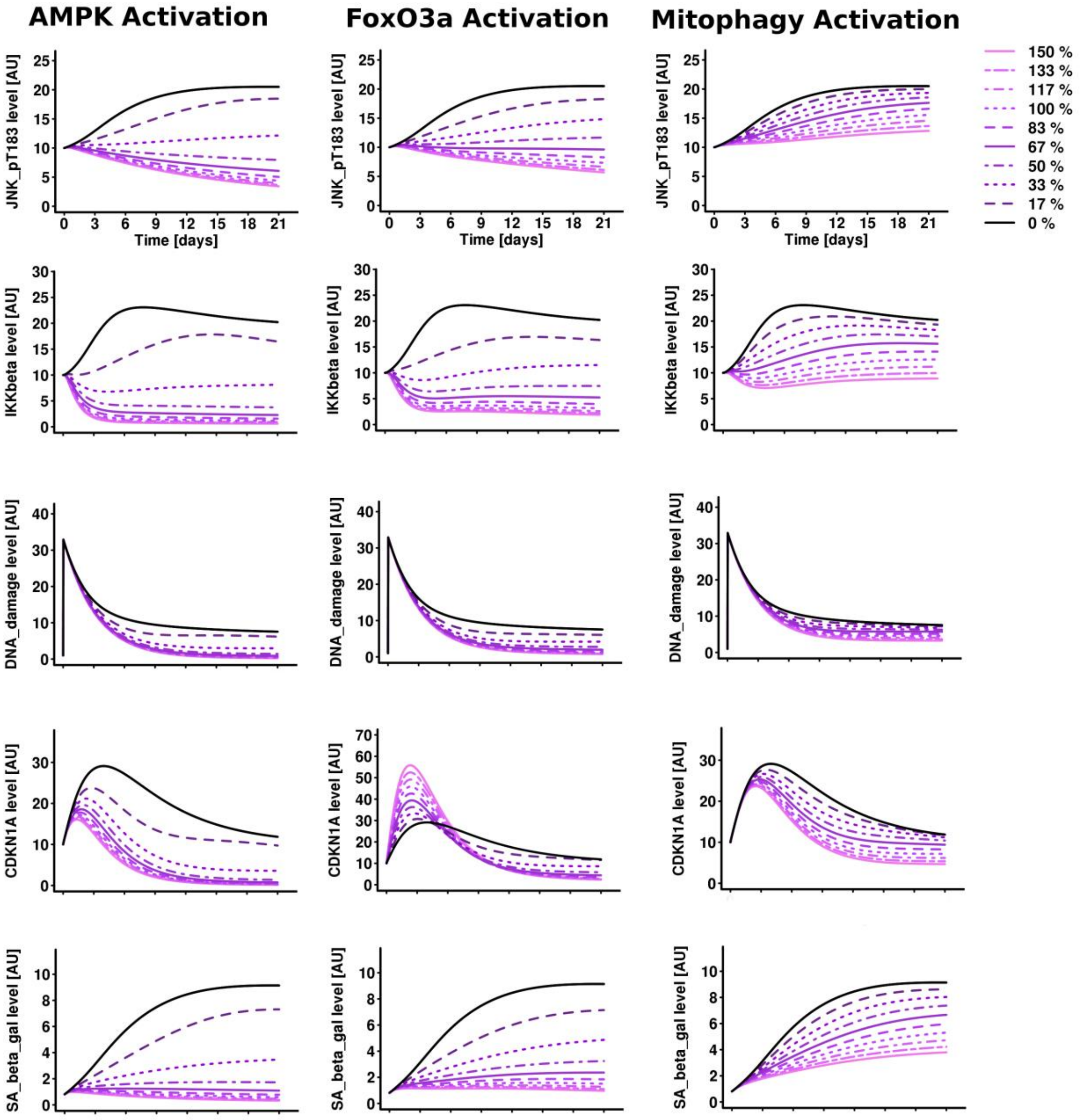

Figure S23. Additional readouts upon AMPK, FoxO3a or mitophagy simulated over-activation.
The model predicted a decrease in the DNA damage/oxidative stress response pathways upon over-activation of AMPK, FoxO3a or mitophagy. AMPK was predicted to achieve the strongest inhibition throughout the time course as compared to FoxO3a or mitophagy. Interestingly, an over-activation of FoxO3a was predicted to initially increase and then reduce CDKN1A levels as compared to the control (black line), suggesting differential regulation of cell cycle arrest over time.

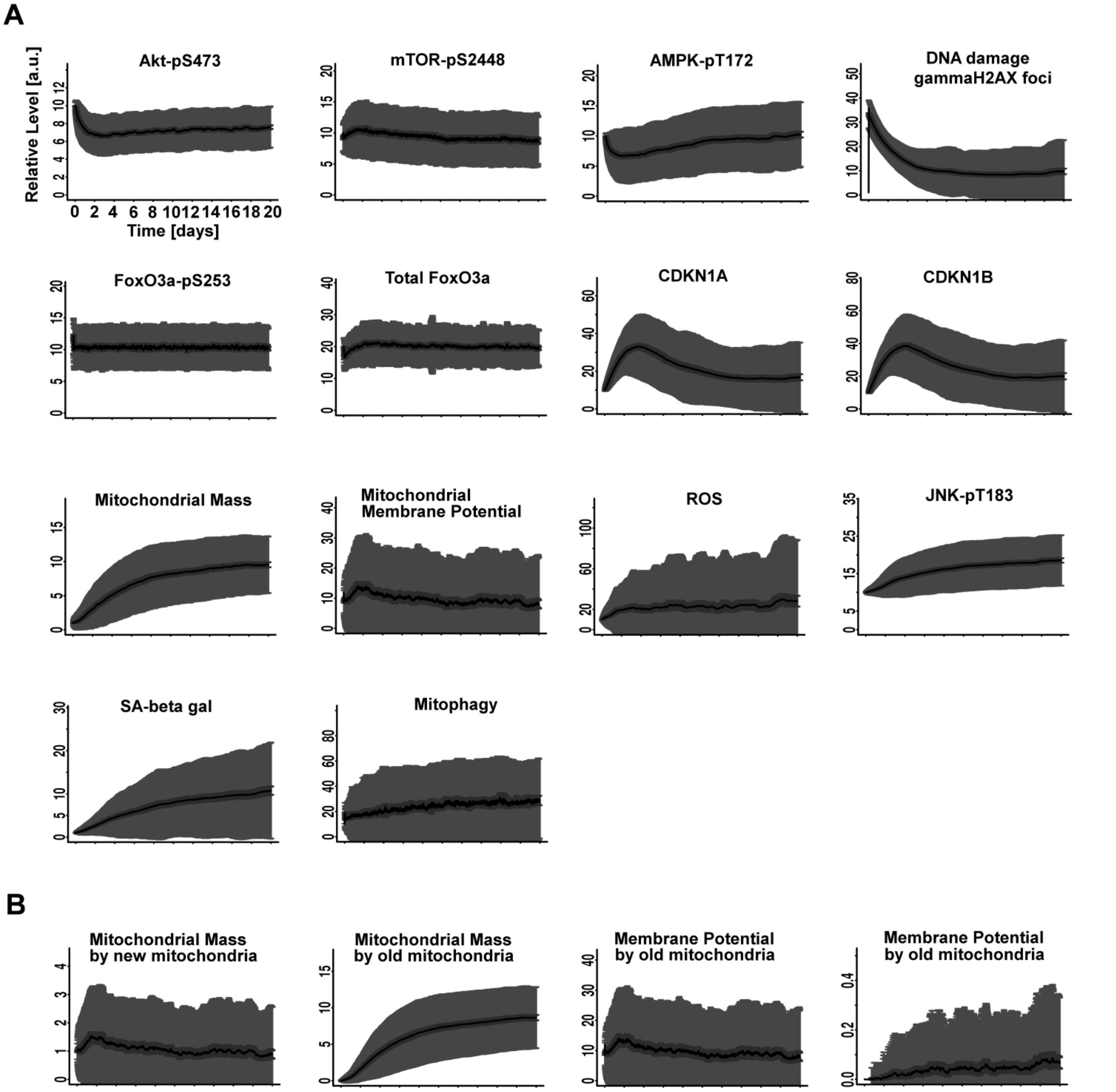

Figure S24. Model stochastic simulation showed increase stochasticity over time.
Model stochastic simulations up to 20 days graphically showed increased stochasticity for the oxidative stress/DNA damage signalling species. Moderate increased variance was also detected for the species Mitophagy and the species representing mitochondrial mass and membrane potential. Number of stochastic runs: 500; black line indicates the means, dark grey area indicates 95% confidence interval of the mean and grey area indicates a standard deviation. (A) Species associated to *in vitro* data (observable variables). (B) Mitochondrial internal states (derived variables).
